# Supplementary material for: Trajectories of childhood immune development and respiratory health relevant to asthma and allergy
Source: eLife. 2018 Oct 15;7:e35856. doi: 10.7554/eLife.35856 (PMC6221547; doi:10.7554/eLife.35856)
Supplement: Supplementary file 1. [file elife-35856-supp1.docx]

# Supplementary File 1

Table supplement 1: List of clustering features

| **Feature** | **Present?** | | |
| --- | --- | --- | --- |
|  | **CAS** | **MAAS** | **COAST** |
| **Sex** | Y | Y | Y |
| **Respiratory infection-related variables** |  |  |  |
| Frequency of upper respiratory illnesses (URIs) at ages 1, 2, 3 | Y | Y (1,3) | Y |
| Frequency of lower respiratory illnesses (LRIs) at ages 1, 2, 3 | Y | N | Y |
| Frequency of wheezy LRIs (wLRIs) at ages 1, 2, 3 | Y | Y (1,3) | Y |
| Frequency of febrile LRIs (fLRIs) at ages 1, 2, 3 | Y | N | Y |
| Number of URIs, LRIs, wLRIs, and fLRIs with respiratory syncytial virus (RSV) detected, at ages 1, 2, 3 | Y | N | Y |
| Number of URIs, LRIs, wLRIs, and fLRIs with influenza detected, at ages 1, 2, 3 | Y | N | Y |
| Number of LRIs, wLRIs, and fLRIs with human rhinovirus A (HRVA) detected, at ages 1, 2, 3 | Y | N | Y |
| Number of LRIs, wLRIs, and fLRIs with human rhinovirus B (HRVB) detected, at ages 1, 2, 3 | Y | N | Y |
| Number of LRIs, wLRIs, and fLRIs with human rhinovirus C (HRVC) detected, at ages 1, 2, 3 | Y | N | Y |
| **IgE variables** |  |  |  |
| Total IgE (kU/L), with log10 transformation, at age 6m, 1, 2 and 3 | Y | Y (1,3) | Y (1,2,3) |
| House dust mite (HDM)-specific IgE (kU/L), with log10 transformation, at age 6m, 1, 2 and 3 | Y | Y (1,3) | Y (1,2,3) |
| Cat-specific IgE (kU/L), with log10 transformation, at ages 6m, 1, 2 and 3 | Y | Y (1,3) | Y (1,2,3) |
| Peanut-specific IgE (kU/L), with log10 transformation, at ages 6m, 1, 2 and 3 | Y | N | Y (1,2,3) |
| Couch grass-specific IgE (kU/L), with log10 transformation, at ages 6m, 1, 2 and 3 | Y | N | N |
| Ryegrass-specific IgE (kU/L), with log10 transformation, at ages 6m, 1, 2 and 3 | Y | N | N |
| Mould-specific IgE (kU/L), with log10 transformation, at ages 6m, 1, 2 and 3 | Y | N | N |
| Phadiatop Infant IgE (kU/L), with log10 transformation, at ages 6m, 1, 2 and 3 | Y | N | N |
| **IgG4 variables** |  |  |  |
| HDM-specific IgG4 (μg/L), with log10 transformation, at ages 6m, 1, 2 and 3 | Y | Y (5*) | N |
| Cat-specific IgG4 (μg/L), with log10 transformation, at ages 6m, 1, 2 and 3 | Y | Y (5*) | N |
| Peanut-specific IgG4 (μg/L), with log10 transformation, at ages 6m, 1, 2 and 3 | Y | N | N |
| Couch grass-specific IgG4 (μg/L), with log10 transformation, at ages 6m, 1, 2 and 3 | Y | N | N |
| Ryegrass-specific IgG4 (μg/L), with log10 transformation, at ages 6m, 1, 2 and 3 | Y | N | N |
| Mould-specific IgG4 (μg/L), with log10 transformation, at ages 6m, 1, 2 and 3 | Y | N | N |
| Phadiatop Infant IgG4 (μg/L), with log10 transformation, at ages 6m, 1, 2 and 3 | Y | N | N |
| **IgG variables** |  |  |  |
| HDM-specific IgG (mg/L), with log10 transformation, at ages 1,2, and 3 | Y | Y (5*) | N |
| Cat-specific IgG (mg/L), with log10 transformation, at ages 1,2, and 3 | Y | Y (5*) | N |
| (Timothy) Grass-specific IgG (mg/L), with log10 transformation, at ages 1,2, and 3 | Y | N | N |
| **SPT variables** |  |  |  |
| Histamine-specific skin sensitisation or skin prick test (SPT) response, diameter of wheal (mm) at ages 6m, 2 | Y | N | N |
| HDM-specific SPT response, diameter of wheal (mm) at ages 6m, 2 | Y | Y (1*,3*) | N |
| Cat-specific SPT response, diameter of wheal (mm) at ages 6m, 2 | Y | Y (1*,3*) | N |
| Ryegrass-specific SPT response, diameter of wheal (mm) at ages 6m, 2 | Y | Y (1*,3*)^ | N |
| *Alternaria*-specific SPT response, diameter of wheal (mm) at ages 6m, 2 | Y | N | N |
| *Aspergillus*-specific SPT response, diameter of wheal (mm) at ages 6m, 2 | Y | Y (3*) | N |
| Cow’s milk-specific SPT response, diameter of wheal (mm) at ages 6m, 2 | Y | Y (1*,3*) | N |
| Egg white-specific SPT response, diameter of wheal (mm) at ages 6m, 2 | Y | Y (1*,3*) | N |
| **Family history of asthma or atopy** |  |  |  |
| Maternal and paternal history of atopy as determined by SPT ≥ 3mm to any allergen | Y | Y | Y |
| Maternal and paternal history of physician-diagnosed asthma | Y | Y | Y |
| Maternal and paternal history of physician-diagnosed atopic disease besides asthma (eczema, hayfever) | Y | Y | Y |
| **Other** |  |  |  |
| Child ever exposed to cigarette smoke at ages 1, 2 and 3 | Y | Y (1,3) | Y |
| Child ever attended childcare at ages 1, 2, and 3 | Y | Y (2,3) | Y |
| Child ever exposed to cat at ages 1, 2 and 3 | Y | Y | Y |
| Number of children (age<16) living in the same household at ages 1, 2, and 3 | Y | Y | Y |
| Number of children older than the proband, living in the same household at ages 0, 1, 2, and 3 | Y | Y | Y |
| Height (cm) at age 3 | Y | Y | Y |
| Weight (kg) at age 3 | Y | Y | Y |
| 25-Hydroxy Vitamin D (nmol/L) in mothers’s serum at 6 weeks postpartum | Y | N | N |
| Physician-diagnosed eczema or atopic dermatitis at ages 6m, 1, 2, and 3 | Y | Y (1,3) | Y |

*Used as surrogate measure for values at different timepoints. Age 5 values substituted for age 3; age 3 for age 2; age 1 for age 6m, as indicated. ^Mixed grass SPT used as surrogate for ryegrasss SPT. Blue and Y indicates feature present in dataset; yellow and Y indicates present, but only for some time-points (as indicated by numbers in brackets); red and N indicates feature absent from dataset.

Table supplement 2: Terminology used to describe groupings produced by various clustering and classification methods on different datasets

| **Dataset** | **Method** | **Terminology** | | |
| --- | --- | --- | --- | --- |
|  |  | **Cluster 1** | **Cluster 2** | **Cluster 3** |
| CAS (discovery) | npEM clustering for complete-case subset; npEM-based classification for low-missingness subset | CAS1 | CAS2 | CAS3 |
| MAAS (replication) | CAS npEM-based classification of MAAS, using only features common to MAAS and CAS | MAAS1 | MAAS2 | MAAS3 |
| COAST (replication) | CAS npEM-based classification of COAST, using only features common to COAST and CAS | COAST1 | COAST2 | COAST3 |

CAS = Childhood Asthma Study, Perth, Australia; COAST = Childhood Origins of Asthma Study, Wisconsin, US; MAAS = Manchester Asthma and Allergy Study, Manchester, UK; npEM = non-parametric expectation-maximisation mixture modelling

Table supplement 3: Comparison of selected variables (respiratory, immunological, clinical) in CAS clusters

1. **Clinical and demographic**

| **Variable** | **Age** | **CAS1 (N=88)** | **CAS2 (N=107)** | **CAS3 (N=22)** | **P-value** |  |  |  | **P-value (adj.)** |  |  |  | **Feature?** |
| --- | --- | --- | --- | --- | --- | --- | --- | --- | --- | --- | --- | --- | --- |
|  |  | **Prop. (95% CI)** | **Prop. (95% CI)** | **Prop. (95% CI)** | **Overall** | **1 vs. 2** | **1 vs. 3** | **2 vs. 3** | **Overall** | **1 vs. 2** | **1 vs. 3** | **2 vs. 3** |  |
| *Sex* |  |  |  |  |  |  |  |  |  |  |  |  |  |
| Male |  | 55% (44%-65%) | 51% (42%-61%) | 86% (71%-100%) | **7.3E-03** | 0.67 | **6.8E-03** | **3.7E-03** | 0.15 | 1 | 0.23 | 0.14 | **Yes** |
| *Maternal medical history* |  |  |  |  |  |  |  |  |  |  |  |  |  |
| Atopy |  | 74% (65%-83%) | 84% (77%-91%) | 82% (64%-99%) | 0.2 | 0.11 | 0.58 | 0.76 | 1 | 1 | 1 | 1 | No |
| Atopic eczema |  | 83% (75%-91%) | 81% (74%-89%) | 95% (86%-100%) | 0.28 | 0.85 | 0.19 | 0.12 | 1 | 1 | 1 | 1 | No |
| Asthma |  | 51% (40%-62%) | 41% (32%-51%) | 59% (37%-81%) | 0.19 | 0.19 | 0.63 | 0.16 | 1 | 1 | 1 | 1 | **Yes** |
| Atopic disease besides asthma |  | 72% (62%-81%) | 74% (65%-82%) | 77% (58%-96%) | 0.88 | 0.75 | 0.79 | 1 | 1 | 1 | 1 | 1 | **Yes** |
| *Paternal medical history* |  |  |  |  |  |  |  |  |  |  |  |  |  |
| Atopy |  | 74% (65%-83%) | 85% (78%-92%) | 91% (78%-100%) | *0.075* | *0.072* | 0.15 | 0.74 | 1 | 1 | 1 | 1 | No |
| Atopic eczema |  | 58% (47%-68%) | 77% (69%-85%) | 59% (37%-81%) | **0.01** | **5.0E-03** | 1 | 0.11 | 0.2 | 0.18 | 1 | 1 | No |
| Asthma |  | 22% (13%-30%) | 44% (35%-54%) | 23% (3.7%-42%) | **2.2E-03** | **1.3E-03** | 1 | *0.093* | *0.051* | *0.055* | 1 | 1 | **Yes** |
| Atopic disease besides asthma |  | 48% (37%-58%) | 54% (44%-63%) | 41% (19%-63%) | 0.49 | 0.47 | 0.64 | 0.35 | 1 | 1 | 1 | 1 | **Yes** |
| *Atopy-associated diseases* |  |  |  |  |  |  |  |  |  |  |  |  |  |
| Wheeze | 1 | 33% (23%-43%) | 30% (21%-39%) | 55% (32%-77%) | *0.092* | 0.76 | *0.084* | **0.046** | 1 | 1 | 1 | 1 | No |
|  | 2 | 30% (20%-39%) | 29% (20%-38%) | 59% (37%-81%) | **0.024** | 1 | **0.013** | **0.012** | 0.42 | 1 | 0.4 | 0.37 | No |
|  | 3 | 30% (20%-40%) | 23% (15%-31%) | 55% (32%-77%) | **0.015** | 0.32 | **0.044** | **7.6E-03** | 0.28 | 1 | 1 | 0.25 | No |
|  | 4 | 23% (14%-32%) | 20% (12%-28%) | 77% (58%-96%) | **9.3E-07** | 0.59 | **5.1E-06** | **5.2E-07** | **4.0E-05** | 1 | **3.9E-04** | **5.0E-05** | No |
|  | 5 | 25% (15%-35%) | 21% (13%-30%) | 76% (56%-96%) | **7.1E-06** | 0.59 | **2.6E-05** | **3.4E-06** | **2.6E-04** | 1 | **1.7E-03** | **2.8E-04** | No |
|  | 10 | 12% (3.4%-21%) | 18% (8.4%-27%) | 50% (24%-76%) | **3.1E-03** | 0.46 | **1.5E-03** | 0.011 | *0.069* | 1 | *0.063* | 0.34 | No |
| Asthma | 3 | 12% (4.8%-19%) | 3.8% (0.087%-7.5%) | 23% (3.7%-42%) | **8.2E-03** | **0.049** | 0.3 | **7.9E-03** | 0.17 | 1 | 1 | 0.26 | No |
|  | 4 | 11% (4.1%-18%) | 8.2% (2.7%-14%) | 36% (15%-58%) | **4.2E-03** | 0.61 | **8.3E-03** | **2.0E-03** | *0.091* | 1 | 0.27 | 0.08 | No |
|  | 5 | 15% (7%-23%) | 13% (5.9%-20%) | 52% (29%-76%) | **4.1E-04** | 0.83 | **7.7E-04** | **2.1E-04** | **0.011** | 1 | **0.034** | **0.011** | No |
|  | 10 | 10% (2.3%-18%) | 15% (6.1%-23%) | 56% (30%-81%) | **2.6E-04** | 0.59 | **1.8E-04** | **7.9E-04** | **7.2E-03** | 1 | **9.6E-03** | **0.035** | No |
| Allergic rhinoconjunctivitis | 5 | 30% (20%-40%) | 39% (29%-49%) | 76% (56%-96%) | **6.4E-04** | 0.21 | **2.7E-04** | **3.2E-03** | 0.017 | 1 | 0.014 | 0.12 | No |
| Eczema | 6m | 39% (28%-49%) | 45% (35%-54%) | 91% (78%-100%) | **2.4E-05** | 0.47 | **7.9E-06** | **9.0E-05** | **8.1E-04** | 1 | **5.8E-04** | **5.1E-03** | **Yes** |
|  | 1 | 34% (24%-44%) | 30% (21%-39%) | 82% (64%-99%) | **2.5E-05** | 0.54 | **7.2E-05** | **1.4E-05** | **8.4E-04** | 1 | **4.2E-03** | **9.6E-04** | **Yes** |
|  | 2 | 30% (20%-39%) | 31% (22%-40%) | 68% (47%-89%) | **2.7E-03** | 0.88 | **1.2E-03** | **1.5E-03** | *0.062* | 1 | *0.051* | *0.063* | **Yes** |
|  | 3 | 27% (18%-37%) | 25% (16%-33%) | 59% (37%-81%) | **7.1E-03** | 0.74 | **0.01** | **4.0E-03** | 0.15 | 1 | 0.32 | 0.15 | **Yes** |
|  | 4 | 26% (16%-35%) | 28% (19%-37%) | 59% (37%-81%) | **0.012** | 0.87 | **4.7E-03** | **0.011** | 0.23 | 1 | 0.17 | 0.34 | No |
|  | 5 | 28% (18%-37%) | 24% (16%-33%) | 71% (50%-92%) | **2.1E-04** | 0.73 | **3.3E-04** | **7.9E-05** | **6.1E-03** | 1 | **0.016** | **4.6E-03** | No |
| *Exposure to tobacco smoke* |  |  |  |  |  |  |  |  |  |  |  |  |  |
| Cigarette smoke exposure | 1 | 20% (12%-29%) | 20% (12%-27%) | 0% (0%-0%) | **0.041** | 1 | **0.021** | **0.023** | 0.65 | 1 | 0.6 | 0.64 | **Yes** |
|  | 2 | 22% (13%-30%) | 13% (6.6%-20%) | 4.5% (0%-14%) | 0.11 | 0.13 | *0.071* | 0.46 | 1 | 1 | 1 | 1 | **Yes** |
|  | 3 | 19% (11%-28%) | 19% (12%-27%) | 4.5% (0%-14%) | 0.24 | 1 | 0.11 | 0.12 | 1 | 1 | 1 | 1 | **Yes** |
|  | 4 | 16% (7.8%-24%) | 11% (4.9%-18%) | 4.5% (0%-14%) | 0.37 | 0.39 | 0.29 | 0.46 | 1 | 1 | 1 | 1 | No |
|  | 5 | 15% (7%-23%) | 16% (8.4%-23%) | 4.8% (0%-15%) | 0.49 | 1 | 0.29 | 0.3 | 1 | 1 | 1 | 1 | No |
|  | 10 | 12% (3.4%-21%) | 12% (3.9%-20%) | 5.6% (0%-17%) | 0.87 | 1 | 0.67 | 0.68 | 1 | 1 | 1 | 1 | No |
| *Exposure to childcare* |  |  |  |  |  |  |  |  |  |  |  |  |  |
| Childcare attendance | 1 | 27% (18%-37%) | 26% (18%-35%) | 36% (15%-58%) | 0.6 | 0.87 | 0.44 | 0.43 | 1 | 1 | 1 | 1 | **Yes** |
|  | 2 | 45% (34%-55%) | 44% (35%-54%) | 41% (19%-63%) | 0.96 | 1 | 0.81 | 0.82 | 1 | 1 | 1 | 1 | **Yes** |
|  | 3 | 54% (43%-64%) | 52% (43%-62%) | 73% (53%-93%) | 0.22 | 0.88 | 0.15 | 0.1 | 1 | 1 | 1 | 1 | **Yes** |
|  | 4 | 85% (78%-93%) | 86% (78%-93%) | 100% (100%-100%) | 0.15 | 1 | *0.066* | *0.07* | 1 | 1 | 1 | 1 | No |
| *Exposure to pets* |  |  |  |  |  |  |  |  |  |  |  |  |  |
| Exposure to cat | 1 | 36% (26%-47%) | 35% (25%-44%) | 23% (3.7%-42%) | 0.53 | 0.88 | 0.31 | 0.33 | 1 | 1 | 1 | 1 | **Yes** |
|  | 2 | 35% (25%-45%) | 36% (26%-45%) | 23% (3.7%-42%) | 0.55 | 1 | 0.32 | 0.32 | 1 | 1 | 1 | 1 | **Yes** |
|  | 3 | 30% (20%-40%) | 27% (18%-35%) | 14% (0%-29%) | 0.34 | 0.74 | 0.18 | 0.28 | 1 | 1 | 1 | 1 | **Yes** |
|  | 4 | 28% (18%-38%) | 26% (17%-35%) | 18% (0.68%-36%) | 0.7 | 0.74 | 0.42 | 0.59 | 1 | 1 | 1 | 1 | No |
|  | 5 | 30% (20%-40%) | 29% (19%-38%) | 14% (0%-31%) | 0.35 | 0.87 | 0.18 | 0.27 | 1 | 1 | 1 | 1 | No |
|  | 10 | 34% (22%-47%) | 26% (16%-37%) | 28% (4.9%-51%) | 0.61 | 0.34 | 0.78 | 1 | 1 | 1 | 1 | 1 | No |
| Exposure to dog | 10 | 38% (25%-51%) | 32% (21%-44%) | 22% (0.95%-43%) | 0.48 | 0.58 | 0.27 | 0.57 | 1 | 1 | 1 | 1 | No |
| Exposure to any furred pet | 10 | 62% (49%-75%) | 78% (68%-88%) | 78% (57%-99%) | 0.14 | *0.077* | 0.27 | 1 | 1 | 1 | 1 | 1 | No |
|  |  | **Mean (95% CI)** | **Mean (95% CI)** | **Mean (95% CI)** | **Overall** | **1 vs. 2** | **1 vs. 3** | **2 vs. 3** | **Overall** | **1 vs. 2** | **1 vs. 3** | **2 vs. 3** |  |
| *Anthropometry* |  |  |  |  |  |  |  |  |  |  |  |  |  |
| Height (cm) | 3 | 96 (95-97) | 97 (96-97) | 96 (95-97) | 0.54 | 0.3 | 0.91 | 0.49 | 1 | 1 | 1 | 1 | **Yes** |
|  | 4 | 104 (103-105) | 104 (103-105) | 103 (101-105) | 0.82 | 0.83 | 0.64 | 0.52 | 1 | 1 | 1 | 1 | No |
|  | 5 | 111 (110-112) | 111 (111-112) | 110 (108-112) | 0.57 | 0.7 | 0.44 | 0.29 | 1 | 1 | 1 | 1 | No |
| (m) | 10 | 1.4 (1.4-1.4) | 1.4 (1.4-1.4) | 1.4 (1.4-1.4) | 0.26 | 0.25 | 0.15 | 0.41 | 1 | 1 | 1 | 1 | No |
| Weight (kg) | 3 | 15 (15-16) | 15 (15-16) | 15 (14-16) | 0.8 | 0.61 | 0.78 | 0.6 | 1 | 1 | 1 | 1 | **Yes** |
|  | 4 | 17 (17-18) | 17 (17-18) | 18 (16-19) | 0.85 | 0.67 | 0.63 | 0.81 | 1 | 1 | 1 | 1 | No |
|  | 5 | 20 (19-21) | 20 (19-21) | 20 (18-21) | 0.77 | 0.49 | 1 | 0.7 | 1 | 1 | 1 | 1 | No |
|  | 10 | 37 (35-40) | 36 (34-38) | 35 (32-39) | 0.66 | 0.4 | 0.52 | 1 | 1 | 1 | 1 | 1 | No |
| BMI (kg/m^2^) | 3 | 16 (16-17) | 16 (16-17) | 16 (16-17) | 0.86 | 0.65 | 0.68 | 0.8 | 1 | 1 | 1 | 1 | No* |
|  | 4 | 16 (16-17) | 16 (16-16) | 17 (16-17) | 0.59 | 0.76 | 0.32 | 0.39 | 1 | 1 | 1 | 1 | No |
|  | 5 | 16 (16-16) | 16 (16-16) | 16 (15-17) | 0.71 | 0.56 | 0.48 | 0.67 | 1 | 1 | 1 | 1 | No |
|  | 10 | 18 (17-19) | 18 (17-18) | 18 (17-19) | 0.89 | 0.75 | 1 | 0.62 | 1 | 1 | 1 | 1 | No |
| *Household inhabitants* |  |  |  |  |  |  |  |  |  |  |  |  |  |
| Number of children in household | 1 | 1.8 (1.6-2) | 1.5 (1.4-1.7) | 1.8 (1.3-2.2) | **0.013** | **3.5E-03** | 0.57 | 0.21 | 0.25 | 0.13 | 1 | 1 | **Yes** |
|  | 2 | 2 (1.8-2.2) | 1.6 (1.5-1.8) | 2.1 (1.7-2.5) | **8.2E-04** | **5.7E-04** | 0.68 | **0.014** | **0.021** | **0.026** | 1 | 0.43 | **Yes** |
|  | 3 | 2.2 (2.1-2.4) | 2 (1.8-2.1) | 2.3 (1.9-2.7) | **0.035** | **0.022** | 0.81 | *0.082* | 0.57 | 0.62 | 1 | 1 | **Yes** |
|  | 4 | 2.4 (2.2-2.6) | 2.1 (1.9-2.2) | 2.5 (2.1-2.9) | **0.03** | **0.02** | 0.8 | *0.061* | 0.5 | 0.57 | 1 | 1 | No |
|  | 5 | 2.4 (2.2-2.6) | 2.1 (2-2.3) | 2.4 (2-2.8) | 0.11 | **0.04** | 0.59 | 0.38 | 1 | 1 | 1 | 1 | No |
|  | 10 | 2.6 (2.3-2.8) | 2.2 (2-2.4) | 2.3 (2-2.7) | **0.077** | **0.026** | 0.35 | 0.51 | 1 | 0.71 | 1 | 1 | No |
| Number of older children in household | 0 | 0.93 (0.72-1.1) | 0.53 (0.38-0.69) | 0.77 (0.32-1.2) | **4.5E-03** | **1.0E-03** | 0.37 | 0.25 | *0.097* | *0.043* | 1 | 1 | **Yes** |
|  | 1 | 0.84 (0.65-1) | 0.51 (0.36-0.67) | 0.77 (0.32-1.2) | **0.013** | **3.5E-03** | 0.57 | 0.21 | 0.25 | 0.13 | 1 | 1 | **Yes** |
|  | 2 | 0.85 (0.66-1) | 0.5 (0.34-0.65) | 0.77 (0.32-1.2) | **2.8E-03** | **6.5E-04** | 0.48 | 0.16 | *0.063* | **0.029** | 1 | 1 | **Yes** |
|  | 3 | 0.81 (0.6-1) | 0.5 (0.35-0.65) | 0.77 (0.32-1.2) | **0.032** | **0.01** | 0.74 | 0.19 | 0.53 | 0.32 | 1 | 1 | **Yes** |
|  | 4 | 0.88 (0.66-1.1) | 0.49 (0.34-0.65) | 0.57 (0.23-0.91) | **5.7E-03** | **1.4E-03** | 0.19 | 0.49 | 0.12 | *0.059* | 1 | 1 | No |
|  | 5 | 0.68 (0.5-0.85) | 0.39 (0.25-0.54) | 0.67 (0.23-1.1) | **0.016** | **5.1E-03** | 0.75 | 0.12 | 0.29 | 0.18 | 1 | 1 | No |
|  | 10 | 0.76 (0.58-0.95) | 0.44 (0.26-0.61) | 0.59 (0.22-0.95) | **0.028** | **7.5E-03** | 0.34 | 0.39 | 0.47 | 0.25 | 1 | 1 | No |
|  |  | **Geom. mean (95% CI)** | **Geom. mean (95% CI)** | **Geom. mean (95% CI)** | **Overall** | **1 vs. 2** | **1 vs. 3** | **2 vs. 3** | **Overall** | **1 vs. 2** | **1 vs. 3** | **2 vs. 3** |  |
| *Maternal Vitamin D* |  |  |  |  |  |  |  |  |  |  |  |  |  |
| Vitamin D (nmol/L) | 6wk | 76 (69-83) | 71 (66-77) | 69 (59-80) | 0.29 | 0.31 | 0.15 | 0.38 | 1 | 1 | 1 | 1 | **Yes** |
| *Child Vitamin D* |  |  |  |  |  |  |  |  |  |  |  |  |  |
| Vitamin D (nmol/L) | 0 | 26 (23-29) | 27 (24-30) | 26 (21-32) | 0.69 | 0.58 | 0.71 | 0.41 | 1 | 1 | 1 | 1 | No |
|  | 6m | 64 (59-69) | 64 (59-68) | 59 (50-70) | 0.66 | 0.68 | 0.38 | 0.51 | 1 | 1 | 1 | 1 | No |
|  | 1 | 60 (55-64) | 59 (55-63) | 59 (52-67) | 0.93 | 0.98 | 0.76 | 0.7 | 1 | 1 | 1 | 1 | No |
|  | 2 | 57 (54-61) | 58 (55-61) | 47 (40-55) | **0.012** | 0.82 | **5.4E-03** | **4.4E-03** | 0.23 | 1 | 0.19 | 0.16 | No |
|  | 3 | 53 (49-58) | 59 (56-62) | 51 (46-57) | *0.082* | 0.14 | 0.38 | **0.033** | 1 | 1 | 1 | 0.86 | No |
|  | 4 | 59 (56-62) | 57 (53-60) | 50 (45-56) | *0.082* | 0.51 | **0.023** | *0.077* | 1 | 1 | 0.64 | 1 | No |
|  | 5 | 89 (83-95) | 84 (79-89) | 77 (69-84) | *0.057* | 0.46 | **0.016** | *0.056* | 0.86 | 1 | 0.47 | 1 | No |
|  | 10 | 77 (72-82) | 78 (73-83) | 65 (56-75) | **0.039** | 0.86 | **0.024** | **0.012** | 0.63 | 1 | 0.67 | 0.37 | No |
| “Deseasonalised” Vitamin D (nmol/L) | 0 | 26 (23-29) | 27 (25-30) | 26 (21-31) | 0.46 | 0.4 | 0.6 | 0.26 | 1 | 1 | 1 | 1 | No |
|  | 6m | 65 (60-69) | 64 (60-68) | 60 (53-68) | 0.46 | 0.61 | 0.21 | 0.36 | 1 | 1 | 1 | 1 | No |
|  | 1 | 60 (57-64) | 60 (56-63) | 59 (51-68) | 0.91 | 0.89 | 0.67 | 0.71 | 1 | 1 | 1 | 1 | No |
|  | 2 | 57 (54-61) | 58 (56-61) | 48 (40-56) | **0.032** | 0.77 | **0.011** | **0.015** | 0.53 | 1 | 0.34 | 0.45 | No |
|  | 3 | 52 (48-57) | 60 (57-62) | 51 (45-58) | **0.018** | *0.052* | 0.27 | **9.8E-03** | 0.32 | 1 | 1 | 0.31 | No |
|  | 4 | 59 (56-62) | 57 (54-60) | 51 (44-59) | 0.31 | 0.52 | 0.13 | 0.26 | 1 | 1 | 1 | 1 | No |
|  | 5 | 88 (83-94) | 85 (81-89) | 80 (73-87) | 0.27 | 0.57 | 0.14 | 0.16 | 1 | 1 | 1 | 1 | No |
|  | 10 | 76 (71-81) | 78 (74-83) | 69 (60-80) | 0.21 | 0.44 | 0.23 | 0.074 | 1 | 1 | 1 | 1 | No |

BMI = body mass index; feature? = whether variable was used as a clustering feature or not; geom. mean = geometric mean; P-value (adj.) = adjusted P-values (Benjamini-Yekutieli method); prop. = proportion. For categorical variables, associations were tested using Fisher exact test; for continuous variables, Kruskal-Wallis and Mann-Whitney-Wilcoxon. Bold text indicates statistical significance (*p*<0.05); italics indicate near-significance (*p*<0.10). *Not used as clustering feature, as BMI is a derived variable. Height and weight at age 3 were used instead.

1. **Immunological (antibodies)**

| **Variable** | **Age** | **CAS1 (N=88)** | **CAS2 (N=107)** | **CAS3 (N=22)** | **P-value** |  |  |  | **P-value (adj.)** |  |  |  | **Feature?** |
| --- | --- | --- | --- | --- | --- | --- | --- | --- | --- | --- | --- | --- | --- |
|  |  | **Geom. mean (95% CI)** | **Geom. mean (95% CI)** | **Geom. mean (95% CI)** | **Overall** | **1 vs. 2** | **1 vs. 3** | **2 vs. 3** | **Overall** | **1 vs. 2** | **1 vs. 3** | **2 vs. 3** |  |
| *Total antibody* |  |  |  |  |  |  |  |  |  |  |  |  |  |
| IgE (kU/L) | 6m | 1.2 (0.69-2) | 2.2 (1.4-3.6) | 21 (12-35) | **1.2E-07** | **0.044** | **6.7E-08** | **2.2E-06** | **5.9E-06** | 1 | **8.1E-06** | **1.9E-04** | **Yes** |
|  | 1 | 0.6 (0.29-1.3) | 2 (1.1-3.7) | 43 (17-109) | **2.0E-09** | **0.019** | **4.3E-09** | **5.3E-08** | **1.3E-07** | 0.55 | **6.8E-07** | **6.6E-06** | **Yes** |
|  | 2 | 6.6 (3.5-12) | 17 (12-25) | 187 (131-267) | **1.2E-11** | **0.044** | **4.2E-11** | **1.4E-10** | **1.4E-09** | 1 | **1.2E-08** | **3.6E-08** | **Yes** |
|  | 3 | 18 (13-27) | 28 (22-35) | 267 (177-401) | **1.3E-11** | 0.11 | **2.8E-11** | **8.9E-11** | **1.4E-09** | 1 | **8.3E-09** | **2.5E-08** | **Yes** |
|  | 4 | 20 (13-29) | 35 (26-47) | 345 (212-563) | **2.8E-10** | *0.064* | **2.0E-10** | **8.5E-09** | **2.2E-08** | 1 | **4.6E-08** | **1.2E-06** | No |
|  | 5 | 35 (23-55) | 60 (46-80) | 451 (278-731) | **2.2E-08** | *0.096* | **1.9E-08** | **1.5E-07** | **1.2E-06** | 1 | **2.6E-06** | **1.7E-05** | No |
|  | 10 | 85 (46-154) | 150 (103-217) | 800 (405-1.6E+03) | **1.4E-04** | 0.11 | **1.3E-04** | **2.8E-04** | **4.2E-03** | 1 | **7.1E-03** | **0.014** | No |
| *HDM antibody* |  |  |  |  |  |  |  |  |  |  |  |  |  |
| IgE (kU/L) | 6m | 0.018 (0.016-0.02) | 0.019 (0.016-0.022) | 0.033 (0.019-0.059) | **1.9E-03** | 0.47 | **7.9E-04** | **4.2E-03** | **0.045** | 1 | **0.035** | 0.15 | **Yes** |
|  | 1 | 0.019 (0.017-0.023) | 0.019 (0.016-0.022) | 0.26 (0.075-0.93) | **1.3E-09** | 0.47 | **2.5E-07** | **4.5E-09** | **9.1E-08** | 1 | **2.6E-05** | **7.1E-07** | **Yes** |
|  | 2 | 0.024 (0.019-0.031) | 0.042 (0.029-0.06) | 7.1 (2.7-19) | **2.6E-16** | *0.078* | **2.5E-15** | **3.5E-13** | **8.0E-14** | 1 | **2.6E-12** | **2.4E-10** | **Yes** |
|  | 3 | 0.043 (0.029-0.064) | 0.064 (0.04-0.1) | 23 (7.5-68) | **1.5E-13** | 0.49 | **8.8E-13** | **2.4E-12** | **2.2E-11** | 1 | **4.9E-10** | **1.0E-09** | **Yes** |
|  | 4 | 0.057 (0.036-0.09) | 0.2 (0.11-0.35) | 30 (8.9-103) | **3.7E-11** | **2.8E-03** | **2.0E-10** | **1.7E-08** | **3.5E-09** | 0.11 | **4.6E-08** | **2.3E-06** | No |
|  | 5 | 0.072 (0.041-0.13) | 0.23 (0.12-0.45) | 31 (7.8-127) | **4.2E-09** | **0.015** | **3.8E-09** | **5.1E-07** | **2.6E-07** | 0.45 | **6.1E-07** | **5.0E-05** | No |
|  | 10 | 0.37 (0.17-0.8) | 1.3 (0.51-3.4) | 52 (19-144) | **2.9E-06** | *0.068* | **5.7E-07** | **9.7E-05** | **1.1E-04** | 1 | **5.4E-05** | **5.5E-03** | No |
| IgG (mg/L) | 1 | 0.21 (0.2-0.23) | 0.23 (0.21-0.25) | 0.29 (0.21-0.39) | **0.042** | 0.34 | **0.012** | *0.07* | 0.66 | 1 | 0.37 | 1 | **Yes** |
|  | 2 | 0.32 (0.27-0.37) | 0.49 (0.41-0.59) | 0.89 (0.57-1.4) | **1.9E-06** | **2.1E-04** | **3.8E-06** | **7.0E-03** | **7.8E-05** | **0.011** | **3.0E-04** | 0.24 | **Yes** |
|  | 3 | 0.24 (0.22-0.26) | 0.31 (0.26-0.36) | 0.88 (0.51-1.5) | **2.5E-07** | **0.023** | **8.5E-08** | **3.7E-05** | **1.2E-05** | 0.64 | **1.0E-05** | **2.3E-03** | **Yes** |
|  | 5 | 0.55 (0.42-0.7) | 0.59 (0.46-0.74) | 1.7 (0.88-3.3) | **1.5E-03** | **0.67** | **6.4E-04** | **9.0E-04** | **0.036** | 1 | **0.029** | **0.039** | No |
|  | 10 | 1.6 (1.3-1.9) | 2.1 (1.8-2.5) | 2.8 (1.9-4.2) | **1.0E-02** | **0.023** | **0.011** | 0.18 | 0.2 | 0.64 | 0.34 | 1 | No |
| IgG4 (μg/L) | 6m | 1.5E-04 (1.5E-04-1.5E-04) | 1.7E-04 (1.3E-04-2.1E-04) | 4.6E-04 (9.0E-05-2.4E-03) | **4.9E-03** | 0.37 | **5.2E-03** | **0.024** | 0.1 | 1 | 0.18 | 0.67 | **Yes** |
|  | 1 | 1.5E-04 (1.5E-04-1.5E-04) | 6.9E-04 (3.2E-04-1.5E-03) | 0.081 (4.6E-03-1.4) | **1.8E-10** | **5.2E-04** | **6.6E-12** | **2.2E-05** | **1.5E-08** | **0.024** | **2.5E-09** | **1.4E-03** | **Yes** |
|  | 2 | 3.4E-04 (1.8E-04-6.6E-04) | 4.8 (1.7-13) | 61 (8.9-419) | **1.8E-25** | **1.5E-22** | **8.6E-18** | **9.8E-05** | **5.0E-22** | **1.4E-18** | **2.0E-14** | **5.5E-03** | **Yes** |
|  | 3 | 7.7E-04 (3.1E-04-1.9E-03) | 35 (18-71) | 198 (46-859) | **2.9E-29** | **8.2E-26** | **1.0E-16** | **1.8E-06** | **1.6E-25** | **1.6E-21** | **1.6E-13** | **1.6E-04** | **Yes** |
|  | 4 | 0.01 (2.6E-03-0.041) | 16 (5.5-44) | 389 (228-664) | **3.1E-18** | **2.0E-13** | **2.2E-12** | **1.5E-05** | **1.9E-15** | **1.5E-10** | **9.7E-10** | **1.0E-03** | No |
|  | 5 | 2 (0.48-8.1) | 168 (111-256) | 539 (317-917) | **1.1E-15** | **1.3E-12** | **1.0E-08** | **1.9E-04** | **2.8E-13** | **6.5E-10** | **1.4E-06** | **0.01** | No |
| Non-IgG4 IgG (mg/L) | 1 | 0.21 (0.2-0.23) | 0.23 (0.21-0.25) | 0.28 (0.21-0.39) | **0.042** | 0.34 | **0.012** | *0.07* | 0.66 | 1 | 0.37 | 1 | No* |
|  | 2 | 0.31 (0.26-0.37) | 0.46 (0.38-0.54) | 0.67 (0.44-1) | **5.7E-05** | **6.7E-04** | **8.6E-05** | *0.05* | **1.8E-03** | **0.03** | **4.9E-03** | 1 | No* |
|  | 3 | 0.24 (0.22-0.26) | 0.29 (0.25-0.33) | 0.59 (0.35-1) | **2.3E-04** | *0.061* | **5.8E-05** | **3.5E-03** | **6.5E-03** | 1 | **3.5E-03** | 0.13 | No* |
|  | 5 | 0.52 (0.41-0.66) | 0.47 (0.38-0.59) | 1.2 (0.62-2.3) | **9.4E-03** | 0.57 | **7.1E-03** | **3.3E-03** | 0.19 | 1 | 0.24 | 0.13 | No |
| IgG:IgE (mg/L:mg/L) | 1 | 4.6E+03 (3.9E+03-5.4E+03) | 5.1E+03 (4.3E+03-6.0E+03) | 452 (120-1.7E+03) | **2.9E-06** | 0.15 | **3.7E-05** | **4.7E-06** | **1.1E-04** | 1 | **2.3E-03** | **3.7E-04** | No* |
|  | 2 | 5.4E+03 (4.0E+03-7.2E+03) | 5.0E+03 (3.3E+03-7.6E+03) | 52 (22-122) | **4.9E-11** | 0.15 | **1.4E-11** | **5.4E-10** | **4.5E-09** | 1 | **4.7E-09** | **1.1E-07** | No* |
|  | 3 | 2.3E+03 (1.5E+03-3.4E+03) | 2.0E+03 (1.3E+03-3.2E+03) | 16 (6.5-41) | **3.9E-11** | 0.96 | **2.0E-11** | **2.7E-10** | **3.7E-09** | 1 | **6.2E-09** | **5.9E-08** | No* |
|  | 5 | 3.8E+03 (2.2E+03-6.5E+03) | 1.2E+03 (608-2.4E+03) | 23 (8.4-60) | **2.2E-08** | **0.029** | **8.8E-09** | **1.8E-06** | **1.2E-06** | 0.77 | **1.3E-06** | **1.6E-04** | No |
|  | 10 | 1.7E+03 (830-3.5E+03) | 656 (263-1.6E+03) | 22 (9.3-54) | **3.0E-05** | 0.18 | **3.0E-06** | **3.7E-04** | **9.9E-04** | 1 | **2.5E-04** | **0.018** | No |
| IgG4:IgE (μg/L:μg/L) | 6m | 3.5E-03 (3.1E-03-4.0E-03) | 3.6E-03 (2.8E-03-4.8E-03) | 5.9E-03 (9.3E-04-0.037) | *0.06* | 0.61 | **0.026** | *0.058* | 0.89 | 1 | 0.71 | 1 | No* |
|  | 1 | 3.2E-03 (2.8E-03-3.8E-03) | 0.015 (6.9E-03-0.033) | 0.13 (7.3E-03-2.2) | **4.2E-03** | **9.2E-03** | **3.6E-03** | 0.15 | *0.091* | 0.3 | 0.13 | 1 | No* |
|  | 2 | 5.8E-03 (3.0E-03-0.011) | 53 (18-154) | 3.6 (0.56-23) | **2.2E-20** | **2.1E-19** | **8.8E-09** | **4.3E-04** | **2.4E-17** | **8.0E-16** | **1.3E-06** | **0.02** | No* |
|  | 3 | 7.4E-03 (2.8E-03-0.02) | 231 (101-528) | 3.7 (0.74-18) | **1.3E-23** | **9.4E-22** | **1.4E-07** | **1.1E-06** | **2.4E-20** | **6.0E-18** | **1.6E-05** | **1.0E-04** | No* |
|  | 4 | 0.076 (0.021-0.28) | 33 (11-103) | 5.3 (1.8-16) | **3.4E-09** | **7.7E-09** | **1.6E-03** | **1.5E-03** | **2.2E-07** | **1.1E-06** | *0.066* | *0.063* | No |
|  | 5 | 11 (3-44) | 307 (148-634) | 7.1 (1.9-27) | **6.3E-06** | **3.1E-04** | 0.13 | **1.2E-05** | **2.3E-04** | **0.015** | 1 | **8.3E-04** | No |
| *Cat antibody* |  |  |  |  |  |  |  |  |  |  |  |  |  |
| IgE (kU/L) | 6m | 0.018 (0.016-0.021) | 0.021 (0.018-0.024) | 0.029 (0.018-0.048) | **0.029** | 0.21 | **7.3E-03** | *0.075* | 0.48 | 1 | 0.24 | 1 | **Yes** |
|  | 1 | 0.017 (0.015-0.019) | 0.017 (0.015-0.018) | 0.05 (0.026-0.096) | **1.7E-08** | 0.34 | **6.6E-06** | **3.9E-08** | **9.7E-07** | 1 | **4.9E-04** | **5.0E-06** | **Yes** |
|  | 2 | 0.018 (0.016-0.021) | 0.02 (0.017-0.023) | 0.072 (0.034-0.15) | **3.2E-09** | 0.66 | **4.6E-08** | **8.3E-08** | **2.1E-07** | 1 | **5.7E-06** | **1.0E-05** | **Yes** |
|  | 3 | 0.021 (0.017-0.026) | 0.019 (0.016-0.022) | 0.09 (0.038-0.21) | **2.2E-09** | 0.6 | **5.6E-07** | **6.8E-09** | **1.5E-07** | 1 | **5.3E-05** | **1.0E-06** | **Yes** |
|  | 4 | 0.017 (0.015-0.018) | 0.023 (0.018-0.029) | 0.13 (0.05-0.32) | **9.2E-12** | *0.071* | **1.3E-11** | **1.4E-07** | **1.1E-09** | 1 | **4.5E-09** | **1.6E-05** | No |
|  | 5 | 0.018 (0.015-0.021) | 0.027 (0.02-0.036) | 0.21 (0.075-0.61) | **2.4E-10** | **0.022** | **1.0E-10** | **1.2E-06** | **1.9E-08** | 0.62 | **2.7E-08** | **1.1E-04** | No |
|  | 10 | 0.05 (0.029-0.084) | 0.056 (0.033-0.095) | 1.4 (0.38-5.1) | **4.1E-06** | 0.71 | **3.8E-06** | **6.9E-06** | **1.5E-04** | 1 | **3.0E-04** | **5.1E-04** | No |
| IgG (mg/L) | 1 | 0.2 (0.2-0.21) | 0.23 (0.21-0.25) | 0.24 (0.19-0.3) | **0.024** | **0.015** | **5.5E-03** | 0.49 | 0.42 | 0.45 | 0.19 | 1 | **Yes** |
|  | 2 | 0.28 (0.24-0.32) | 0.37 (0.31-0.45) | 0.35 (0.24-0.51) | **0.023** | **6.0E-03** | 0.19 | 0.71 | 0.4 | 0.21 | 1 | 1 | **Yes** |
|  | 3 | 0.22 (0.21-0.24) | 0.3 (0.26-0.35) | 0.39 (0.26-0.6) | **1.9E-03** | **8.6E-03** | **5.2E-04** | 0.14 | **0.045** | 0.28 | **0.024** | 1 | **Yes** |
|  | 5 | 0.48 (0.38-0.62) | 0.52 (0.41-0.66) | 0.92 (0.54-1.6) | *0.06* | 0.7 | **0.021** | **0.037** | 0.89 | 1 | 0.6 | 0.94 | No |
|  | 10 | 0.72 (0.54-0.95) | 0.89 (0.72-1.1) | 1.1 (0.63-1.9) | 0.3 | 0.18 | 0.24 | 0.72 | 1 | 1 | 1 | 1 | No |
| IgG4 (μg/L) | 6m | 2.3E-04 (1.4E-04-3.9E-04) | 4.8E-04 (2.4E-04-9.8E-04) | 1.8E-03 (1.6E-04-0.019) | **0.048** | *0.099* | **0.012** | 0.21 | 0.74 | 1 | 0.37 | 1 | **Yes** |
|  | 1 | 7.5E-04 (3.2E-04-1.8E-03) | 0.032 (9.5E-03-0.11) | 1.1 (0.066-18) | **2.2E-08** | **3.4E-06** | **1.6E-08** | **0.017** | **1.2E-06** | **2.8E-04** | **2.2E-06** | 0.5 | **Yes** |
|  | 2 | 0.055 (0.015-0.21) | 68 (36-126) | 33 (3.5-317) | **1.3E-19** | **2.6E-19** | **3.6E-08** | **0.34** | **1.2E-16** | **8.3E-16** | **4.7E-06** | 1 | **Yes** |
|  | 3 | 0.32 (0.079-1.3) | 110 (58-210) | 285 (182-445) | **7.0E-19** | **3.2E-17** | **1.1E-09** | *0.082* | **4.9E-16** | **6.8E-14** | **2.0E-07** | 1 | **Yes** |
|  | 4 | 1.3 (0.31-5.5) | 117 (56-245) | 199 (44-895) | **3.0E-14** | **1.9E-12** | **2.8E-08** | *0.054* | **5.9E-12** | **8.8E-10** | **3.8E-06** | 1 | No |
|  | 5 | 32 (10-100) | 449 (377-535) | 298 (48-1.8E+03) | **2.7E-11** | **5.1E-10** | **2.1E-06** | **0.035** | **2.7E-09** | **1.1E-07** | **1.8E-04** | 0.9 | No |
| Non-IgG4 IgG (mg/L) | 1 | 0.2 (0.2-0.21) | 0.23 (0.21-0.24) | 0.24 (0.19-0.3) | **0.025** | **0.015** | **5.5E-03** | 0.49 | 0.43 | 0.45 | 0.19 | 1 | No* |
|  | 2 | 0.27 (0.24-0.32) | 0.33 (0.28-0.38) | 0.28 (0.21-0.37) | 0.14 | *0.056* | 0.79 | 0.37 | 1 | 1 | 1 | 1 | No* |
|  | 3 | 0.22 (0.21-0.24) | 0.26 (0.23-0.29) | 0.33 (0.22-0.48) | **0.017** | 0.12 | **4.1E-03** | *0.078* | 0.31 | 1 | 0.15 | 1 | No* |
|  | 5 | 0.43 (0.34-0.54) | 0.37 (0.3-0.46) | 0.51 (0.29-0.88) | 0.54 | 0.48 | 0.53 | 0.33 | 1 | 1 | 1 | 1 | No |
| IgG:IgE (mg/L:mg/L) | 1 | 4.9E+03 (4.4E+03-5.5E+03) | 5.7E+03 (5.0E+03-6.4E+03) | 2.0E+03 (1.0E+03-4.1E+03) | **3.1E-05** | **0.03** | **8.4E-04** | **9.6E-05** | **1.0E-03** | 0.79 | **0.037** | **5.5E-03** | No* |
|  | 2 | 6.2E+03 (5.0E+03-7.7E+03) | 8.0E+03 (6.4E+03-9.9E+03) | 2.0E+03 (982-4.1E+03) | **3.0E-06** | **0.043** | **2.0E-05** | **7.1E-06** | **1.2E-04** | 1 | **1.3E-03** | **5.2E-04** | No* |
|  | 3 | 4.5E+03 (3.6E+03-5.7E+03) | 6.6E+03 (5.4E+03-8.1E+03) | 1.8E+03 (724-4.6E+03) | **2.8E-04** | *0.051* | **3.6E-03** | **2.8E-04** | **7.7E-03** | 1 | 0.13 | **0.014** | No* |
|  | 5 | 1.1E+04 (8.4E+03-1.4E+04) | 8.3E+03 (6.0E+03-1.2E+04) | 1.8E+03 (686-4.7E+03) | **1.6E-03** | 0.5 | **3.9E-04** | **2.2E-03** | **0.038** | 1 | **0.019** | *0.087* | No |
|  | 10 | 6.0E+03 (3.6E+03-1.0E+04) | 6.6E+03 (3.9E+03-1.1E+04) | 326 (110-960) | **2.3E-05** | 0.72 | **7.1E-06** | **2.5E-05** | **7.8E-04** | 1 | **5.2E-04** | **1.6E-03** | No |
| IgG4:IgE (μg/L:μg/L) | 6m | 5.4E-03 (3.2E-03-9.2E-03) | 9.7E-03 (4.7E-03-0.02) | 0.025 (1.9E-03-0.33) | 0.72 | 0.94 | 0.41 | 0.51 | 1 | 1 | 1 | 1 | No* |
|  | 1 | 0.018 (7.5E-03-0.044) | 0.82 (0.24-2.8) | 9.1 (0.48-171) | **7.8E-06** | **4.4E-06** | **4.4E-04** | 0.54 | **2.8E-04** | **3.5E-04** | **0.021** | 1 | No* |
|  | 2 | 1.3 (0.33-4.8) | 1.4E+03 (752-2.7E+03) | 191 (20-1.9E+03) | **7.7E-18** | **1.6E-18** | **5.2E-04** | **0.011** | **4.3E-15** | **4.4E-15** | **0.024** | 0.34 | No* |
|  | 3 | 6.5 (1.5-28) | 2.4E+03 (1.2E+03-4.8E+03) | 1.3E+03 (578-3.0E+03) | **4.9E-15** | **8.1E-16** | **2.3E-03** | **0.018** | **1.1E-12** | **9.1E-13** | *0.091* | 0.52 | No* |
|  | 4 | 33 (8-138) | 2.1E+03 (1.0E+03-4.6E+03) | 656 (151-2.8E+03) | **1.9E-09** | **8.9E-10** | 0.44 | **8.4E-04** | **1.3E-07** | **1.6E-07** | 1 | **0.037** | No |
|  | 5 | 732 (235-2.3E+03) | 7.0E+03 (5.0E+03-9.8E+03) | 580 (104-3.2E+03) | **5.7E-07** | **7.0E-06** | *0.055* | **9.2E-05** | **2.5E-05** | **5.2E-04** | 1 | **5.2E-03** | No |
| *Peanut antibody* |  |  |  |  |  |  |  |  |  |  |  |  |  |
| IgE (kU/L) | 6m | 0.024 (0.019-0.03) | 0.03 (0.024-0.037) | 0.21 (0.079-0.58) | **1.0E-06** | 0.11 | **4.0E-07** | **1.6E-05** | **4.3E-05** | 1 | **4.0E-05** | **1.1E-03** | **Yes** |
|  | 1 | 0.021 (0.017-0.025) | 0.024 (0.02-0.03) | 0.54 (0.19-1.5) | **8.9E-12** | 0.34 | **8.5E-11** | **5.6E-10** | **1.1E-09** | 1 | **2.4E-08** | **1.1E-07** | **Yes** |
|  | 2 | 0.024 (0.02-0.029) | 0.025 (0.021-0.03) | 0.58 (0.24-1.4) | **4.2E-14** | 0.56 | **7.3E-13** | **1.2E-12** | **7.8E-12** | 1 | **4.2E-10** | **6.3E-10** | **Yes** |
|  | 3 | 0.021 (0.017-0.026) | 0.018 (0.016-0.021) | 0.42 (0.14-1.3) | **3.8E-17** | 0.75 | **4.7E-12** | **9.8E-15** | **1.7E-14** | 1 | **1.9E-09** | **9.8E-12** | **Yes** |
|  | 4 | 0.019 (0.016-0.022) | 0.022 (0.018-0.027) | 0.37 (0.11-1.2) | **4.4E-13** | 0.17 | **6.4E-12** | **8.9E-10** | **6.1E-11** | 1 | **2.4E-09** | **1.6E-07** | No |
|  | 5 | 0.027 (0.02-0.037) | 0.034 (0.024-0.047) | 0.73 (0.19-2.8) | **6.9E-09** | 0.3 | **7.9E-09** | **2.2E-07** | **4.2E-07** | 1 | **1.2E-06** | **2.3E-05** | No |
|  | 10 | 0.085 (0.052-0.14) | 0.072 (0.045-0.11) | 0.78 (0.25-2.4) | **1.5E-04** | 0.58 | **2.6E-04** | **3.9E-05** | **4.5E-03** | 1 | **0.013** | **2.4E-03** | No |
| IgG4 (μg/L) | 6m | 1.5E-04 (1.5E-04-1.5E-04) | 1.5E-04 (1.5E-04-1.5E-04) | 8.1E-04 (1.2E-04-5.6E-03) | **2.1E-06** | NA | **5.7E-04** | **1.5E-04** | **8.5E-05** | NA | **0.026** | **8.2E-03** | **Yes** |
|  | 1 | 2.0E-04 (1.3E-04-2.9E-04) | 2.7E-03 (9.5E-04-7.7E-03) | 0.53 (0.023-12) | **3.3E-10** | **4.4E-05** | **3.8E-12** | **1.3E-04** | **2.5E-08** | **2.7E-03** | **1.5E-09** | **7.1E-03** | **Yes** |
|  | 2 | 2.9E-03 (8.5E-04-9.6E-03) | 4.6 (1.3-16) | 20 (1.4-280) | **4.9E-12** | **1.9E-11** | **5.7E-08** | 0.33 | **5.9E-10** | **6.0E-09** | **7.0E-06** | 1 | **Yes** |
|  | 3 | 5.3E-03 (1.4E-03-0.021) | 27 (8.8-85) | 77 (6.1-953) | **3.9E-14** | **1.7E-13** | **2.0E-08** | 0.2 | **7.5E-12** | **1.3E-10** | **2.7E-06** | 1 | **Yes** |
|  | 4 | 0.012 (2.8E-03-0.051) | 11 (2.6-43) | 67 (5-907) | **1.1E-10** | **8.5E-10** | **2.8E-07** | 0.31 | **9.8E-09** | **1.6E-07** | **2.9E-05** | 1 | No |
|  | 5 | 0.45 (0.083-2.5) | 91 (31-267) | 316 (43-2.3E+03) | **1.5E-07** | **8.4E-07** | **3.1E-05** | 0.23 | **7.3E-06** | **7.8E-05** | **2.0E-03** | 1 | No |
| IgG4:IgE (μg/L:μg/L) | 6m | 2.6E-03 (2.1E-03-3.2E-03) | 2.1E-03 (1.7E-03-2.6E-03) | 1.6E-03 (1.9E-04-0.013) | **7.1E-03** | 0.11 | **3.8E-03** | **0.021** | 0.15 | 1 | 0.14 | 0.6 | No* |
|  | 1 | 4.0E-03 (2.5E-03-6.2E-03) | 0.046 (0.017-0.13) | 0.41 (0.027-6.2) | **9.9E-04** | **6.5E-03** | **2.2E-04** | 0.15 | **0.025** | 0.22 | **0.011** | 1 | No* |
|  | 2 | 0.05 (0.015-0.17) | 75 (21-268) | 14 (1.2-160) | **1.0E-10** | **6.7E-11** | **3.7E-03** | **3.8E-03** | **9.1E-09** | **1.9E-08** | 0.14 | 0.14 | No* |
|  | 3 | 0.11 (0.028-0.39) | 626 (204-1.9E+03) | 75 (5.7-991) | **4.6E-14** | **1.3E-14** | **3.7E-04** | **9.5E-03** | **8.1E-12** | **1.2E-11** | **0.018** | 0.31 | No* |
|  | 4 | 0.26 (0.061-1.1) | 199 (48-818) | 76 (7-824) | **1.3E-08** | **5.8E-09** | **3.0E-03** | **0.028** | **7.6E-07** | **9.0E-07** | 0.12 | 0.75 | No |
|  | 5 | 7 (1.3-38) | 1.1E+03 (389-3.2E+03) | 179 (26-1.2E+03) | **2.3E-05** | **1.2E-05** | 0.28 | **0.011** | **7.8E-04** | **8.3E-04** | 1 | 0.34 | No |
| *Couch grass antibody* |  |  |  |  |  |  |  |  |  |  |  |  |  |
| IgE (kU/L) | 6m | 0.02 (0.017-0.024) | 0.021 (0.017-0.024) | 0.024 (0.016-0.035) | 0.47 | 0.88 | 0.28 | 0.24 | 1 | 1 | 1 | 1 | **Yes** |
|  | 1 | 0.019 (0.016-0.022) | 0.018 (0.016-0.02) | 0.029 (0.017-0.049) | **0.049** | 0.72 | **0.04** | **0.019** | 0.75 | 1 | 1 | 0.55 | **Yes** |
|  | 2 | 0.022 (0.019-0.026) | 0.019 (0.017-0.022) | 0.087 (0.05-0.15) | **7.2E-10** | *0.096* | **3.4E-07** | **2.7E-10** | **5.4E-08** | 1 | **3.4E-05** | **5.9E-08** | **Yes** |
|  | 3 | 0.019 (0.015-0.023) | 0.018 (0.015-0.021) | 0.12 (0.047-0.28) | **1.1E-12** | 0.23 | **5.7E-08** | **1.8E-11** | **1.5E-10** | 1 | **7.0E-06** | **5.8E-09** | **Yes** |
|  | 4 | 0.02 (0.015-0.025) | 0.023 (0.017-0.031) | 0.32 (0.12-0.89) | **5.8E-14** | 0.54 | **2.3E-11** | **2.0E-10** | **9.6E-12** | 1 | **7.1E-09** | **4.6E-08** | No |
|  | 5 | 0.029 (0.02-0.041) | 0.046 (0.029-0.072) | 1.2 (0.34-4.3) | **2.7E-08** | 0.17 | **1.5E-08** | **1.3E-06** | **1.5E-06** | 1 | **2.1E-06** | **1.2E-04** | No |
|  | 10 | 0.19 (0.095-0.38) | 0.12 (0.065-0.22) | 2.8 (0.86-9.4) | **3.3E-04** | 0.36 | **8.2E-04** | **7.6E-05** | **8.9E-03** | 1 | **0.036** | **4.5E-03** | No |
| IgG4 (μg/L) | 6m | 1.5E-04 (1.5E-04-1.5E-04) | 1.7E-04 (1.3E-04-2.1E-04) | 2.8E-04 (7.6E-05-1.0E-03) | 0.14 | 0.37 | *0.051* | 0.22 | 1 | 1 | 1 | 1 | **Yes** |
|  | 1 | 1.7E-04 (1.3E-04-2.3E-04) | 8.0E-04 (3.6E-04-1.8E-03) | 0.01 (6.2E-04-0.18) | **2.1E-05** | **1.3E-03** | **8.6E-07** | **0.015** | **7.2E-04** | *0.055* | **8.0E-05** | 0.45 | **Yes** |
|  | 2 | 2.0E-04 (1.3E-04-3.0E-04) | 0.02 (5.8E-03-0.067) | 0.14 (6.1E-03-3.2) | **7.3E-10** | **2.2E-09** | **5.3E-10** | 0.12 | **5.4E-08** | **3.8E-07** | **1.1E-07** | 1 | **Yes** |
|  | 3 | 2.6E-03 (8.2E-04-8.4E-03) | 13 (4.4-41) | 18 (1.4-241) | **3.4E-17** | **6.6E-17** | **3.3E-09** | 0.35 | **1.7E-14** | **1.1E-13** | **5.4E-07** | 1 | **Yes** |
|  | 4 | 0.037 (8.1E-03-0.17) | 42 (14-125) | 86 (11-698) | **6.4E-14** | **2.7E-13** | **1.8E-07** | 0.32 | **1.0E-11** | **1.9E-10** | **1.9E-05** | 1 | No |
|  | 5 | 0.26 (0.055-1.3) | 125 (59-265) | 196 (32-1.2E+03) | **9.1E-14** | **7.2E-13** | **3.0E-07** | 0.24 | **1.4E-11** | **4.2E-10** | **3.1E-05** | 1 | No |
| IgG4:IgE (μg/L:μg/L) | 6m | 3.1E-03 (2.6E-03-3.6E-03) | 3.4E-03 (2.6E-03-4.5E-03) | 4.9E-03 (1.2E-03-0.02) | 0.7 | 0.77 | 0.51 | 0.43 | 1 | 1 | 1 | 1 | No* |
|  | 1 | 3.8E-03 (2.8E-03-5.2E-03) | 0.018 (8.1E-03-0.042) | 0.15 (0.01-2.2) | **0.014** | **0.032** | **7.1E-03** | 0.17 | 0.26 | 0.84 | 0.24 | 1 | No* |
|  | 2 | 3.7E-03 (2.4E-03-5.8E-03) | 0.43 (0.12-1.5) | 0.66 (0.031-14) | **5.7E-07** | **2.9E-08** | **0.038** | 0.39 | **2.5E-05** | **3.8E-06** | 0.97 | 1 | No* |
|  | 3 | 0.058 (0.018-0.19) | 316 (104-962) | 65 (3.5-1.2E+03) | **3.7E-16** | **5.6E-17** | **1.1E-05** | 0.23 | **1.1E-13** | **1.1E-13** | **7.8E-04** | 1 | No* |
|  | 4 | 0.77 (0.18-3.4) | 759 (247-2.3E+03) | 111 (11-1.2E+03) | **1.7E-11** | **6.0E-12** | **0.014** | **3.4E-03** | **1.8E-09** | **2.3E-09** | 0.42 | 0.13 | No |
|  | 5 | 3.9 (0.74-20) | 1.1E+03 (497-2.6E+03) | 67 (9.6-472) | **1.1E-07** | **1.2E-07** | 0.47 | **5.7E-04** | **5.5E-06** | **1.4E-05** | 1 | **0.026** | No |
| *Ryegrass antibody* |  |  |  |  |  |  |  |  |  |  |  |  |  |
| IgE (kU/L) | 6m | 0.021 (0.018-0.025) | 0.022 (0.018-0.026) | 0.024 (0.016-0.035) | 0.54 | 0.74 | 0.3 | 0.34 | 1 | 1 | 1 | 1 | **Yes** |
|  | 1 | 0.017 (0.015-0.02) | 0.017 (0.016-0.019) | 0.025 (0.015-0.042) | *0.066* | 0.78 | **0.033** | **0.042** | 0.97 | 1 | 0.86 | 1 | **Yes** |
|  | 3 | 0.026 (0.02-0.035) | 0.02 (0.017-0.025) | 0.28 (0.1-0.78) | **2.0E-10** | 0.14 | **2.9E-07** | **1.2E-10** | **1.7E-08** | 1 | **3.0E-05** | **3.1E-08** | **Yes** |
|  | 4 | 0.024 (0.018-0.033) | 0.03 (0.022-0.042) | 0.86 (0.3-2.4) | **1.7E-11** | 0.27 | **1.1E-10** | **1.7E-09** | **1.8E-09** | 1 | **3.0E-08** | **3.0E-07** | No |
|  | 5 | 0.039 (0.026-0.059) | 0.056 (0.035-0.09) | 1.8 (0.49-6.4) | **2.5E-07** | 0.37 | **1.7E-07** | **1.4E-06** | **1.2E-05** | 1 | **1.9E-05** | **1.2E-04** | No |
|  | 10 | 0.3 (0.14-0.63) | 0.23 (0.12-0.45) | 4.3 (1.2-15) | **1.1E-03** | 0.77 | **1.2E-03** | **2.7E-04** | **0.027** | 1 | *0.051* | **0.014** | No |
| IgG4 (μg/L) | 6m | 1.5E-04 (1.5E-04-1.5E-04) | 1.5E-04 (1.5E-04-1.5E-04) | 2.6E-04 (8.1E-05-8.6E-04) | **0.013** | NA | *0.051* | **0.031** | 0.25 | NA | 1 | 0.81 | **Yes** |
|  | 1 | 1.8E-04 (1.3E-04-2.5E-04) | 2.6E-04 (1.6E-04-4.3E-04) | 5.9E-03 (3.8E-04-0.09) | **8.4E-06** | 0.17 | **1.2E-05** | **3.9E-04** | **3.0E-04** | 1 | **8.3E-04** | **0.019** | **Yes** |
|  | 2 | 2.0E-04 (1.3E-04-2.9E-04) | 5.9E-03 (1.8E-03-0.019) | 0.12 (5.5E-03-2.6) | **4.0E-08** | **1.6E-06** | **6.3E-10** | **0.041** | **2.2E-06** | **1.4E-04** | **1.2E-07** | 1 | **Yes** |
|  | 3 | 1.5E-04 (1.5E-04-1.5E-04) | 0.013 (3.7E-03-0.042) | 0.2 (9.7E-03-4.3) | **2.0E-10** | **3.7E-09** | **1.3E-12** | *0.056* | **1.7E-08** | **6.0E-07** | **6.5E-10** | 1 | **Yes** |
|  | 4 | 2.7E-04 (1.5E-04-5.0E-04) | 7.6E-03 (2.2E-03-0.027) | 0.19 (7.8E-03-4.8) | **2.7E-07** | **1.7E-05** | **2.9E-08** | **0.023** | **1.2E-05** | **1.1E-03** | **3.8E-06** | 0.64 | No |
|  | 5 | 3.8E-03 (9.7E-04-0.015) | 0.25 (0.056-1.1) | 30 (3-303) | **1.5E-07** | **8.5E-05** | **1.2E-07** | **5.1E-03** | **7.3E-06** | **4.9E-03** | **1.4E-05** | 0.18 | No |
| IgG4:IgE (μg/L:μg/L) | 6m | 3.0E-03 (2.5E-03-3.6E-03) | 2.9E-03 (2.4E-03-3.5E-03) | 4.7E-03 (1.3E-03-0.017) | 0.79 | 0.74 | 0.54 | 0.59 | 1 | 1 | 1 | 1 | No* |
|  | 1 | 4.2E-03 (3.0E-03-6.1E-03) | 6.4E-03 (3.9E-03-0.011) | 0.097 (7.1E-03-1.3) | **0.023** | 0.5 | **9.1E-03** | **0.027** | 0.4 | 1 | 0.29 | 0.73 | No* |
|  | 3 | 2.4E-03 (1.8E-03-3.1E-03) | 0.26 (0.075-0.89) | 0.3 (0.013-6.9) | **2.7E-06** | **1.7E-07** | **0.011** | 0.53 | **1.1E-04** | **1.9E-05** | 0.34 | 1 | No* |
|  | 4 | 4.7E-03 (2.5E-03-8.7E-03) | 0.1 (0.03-0.36) | 0.095 (3.9E-03-2.3) | **0.014** | **2.1E-03** | 0.44 | 0.32 | 0.26 | *0.084* | 1 | 1 | No |
|  | 5 | 0.041 (0.011-0.15) | 1.9 (0.43-8.3) | 7.1 (0.83-60) | **2.6E-04** | **5.5E-04** | **5.1E-04** | 0.75 | **7.2E-03** | **0.025** | **0.024** | 1 | No |
| *(Timothy) Grass antibody* |  |  |  |  |  |  |  |  |  |  |  |  |  |
| IgG (mg/L) | 1 | 0.24 (0.21-0.28) | 0.29 (0.25-0.34) | 0.35 (0.24-0.52) | **0.029** | **0.054** | **9.5E-03** | 0.22 | 0.48 | 1 | 0.31 | 1 | **Yes** |
|  | 2 | 0.37 (0.31-0.46) | 0.64 (0.52-0.79) | 0.51 (0.34-0.77) | **1.4E-03** | **3.0E-04** | 0.13 | 0.44 | **0.034** | **0.015** | 1 | 1 | **Yes** |
|  | 3 | 0.25 (0.23-0.28) | 0.38 (0.32-0.47) | 0.57 (0.37-0.9) | **9.8E-05** | **2.3E-03** | **2.3E-05** | *0.059* | **3.1E-03** | *0.091* | **1.5E-03** | 1 | **Yes** |
|  | 5 | 0.77 (0.61-0.98) | 0.77 (0.6-0.99) | 1.8 (1.1-2.9) | **0.014** | 0.88 | **4.7E-03** | **6.9E-03** | 0.26 | 1 | 0.17 | 0.23 | No |
|  | 10 | 1.4 (1.1-1.6) | 1.6 (1.3-1.9) | 1.5 (1.1-2) | 0.34 | 0.15 | 0.68 | 0.49 | 1 | 1 | 1 | 1 | No |
| *Mould antibody* |  |  |  |  |  |  |  |  |  |  |  |  |  |
| IgE (kU/L) | 6m | 0.018 (0.016-0.02) | 0.017 (0.015-0.02) | 0.019 (0.013-0.027) | 0.66 | 0.39 | 0.99 | 0.58 | 1 | 1 | 1 | 1 | **Yes** |
|  | 1 | 0.016 (0.015-0.017) | 0.016 (0.015-0.017) | 0.015 (0.015-0.015) | 0.77 | 0.82 | 0.48 | 0.53 | 1 | 1 | 1 | 1 | **Yes** |
|  | 2 | 0.018 (0.016-0.02) | 0.018 (0.016-0.021) | 0.025 (0.018-0.034) | **3.4E-03** | 0.94 | **3.6E-03** | **2.5E-03** | *0.075* | 1 | 0.13 | *0.098* | **Yes** |
|  | 3 | 0.022 (0.017-0.028) | 0.019 (0.016-0.023) | 0.048 (0.026-0.09) | **2.3E-06** | 0.49 | **1.3E-04** | **1.2E-06** | **9.1E-05** | 1 | **7.1E-03** | **1.1E-04** | **Yes** |
|  | 4 | 0.017 (0.015-0.02) | 0.026 (0.018-0.037) | 0.042 (0.023-0.078) | **1.4E-05** | 0.1 | **2.4E-06** | **1.6E-03** | **4.9E-04** | 1 | **2.0E-04** | *0.066* | No |
|  | 5 | 0.018 (0.015-0.022) | 0.029 (0.019-0.044) | 0.085 (0.034-0.21) | **7.8E-07** | *0.071* | **1.6E-07** | **2.8E-04** | **3.4E-05** | 1 | **1.8E-05** | **0.014** | No |
|  | 10 | 0.057 (0.033-0.096) | 0.087 (0.046-0.16) | 1 (0.34-3.2) | **1.2E-04** | 0.41 | **2.1E-05** | **3.8E-04** | **3.7E-03** | 1 | **1.4E-03** | **0.018** | No |
| IgG4 (μg/L) | 6m | 2.3E-04 (1.4E-04-3.6E-04) | 1.8E-04 (1.4E-04-2.5E-04) | 2.8E-04 (7.7E-05-1.0E-03) | 0.69 | 0.49 | 0.8 | 0.46 | 1 | 1 | 1 | 1 | **Yes** |
|  | 1 | 1.5E-04 (1.5E-04-1.5E-04) | 1.5E-04 (1.5E-04-1.5E-04) | 2.7E-04 (8.1E-05-8.8E-04) | **0.012** | NA | *0.051* | **0.029** | 0.23 | NA | 1 | 0.77 | **Yes** |
|  | 2 | 1.5E-04 (1.5E-04-1.5E-04) | 4.9E-04 (2.4E-04-9.9E-04) | 2.4E-04 (8.8E-05-6.8E-04) | **0.011** | **3.1E-03** | **0.049** | 0.41 | 0.22 | 0.12 | 1 | 1 | **Yes** |
|  | 3 | 2.0E-04 (1.3E-04-3.1E-04) | 1.1E-03 (4.6E-04-2.8E-03) | 2.1E-03 (2.2E-04-0.019) | **2.9E-03** | **1.6E-03** | **8.6E-04** | 0.6 | *0.065* | *0.066* | **0.037** | 1 | **Yes** |
|  | 4 | 5.2E-04 (2.3E-04-1.2E-03) | 1.8E-03 (6.3E-04-5.1E-03) | 0.097 (5.4E-03-1.7) | **7.6E-05** | *0.076* | **1.2E-05** | **2.5E-03** | **2.4E-03** | 1 | **8.3E-04** | *0.098* | No |
|  | 5 | 2.9E-03 (8.2E-04-0.01) | 0.3 (0.075-1.2) | 3.3 (0.21-51) | **9.3E-07** | **8.4E-06** | **5.6E-06** | 0.14 | **4.0E-05** | **6.1E-04** | **4.3E-04** | 1 | No |
| IgG4:IgE (μg/L:μg/L) | 6m | 5.2E-03 (3.2E-03-8.5E-03) | 4.4E-03 (3.2E-03-6.1E-03) | 6.2E-03 (1.6E-03-0.024) | 0.93 | 0.7 | 0.89 | 0.93 | 1 | 1 | 1 | 1 | No* |
|  | 1 | 3.9E-03 (3.6E-03-4.3E-03) | 4.0E-03 (3.8E-03-4.2E-03) | 7.4E-03 (2.2E-03-0.024) | 0.15 | 0.82 | *0.089* | *0.076* | 1 | 1 | 1 | 1 | No* |
|  | 2 | 3.5E-03 (3.1E-03-3.9E-03) | 0.011 (5.5E-03-0.023) | 4.1E-03 (1.3E-03-0.013) | **3.1E-03** | *0.065* | **0.018** | **3.8E-03** | *0.069* | 1 | 0.52 | 0.14 | No* |
|  | 3 | 3.9E-03 (2.3E-03-6.5E-03) | 0.025 (0.01-0.063) | 0.018 (1.7E-03-0.19) | **7.0E-03** | **4.5E-03** | 0.36 | **0.03** | 0.15 | 0.16 | 1 | 0.79 | No* |
|  | 4 | 0.013 (5.5E-03-0.03) | 0.029 (0.011-0.079) | 0.96 (0.046-20) | **0.041** | 0.23 | **0.017** | *0.072* | 0.65 | 1 | 0.5 | 1 | No |
|  | 5 | 0.054 (0.015-0.2) | 4.3 (1.1-16) | 16 (1.2-205) | **1.6E-05** | **1.0E-05** | **7.0E-04** | 0.78 | **5.6E-04** | **7.2E-04** | **0.031** | 1 | No |
| *Food mix antibody* |  |  |  |  |  |  |  |  |  |  |  |  |  |
| IgE (kU/L) | 5 | 0.039 (0.028-0.054) | 0.081 (0.055-0.12) | 2.1 (0.66-6.9) | **1.9E-09** | **6.9E-03** | **3.3E-09** | **3.6E-07** | **1.3E-07** | 0.23 | **5.4E-07** | **3.6E-05** | No |
| IgG4 (μg/L) | 5 | 758 (213-2.7E+03) | 2.2E+04 (1.6E+04-2.8E+04) | 1.1E+04 (1.2E+03-1.1E+05) | **4.7E-14** | **2.3E-13** | **6.8E-07** | 0.5 | **8.1E-12** | **1.7E-10** | **6.4E-05** | 1 | No |
| *Phadiatop Infant antibody* |  |  |  |  |  |  |  |  |  |  |  |  |  |
| IgE (PAU/L) | 6m | 0.033 (0.025-0.044) | 0.073 (0.053-0.1) | 1 (0.36-2.9) | **2.3E-10** | **2.8E-04** | **3.5E-10** | **2.4E-06** | **1.8E-08** | **0.014** | **7.6E-08** | **2.0E-04** | **Yes** |
|  | 1 | 0.12 (0.092-0.15) | 0.23 (0.18-0.3) | 5.7 (3.2-10) | **1.3E-14** | **1.7E-04** | **2.3E-12** | **1.8E-11** | **2.8E-12** | **9.2E-03** | **1.0E-09** | **5.8E-09** | **Yes** |
|  | 2 | 0.088 (0.069-0.11) | 0.29 (0.22-0.37) | 15 (9.7-23) | **1.6E-19** | **2.4E-09** | **4.7E-13** | **6.3E-13** | **1.3E-16** | **4.1E-07** | **3.0E-10** | **3.9E-10** | **Yes** |
|  | 3 | 0.094 (0.066-0.13) | 0.3 (0.21-0.43) | 28 (16-49) | **2.0E-16** | **5.7E-06** | **1.1E-12** | **2.2E-12** | **6.9E-14** | **4.3E-04** | **6.0E-10** | **9.7E-10** | **Yes** |
|  | 4 | 0.088 (0.057-0.14) | 0.46 (0.29-0.73) | 43 (22-82) | **4.9E-16** | **1.8E-07** | **8.7E-12** | **1.9E-10** | **1.3E-13** | **1.9E-05** | **3.1E-09** | **4.5E-08** | No |
|  | 5# | 0.085 (0.047-0.15) | 0.35 (0.18-0.66) | 35 (12-96) | **2.1E-10** | **1.8E-03** | **9.0E-10** | **1.1E-07** | **1.7E-08** | *0.074* | **1.6E-07** | **1.3E-05** | No |
|  | 10# | 1.1 (0.5-2.6) | 2.3 (1-5.4) | 67 (36-125) | **6.9E-06** | 0.15 | **2.0E-06** | **4.7E-05** | **2.5E-04** | 1 | **1.7E-04** | **2.9E-03** | No |
| IgG4 (PAU/L) | 6m | 9.0E-03 (2.1E-03-0.039) | 0.71 (0.16-3.2) | 0.67 (0.019-23) | **5.4E-04** | **1.7E-04** | **0.014** | 0.99 | **0.014** | **9.2E-03** | 0.42 | 1 | **Yes** |
|  | 1 | 66 (21-203) | 1.3E+03 (706-2.4E+03) | 1.4E+03 (240-8.7E+03) | **1.7E-09** | **1.9E-09** | **8.2E-05** | 0.58 | **1.2E-07** | **3.3E-07** | **4.7E-03** | 1 | **Yes** |
|  | 2 | 64 (15-273) | 1.0E+04 (5.7E+03-1.8E+04) | 5.7E+03 (904-3.5E+04) | **1.9E-16** | **1.2E-16** | **5.1E-06** | 0.69 | **6.9E-14** | **1.8E-13** | **3.9E-04** | 1 | **Yes** |
|  | 3 | 677 (232-2.0E+03) | 2.3E+04 (1.9E+04-2.9E+04) | 2.5E+04 (1.4E+04-4.7E+04) | **1.8E-20** | **3.9E-20** | **4.0E-08** | 0.96 | **2.4E-17** | **1.9E-16** | **5.0E-06** | 1 | **Yes** |
|  | 4 | 575 (172-1.9E+03) | 2.2E+04 (1.6E+04-2.8E+04) | 2.4E+04 (1.3E+04-4.6E+04) | **2.4E-16** | **5.2E-16** | **4.7E-07** | 0.81 | **7.8E-14** | **6.2E-13** | **4.6E-05** | 1 | No |
|  | 5# | 0.016 (3.0E-03-0.081) | 59 (17-206) | 643 (92-4.5E+03) | **5.9E-14** | **3.7E-11** | **5.9E-10** | **3.6E-03** | **9.6E-12** | **1.1E-08** | **1.2E-07** | 0.13 | No |
| IgG4:IgE (μg/L:μg/L) | 6m | 0.27 (0.063-1.2) | 9.7 (2.1-44) | 0.66 (0.023-19) | **0.02** | **0.031** | 0.42 | **0.02** | 0.36 | 0.81 | 1 | 0.57 | No* |
|  | 1 | 561 (179-1.8E+03) | 5.6E+03 (3.0E+03-1.1E+04) | 255 (44-1.5E+03) | **4.3E-07** | **1.9E-04** | **0.012** | **2.0E-06** | **2.0E-05** | **0.01** | 0.37 | **1.7E-04** | No* |
|  | 2 | 722 (170-3.1E+03) | 3.5E+04 (1.9E+04-6.3E+04) | 380 (57-2.5E+03) | **1.8E-11** | **1.5E-06** | **2.0E-03** | **2.6E-10** | **1.8E-09** | **1.3E-04** | *0.08* | **5.8E-08** | No* |
|  | 3 | 7.2E+03 (2.5E+03-2.1E+04) | 7.6E+04 (5.0E+04-1.2E+05) | 898 (347-2.3E+03) | **1.7E-11** | **1.2E-05** | **1.7E-05** | **5.4E-10** | **1.8E-09** | **8.3E-04** | **1.1E-03** | **1.1E-07** | No* |
|  | 4 | 6.5E+03 (2.0E+03-2.1E+04) | 4.7E+04 (2.8E+04-7.8E+04) | 564 (196-1.6E+03) | **5.5E-08** | **0.016** | **4.2E-05** | **9.3E-09** | **2.9E-06** | 0.47 | **2.6E-03** | **1.3E-06** | No |
|  | 5# | 0.18 (0.036-0.88) | 170 (47-612) | 19 (1.9-178) | **1.8E-07** | **2.0E-07** | **6.4E-03** | **7.0E-03** | **8.6E-06** | **2.1E-05** | 0.22 | 0.24 | No |
|  |  | **Prop. (95% CI)** | **Prop. (95% CI)** | **Prop. (95% CI)** | **Overall** | **1 vs. 2** | **1 vs. 3** | **2 vs. 3** | **Overall** | **1 vs. 2** | **1 vs. 3** | **2 vs. 3** |  |
| *Total antibody past*  *atopy threshold* |  |  |  |  |  |  |  |  |  |  |  |  |  |
| IgE ≥ 100 kU/L | 6m | 0% (0%-0%) | 1.9% (0%-4.6%) | 9.1% (0%-22%) | **0.029** | 0.5 | **0.04** | 0.14 | 0.48 | 1 | 1 | 1 | No* |
|  | 1 | 0% (0%-0%) | 2.8% (0%-6%) | 36% (15%-58%) | **1.2E-07** | 0.26 | **9.1E-07** | **2.5E-05** | **5.9E-06** | 1 | **8.4E-05** | **1.6E-03** | No* |
|  | 2 | 6.9% (1.5%-12%) | 9.6% (3.9%-15%) | 73% (53%-93%) | **1.2E-10** | 0.6 | **6.3E-10** | **3.2E-09** | **1.1E-08** | 1 | **1.2E-07** | **5.3E-07** | No* |
|  | 3 | 6% (0.8%-11%) | 15% (7.8%-22%) | 86% (71%-100%) | **7.5E-14** | *0.061* | **1.5E-13** | **1.9E-10** | **1.2E-11** | 1 | **1.2E-10** | **4.5E-08** | No* |
|  | 4 | 12% (4.3%-19%) | 26% (17%-35%) | 86% (69%-100%) | **4.7E-10** | **0.019** | **1.8E-10** | **7.0E-07** | **3.6E-08** | 0.55 | **4.4E-08** | **6.6E-05** | No |
|  | 5 | 27% (16%-38%) | 35% (25%-46%) | 94% (83%-100%) | **4.8E-07** | 0.29 | **1.9E-07** | **6.1E-06** | **2.2E-05** | 1 | **2.0E-05** | **4.6E-04** | No |
|  | 10 | 48% (34%-62%) | 60% (47%-73%) | 100% (100%-100%) | **6.6E-04** | 0.25 | **3.7E-04** | **3.0E-03** | **0.017** | 1 | **0.018** | 0.12 | No |
| *HDM antibody past*  *atopy threshold* |  |  |  |  |  |  |  |  |  |  |  |  |  |
| IgE ≥ 0.35 kU/L | 6m | 0% (0%-0%) | 1.9% (0%-4.6%) | 14% (0%-29%) | **4.5E-03** | 0.5 | **7.5E-03** | **0.037** | *0.097* | 1 | 0.25 | 0.94 | No* |
|  | 1 | 0% (0%-0%) | 1.9% (0%-4.5%) | 50% (27%-73%) | **2.9E-11** | 0.5 | **2.0E-09** | **1.7E-08** | **2.8E-09** | 1 | **3.5E-07** | **2.3E-06** | No* |
|  | 2 | 2.3% (0%-5.5%) | 14% (7.6%-21%) | 86% (71%-100%) | **2.0E-16** | **3.9E-03** | **3.8E-16** | **1.2E-10** | **6.9E-14** | 0.14 | **4.8E-13** | **3.1E-08** | No* |
|  | 3 | 12% (4.9%-19%) | 19% (11%-27%) | 91% (78%-100%) | **2.1E-12** | 0.23 | **3.5E-12** | **2.1E-10** | **2.7E-10** | 1 | **1.4E-09** | **4.8E-08** | No* |
|  | 4 | 12% (4.3%-19%) | 34% (24%-44%) | 90% (77%-100%) | **2.0E-11** | **8.3E-04** | **1.1E-11** | **2.8E-06** | **2.0E-09** | **0.036** | **3.9E-09** | **2.3E-04** | No |
|  | 5 | 23% (13%-33%) | 39% (28%-50%) | 89% (73%-100%) | **1.1E-06** | **0.035** | **4.0E-07** | **1.5E-04** | **4.6E-05** | 0.9 | **4.0E-05** | **8.2E-03** | No |
|  | 10 | 49% (35%-63%) | 58% (45%-72%) | 100% (100%-100%) | **8.4E-04** | 0.44 | **3.7E-04** | **2.9E-03** | **0.021** | 1 | **0.018** | 0.11 | No |
| *Cat antibody past*  *atopy threshold* |  |  |  |  |  |  |  |  |  |  |  |  |  |
| IgE ≥ 0.35 kU/L | 6m | 1.2% (0%-3.5%) | 1.9% (0%-4.6%) | 9.1% (0%-22%) | 0.17 | 1 | 0.1 | 0.14 | 1 | 1 | 1 | 1 | No* |
|  | 1 | 0% (0%-0%) | 0% (0%-0%) | 14% (0%-29%) | **9.4E-04** | 1 | **7.5E-03** | **4.4E-03** | **0.024** | 1 | 0.25 | 0.16 | No* |
|  | 2 | 0% (0%-0%) | 2.9% (0%-6.2%) | 23% (3.7%-42%) | **9.8E-05** | 0.25 | **2.3E-04** | **4.2E-03** | **3.1E-03** | 1 | **0.012** | 0.15 | No* |
|  | 3 | 3.6% (0%-7.7%) | 2% (0%-4.7%) | 23% (3.7%-42%) | **2.3E-03** | 0.66 | **9.6E-03** | **2.0E-03** | *0.053* | 1 | 0.31 | *0.08* | No* |
|  | 4 | 0% (0%-0%) | 5.7% (0.75%-11%) | 24% (3.9%-44%) | **2.6E-04** | *0.061* | **2.8E-04** | **0.022** | **7.2E-03** | 1 | **0.014** | 0.62 | No |
|  | 5 | 0% (0%-0%) | 8.9% (2.5%-15%) | 39% (14%-64%) | **3.3E-06** | **0.015** | **5.0E-06** | **3.8E-03** | **1.3E-04** | 0.45 | **3.9E-04** | 0.14 | No |
|  | 10 | 17% (6.7%-28%) | 20% (9.1%-31%) | 71% (44%-98%) | **2.9E-04** | 0.81 | **2.3E-04** | **4.9E-04** | **8.0E-03** | 1 | **0.012** | **0.023** | No |
| *Peanut antibody past*  *atopy threshold* |  |  |  |  |  |  |  |  |  |  |  |  |  |
| IgE ≥ 0.35 kU/L | 6m | 2.3% (0%-5.6%) | 4.8% (0.63%-9%) | 41% (19%-63%) | **1.5E-06** | 0.46 | **5.4E-06** | **3.5E-05** | **6.3E-05** | 1 | **4.2E-04** | **2.2E-03** | No* |
|  | 1 | 3.5% (0%-7.4%) | 5.6% (1.2%-10%) | 68% (47%-89%) | **3.7E-12** | 0.73 | **1.3E-10** | **4.3E-10** | **4.6E-10** | 1 | **3.3E-08** | **9.1E-08** | No* |
|  | 2 | 1.1% (0%-3.4%) | 2.9% (0%-6.2%) | 64% (42%-85%) | **1.8E-13** | 0.63 | **2.8E-11** | **1.3E-10** | **2.6E-11** | 1 | **8.3E-09** | **3.3E-08** | No* |
|  | 3 | 4.8% (0.11%-9.5%) | 0.99% (0%-3%) | 45% (23%-68%) | **9.6E-09** | 0.18 | **1.4E-05** | **4.3E-08** | **5.7E-07** | 1 | **9.6E-04** | **5.4E-06** | No* |
|  | 4 | 1.3% (0%-3.8%) | 3.4% (0%-7.3%) | 52% (29%-76%) | **1.3E-09** | 0.62 | **3.0E-08** | **2.5E-07** | **9.1E-08** | 1 | **3.9E-06** | **2.6E-05** | No |
|  | 5 | 7.4% (0.99%-14%) | 13% (5.2%-20%) | 56% (30%-81%) | **3.7E-05** | 0.41 | **2.2E-05** | **2.6E-04** | **1.2E-03** | 1 | **1.4E-03** | **0.013** | No |
|  | 10 | 21% (9.7%-33%) | 18% (7.7%-29%) | 64% (36%-93%) | **2.7E-03** | 0.81 | **3.4E-03** | **1.4E-03** | *0.062* | 1 | 0.13 | *0.059* | No |
| *Couch grass antibody past*  *atopy threshold* |  |  |  |  |  |  |  |  |  |  |  |  |  |
| IgE ≥ 0.35 kU/L | 6m | 1.2% (0%-3.5%) | 0.96% (0%-2.9%) | 4.5% (0%-14%) | 0.46 | 1 | 0.37 | 0.32 | 1 | 1 | 1 | 1 | No* |
|  | 1 | 0% (0%-0%) | 0% (0%-0%) | 4.5% (0%-14%) | 0.1 | 1 | 0.2 | 0.17 | 1 | 1 | 1 | 1 | No* |
|  | 2 | 1.1% (0%-3.4%) | 0.96% (0%-2.9%) | 9.1% (0%-22%) | *0.054* | 1 | 0.1 | *0.079* | 0.82 | 1 | 1 | 1 | No* |
|  | 3 | 2.4% (0%-5.8%) | 2% (0%-4.7%) | 36% (15%-58%) | **2.3E-06** | 1 | **3.9E-05** | **1.1E-05** | **9.1E-05** | 1 | **2.4E-03** | **7.8E-04** | No* |
|  | 4 | 3.8% (0%-8.2%) | 6.8% (1.4%-12%) | 57% (34%-80%) | **2.0E-08** | 0.5 | **1.1E-07** | **1.0E-06** | **1.1E-06** | 1 | **1.3E-05** | **9.1E-05** | No |
|  | 5 | 8.8% (1.9%-16%) | 16% (8.1%-25%) | 72% (49%-95%) | **2.7E-07** | 0.22 | **1.8E-07** | **8.6E-06** | **1.2E-05** | 1 | **1.9E-05** | **6.2E-04** | No |
|  | 10 | 35% (21%-48%) | 29% (17%-41%) | 86% (65%-100%) | **3.6E-04** | 0.68 | **8.1E-04** | **1.7E-04** | **9.6E-03** | 1 | **0.036** | **9.2E-03** | No |
| *Ryegrass antibody past*  *atopy threshold* |  |  |  |  |  |  |  |  |  |  |  |  |  |
| IgE ≥ 0.35 kU/L | 6m | 1.2% (0%-3.5%) | 2.9% (0%-6.2%) | 4.5% (0%-14%) | 0.4 | 0.63 | 0.37 | 0.54 | 1 | 1 | 1 | 1 | No* |
|  | 1 | 0% (0%-0%) | 0% (0%-0%) | 4.5% (0%-14%) | 0.1 | 1 | 0.2 | 0.17 | 1 | 1 | 1 | 1 | No* |
|  | 3 | 6% (0.8%-11%) | 3% (0%-6.3%) | 50% (27%-73%) | **5.6E-08** | 0.47 | **6.9E-06** | **1.3E-07** | **2.9E-06** | 1 | **5.1E-04** | **1.5E-05** | No* |
|  | 4 | 5.1% (0.12%-10%) | 10% (3.8%-17%) | 71% (50%-92%) | **1.6E-10** | 0.26 | **7.4E-10** | **3.9E-08** | **1.4E-08** | 1 | **1.4E-07** | **5.0E-06** | No |
|  | 5 | 10% (2.8%-17%) | 22% (12%-31%) | 78% (57%-99%) | **9.5E-08** | *0.074* | **3.8E-08** | **1.2E-05** | **4.9E-06** | 1 | **4.9E-06** | **8.3E-04** | No |
|  | 10 | 40% (27%-54%) | 40% (27%-53%) | 86% (65%-100%) | **5.7E-03** | 1 | **5.3E-03** | **2.7E-03** | 0.12 | 1 | 0.19 | 0.11 | No |
| *Mould antibody past*  *atopy threshold* |  |  |  |  |  |  |  |  |  |  |  |  |  |
| IgE ≥ 0.35 kU/L | 6m | 0% (0%-0%) | 0.96% (0%-2.9%) | 4.5% (0%-14%) | 0.2 | 1 | 0.2 | 0.32 | 1 | 1 | 1 | 1 | No* |
|  | 1 | 0% (0%-0%) | 0% (0%-0%) | 0% (0%-0%) | 1 | 1 | 1 | 1 | 1 | 1 | 1 | 1 | No* |
|  | 2 | 1.1% (0%-3.4%) | 1.9% (0%-4.6%) | 0% (0%-0%) | 1 | 1 | 1 | 1 | 1 | 1 | 1 | 1 | No* |
|  | 3 | 7.2% (1.5%-13%) | 4% (0.091%-7.8%) | 14% (0%-29%) | 0.16 | 0.35 | 0.39 | 0.11 | 1 | 1 | 1 | 1 | No* |
|  | 4 | 1.3% (0%-3.8%) | 9.1% (3%-15%) | 9.5% (0%-23%) | **0.047** | **0.037** | 0.11 | 1 | 0.72 | 0.94 | 1 | 1 | No |
|  | 5 | 2.9% (0%-7.1%) | 10% (3.3%-17%) | 22% (0.95%-43%) | **0.027** | 0.11 | **0.016** | 0.23 | 0.46 | 1 | 0.47 | 1 | No |
|  | 10 | 15% (5.2%-26%) | 20% (9.1%-31%) | 79% (54%-100%) | **1.9E-05** | 0.62 | **1.7E-05** | **7.9E-05** | **6.5E-04** | 1 | **1.1E-03** | **4.6E-03** | No |

Feature? = whether variable was used as a clustering feature or not; geom. mean = geometric mean; HDM = house dust mite; P-value (adj.) = adjusted P-values (Benjamini-Yekutieli method). For categorical variables, associations were tested using Fisher exact test; for continuous variables, Kruskal-Wallis and Mann-Whitney-Wilcoxon. Bold text indicates statistical significance (*p*<0.05); italics indicate near-significance (*p*<0.10). *Not used as clustering features, as these were derived variables. #Assay used at age 5 was the adult version, not Phadiatop infant. Therefore the standard unit (PAU) and specificities may differ between the two.

1. **Immunological (cytokines)**

| **Variable** | **Age** | **CAS1 (N=88)** | **CAS2 (N=107)** | **CAS3 (N=22)** | **P-value** |  |  |  | **P-value (adj.)** |  |  |  | **Feature?** |
| --- | --- | --- | --- | --- | --- | --- | --- | --- | --- | --- | --- | --- | --- |
|  |  | **Geom. mean (95% CI)** | **Geom. mean (95% CI)** | **Geom. mean (95% CI)** | **Overall** | **1 vs. 2** | **1 vs. 3** | **2 vs. 3** | **Overall** | **1 vs. 2** | **1 vs. 3** | **2 vs. 3** |  |
| *HDM cytokine response*  *above control^* |  |  |  |  |  |  |  |  |  |  |  |  |  |
| IL-13 mRNA^ | 0 | 1.7E-03 (1.1E-04-0.026) | 6.0E-03 (4.8E-04-0.075) | 6.7E-03 (3.3E-05-1.4) | 0.85 | 0.6 | 0.68 | 0.94 | 1 | 1 | 1 | 1 | No |
|  | 6m | 1.0E-04 (8.8E-06-1.1E-03) | 3.2E-04 (3.8E-05-2.6E-03) | 2 (0.015-266) | **3.2E-04** | 0.5 | **1.7E-04** | **3.8E-04** | **8.7E-03** | 1 | **9.2E-03** | **0.018** | No |
|  | 1 | 2.2E-05 (2.9E-06-1.6E-04) | 4.9E-05 (5.4E-06-4.4E-04) | 5.8E-03 (3.2E-05-1.1) | **0.026** | 0.57 | **8.4E-03** | **0.037** | 0.45 | 1 | 0.27 | 0.94 | No |
|  | 2 | 1.9E-06 (7.7E-07-4.7E-06) | 1.7E-06 (8.2E-07-3.3E-06) | 2.2E-05 (6.4E-07-7.5E-04) | **0.04** | 0.83 | *0.054* | **0.023** | 0.64 | 1 | 1 | 0.64 | No |
|  | 3 | 1.5E-06 (6.7E-07-3.3E-06) | 1.4E-06 (7.2E-07-2.7E-06) | 1.0E-06 (1.0E-06-1.0E-06) | 0.87 | 0.9 | 0.62 | 0.65 | 1 | 1 | 1 | 1 | No |
|  | 5 | 0.036 (1.6E-03-0.8) | 0.11 (8.8E-03-1.4) | 2.9E+03 (742-1.1E+04) | **6.8E-05** | 0.59 | **9.9E-05** | **2.5E-05** | **2.2E-03** | 1 | **5.6E-03** | **1.6E-03** | No |
| IL-4 mRNA^ | 0 | 1.4E-06 (6.9E-07-3.0E-06) | 1.9E-06 (7.8E-07-4.4E-06) | 1.0E-06 (1.0E-06-1.0E-06) | 0.71 | 0.65 | 0.6 | 0.47 | 1 | 1 | 1 | 1 | No |
|  | 6m | 4.6E-06 (1.0E-06-2.1E-05) | 5.1E-06 (1.4E-06-1.8E-05) | 0.54 (6.5E-03-44) | **6.2E-09** | 0.94 | **4.7E-07** | **1.0E-07** | **3.8E-07** | 1 | **4.6E-05** | **1.2E-05** | No |
|  | 1 | 4.2E-06 (1.0E-06-1.7E-05) | 1.8E-05 (2.8E-06-1.2E-04) | 8.9E-04 (8.1E-06-0.099) | **9.3E-03** | 0.22 | **2.9E-03** | **0.035** | 0.19 | 1 | 0.11 | 0.9 | No |
|  | 2 | 1.2E-05 (2.3E-06-6.1E-05) | 7.9E-06 (2.0E-06-3.1E-05) | 0.01 (1.0E-04-1) | **1.3E-04** | 0.68 | **5.5E-04** | **1.0E-04** | **4.0E-03** | 1 | **0.025** | **5.6E-03** | No |
|  | 3 | 2.1E-06 (7.3E-07-6.2E-06) | 3.7E-06 (1.0E-06-1.4E-05) | 3.9E-06 (2.0E-07-7.7E-05) | 0.8 | 0.53 | 0.65 | 0.96 | 1 | 1 | 1 | 1 | No |
|  | 5 | 2.3E-04 (1.7E-05-3.0E-03) | 4.7E-04 (5.3E-05-4.3E-03) | 5.3 (0.082-345) | **4.9E-04** | 0.72 | **4.5E-04** | **3.2E-04** | **0.013** | 1 | **0.021** | **0.016** | No |
| IL-4R mRNA^ | 6m | 0.011 (6.1E-04-0.19) | 0.028 (3.0E-03-0.26) | 0.23 (1.1E-03-51) | 0.85 | 0.91 | 0.67 | 0.57 | 1 | 1 | 1 | 1 | No |
|  | 1 | 4.0E-03 (2.6E-04-0.062) | 9.5E-03 (6.7E-04-0.14) | 1.5E-03 (9.3E-06-0.25) | 0.85 | 0.79 | 0.7 | 0.59 | 1 | 1 | 1 | 1 | No |
|  | 2 | 1.5E-05 (2.8E-06-8.1E-05) | 1.2E-05 (2.8E-06-5.3E-05) | 7.1E-06 (4.1E-07-1.2E-04) | 0.91 | 0.92 | 0.66 | 0.75 | 1 | 1 | 1 | 1 | No |
|  | 3 | 3.2E-03 (1.9E-04-0.055) | 0.012 (8.5E-04-0.17) | 2.4 (9.0E-03-642) | **0.023** | 0.54 | **7.6E-03** | **0.019** | 0.4 | 1 | 0.25 | 0.55 | No |
|  | 5 | 0.27 (0.013-5.5) | 1 (0.1-11) | 3.2 (5.7E-03-1.8E+03) | **0.095** | 0.75 | **0.035** | 0.05 | 1 | 1 | 0.9 | 1 | No |
| IL-5 mRNA^ | 0 | 2.5E-04 (2.1E-05-2.9E-03) | 2.6E-04 (2.8E-05-2.5E-03) | 1.2E-05 (3.1E-07-4.6E-04) | 0.47 | 0.96 | 0.24 | 0.25 | 1 | 1 | 1 | 1 | No |
|  | 6m | 5.2E-05 (5.6E-06-4.8E-04) | 3.1E-05 (5.2E-06-1.8E-04) | 0.33 (1.3E-03-83) | **1.5E-04** | 0.85 | **2.3E-04** | **1.1E-04** | **4.5E-03** | 1 | **0.012** | **6.1E-03** | No |
|  | 1 | 4.6E-06 (1.0E-06-2.0E-05) | 2.2E-05 (2.9E-06-1.7E-04) | 0.015 (1.0E-04-2.4) | **2.2E-04** | 0.2 | **7.2E-05** | **3.6E-03** | **6.3E-03** | 1 | **4.2E-03** | 0.13 | No |
|  | 2 | 4.6E-06 (1.2E-06-1.7E-05) | 3.7E-06 (1.2E-06-1.1E-05) | 2.0E-03 (1.6E-05-0.25) | **1.7E-04** | 0.8 | **7.5E-04** | **2.0E-04** | **5.0E-03** | 1 | **0.033** | **0.011** | No |
|  | 3 | 2.2E-06 (7.2E-07-7.0E-06) | 1.0E-06 (1.0E-06-1.0E-06) | 1.9E-05 (2.5E-07-1.4E-03) | **0.027** | 0.13 | 0.16 | **4.8E-03** | 0.46 | 1 | 1 | 0.17 | No |
|  | 5 | 0.021 (9.9E-04-0.43) | 0.07 (5.7E-03-0.85) | 246 (7-8.7E+03) | **1.3E-04** | 0.49 | **7.1E-05** | **1.1E-04** | **4.0E-03** | 1 | **4.2E-03** | **6.1E-03** | No |
| IL-9 mRNA^ | 0 | 0.021 (1.0E-03-0.43) | 6.0E-03 (4.2E-04-0.085) | 5.9E-04 (3.4E-06-0.1) | 0.5 | 0.67 | 0.28 | 0.33 | 1 | 1 | 1 | 1 | No |
|  | 6m | 7.0E-03 (3.8E-04-0.13) | 3.1E-03 (3.1E-04-0.032) | 2 (3.4E-03-1.1E+03) | **0.027** | 0.6 | **0.022** | **8.8E-03** | 0.46 | 1 | 0.62 | 0.29 | No |
|  | 1 | 8.1E-05 (7.7E-06-8.5E-04) | 4.9E-04 (3.5E-05-6.9E-03) | 3.6 (0.072-175) | **2.3E-04** | 0.31 | **6.9E-05** | **1.6E-03** | **6.5E-03** | 1 | **4.1E-03** | *0.066* | No |
|  | 2 | 2.0E-06 (7.6E-07-5.0E-06) | 2.4E-05 (4.5E-06-1.3E-04) | 1.3E-03 (7.6E-06-0.23) | **1.2E-03** | **0.016** | **2.1E-04** | **0.041** | **0.029** | 0.47 | **0.011** | 1 | No |
|  | 3 | 5.8E-06 (1.0E-06-3.2E-05) | 1.4E-05 (2.1E-06-9.3E-05) | 2.5E-03 (4.6E-06-1.4) | **0.02** | 0.5 | **7.8E-03** | **0.029** | 0.36 | 1 | 0.26 | 0.77 | No |
|  | 5 | 3.3E-03 (1.0E-04-0.11) | 9.6E-03 (5.4E-04-0.17) | 1.5E+03 (4.2-5.4E+05) | **3.5E-04** | 0.77 | **2.4E-04** | **2.5E-04** | **9.4E-03** | 1 | **0.012** | **0.013** | No |
| IFN-γ mRNA^ | 0 | 0.68 (0.031-15) | 2.5 (0.18-34) | 0.1 (2.4E-04-45) | 0.67 | 0.9 | 0.48 | 0.37 | 1 | 1 | 1 | 1 | No |
|  | 6m | 0.46 (0.024-9) | 0.15 (0.012-1.8) | 23 (0.074-6.9E+03) | **0.043** | 0.46 | **0.045** | **0.014** | 0.68 | 1 | 1 | 0.42 | No |
|  | 1 | 1.2E-03 (7.0E-05-0.02) | 6.9E-04 (4.3E-05-0.011) | 0.054 (1.8E-04-16) | 0.14 | 0.71 | *0.086* | *0.066* | 1 | 1 | 1 | 1 | No |
|  | 2 | 0.09 (8.3E-03-0.97) | 0.31 (0.04-2.4) | 0.068 (4.8E-04-9.6) | 0.46 | 0.24 | 0.43 | 0.88 | 1 | 1 | 1 | 1 | No |
|  | 3 | 3.6E-06 (8.4E-07-1.5E-05) | 5.7E-06 (1.3E-06-2.6E-05) | 1.8E-05 (2.6E-07-1.3E-03) | 0.63 | 0.66 | 0.33 | 0.55 | 1 | 1 | 1 | 1 | No |
|  | 5 | 0.013 (5.4E-04-0.31) | 0.056 (3.8E-03-0.84) | 8.5 (0.034-2.1E+03) | 0.1 | 0.33 | **0.028** | 0.15 | 1 | 1 | 0.75 | 1 | No |
| IL-13 protein (pg/ml)^ | 0 | 0.22 (0.066-0.73) | 0.22 (0.076-0.63) | 0.085 (0.011-0.66) | 0.68 | 0.76 | 0.41 | 0.45 | 1 | 1 | 1 | 1 | No |
|  | 6m | 0.064 (0.022-0.18) | 0.06 (0.025-0.14) | 19 (1.4-244) | **4.6E-06** | 0.98 | **1.7E-05** | **4.1E-06** | **1.7E-04** | 1 | **1.1E-03** | **3.3E-04** | No |
|  | 1 | 0.044 (0.016-0.12) | 0.031 (0.014-0.07) | 0.82 (0.071-9.5) | **2.8E-03** | 0.53 | **6.5E-03** | **1.5E-03** | *0.063* | 1 | 0.22 | *0.063* | No |
|  | 2 | 0.021 (0.011-0.04) | 0.024 (0.013-0.046) | 0.57 (0.058-5.6) | **1.8E-04** | 0.79 | **3.1E-04** | **4.5E-04** | **5.3E-03** | 1 | **0.015** | **0.021** | No |
|  | 3 | 0.03 (0.014-0.068) | 0.035 (0.017-0.074) | 0.37 (0.029-4.8) | **0.027** | 0.78 | **0.016** | **0.017** | 0.46 | 1 | 0.47 | 0.5 | No |
|  | 5 | 0.13 (0.046-0.37) | 0.32 (0.11-0.87) | 12 (1.2-117) | **2.1E-04** | 0.29 | **7.7E-05** | **5.1E-04** | **6.1E-03** | 1 | **4.5E-03** | **0.024** | No |
| IL-5 protein (pg/ml)^ | 0 | 0.043 (0.018-0.11) | 0.026 (0.013-0.052) | 0.018 (5.0E-03-0.068) | 0.44 | 0.36 | 0.29 | 0.57 | 1 | 1 | 1 | 1 | No |
|  | 6m | 0.018 (9.2E-03-0.034) | 0.013 (8.9E-03-0.02) | 0.21 (0.012-3.7) | **7.9E-04** | 0.4 | **8.1E-03** | **3.5E-04** | **0.02** | 1 | 0.27 | **0.017** | No |
|  | 1 | 0.012 (8.5E-03-0.016) | 0.012 (8.3E-03-0.017) | 0.017 (5.6E-03-0.049) | 0.58 | 0.92 | 0.41 | 0.36 | 1 | 1 | 1 | 1 | No |
|  | 2 | 0.011 (8.7E-03-0.015) | 0.021 (0.012-0.039) | 0.044 (8.0E-03-0.25) | *0.054* | *0.088* | **0.011** | 0.28 | 0.82 | 1 | 0.34 | 1 | No |
|  | 3 | 0.016 (9.4E-03-0.026) | 0.019 (0.011-0.033) | 0.21 (0.018-2.4) | **2.1E-03** | 0.65 | **1.9E-03** | **3.9E-03** | *0.05* | 1 | *0.077* | 0.14 | No |
|  | 5 | 0.028 (0.014-0.057) | 0.042 (0.02-0.087) | 2.3 (0.25-22) | **3.2E-06** | 0.45 | **5.7E-06** | **2.0E-05** | **1.2E-04** | 1 | **4.3E-04** | **1.3E-03** | No |
| IL-10 protein (pg/ml)^ | 0 | 0.089 (0.032-0.24) | 0.19 (0.066-0.53) | 0.35 (0.038-3.2) | 0.32 | 0.26 | 0.17 | 0.57 | 1 | 1 | 1 | 1 | No |
|  | 6m | 0.055 (0.02-0.15) | 0.026 (0.013-0.051) | 0.19 (0.012-3) | *0.09* | 0.19 | 0.28 | **0.032** | 1 | 1 | 1 | 0.84 | No |
|  | 1 | 0.02 (0.01-0.04) | 0.014 (8.8E-03-0.021) | 0.01 (0.01-0.01) | 0.33 | 0.3 | 0.24 | 0.46 | 1 | 1 | 1 | 1 | No |
|  | 2 | 0.011 (8.7E-03-0.015) | 0.011 (8.9E-03-0.014) | 0.01 (0.01-0.01) | 0.87 | 0.91 | 0.61 | 0.64 | 1 | 1 | 1 | 1 | No |
|  | 3 | 0.01 (0.01-0.01) | 0.011 (8.9E-03-0.014) | 0.01 (0.01-0.01) | 0.59 | 0.37 | NA | 0.66 | 1 | 1 | NA | 1 | No |
|  | 5 | 0.019 (0.011-0.032) | 0.015 (0.01-0.023) | 0.015 (6.2E-03-0.039) | 0.82 | 0.55 | 0.75 | 1 | 1 | 1 | 1 | 1 | No |
| IFN-γ protein (pg/ml)^ | 0 | 0.085 (0.028-0.26) | 0.081 (0.03-0.22) | 0.057 (7.8E-03-0.42) | 0.95 | 0.89 | 0.72 | 0.85 | 1 | 1 | 1 | 1 | No |
|  | 6m | 0.094 (0.028-0.32) | 0.075 (0.028-0.2) | 0.46 (0.012-17) | 0.43 | 0.82 | 0.28 | 0.21 | 1 | 1 | 1 | 1 | No |
|  | 1 | 0.038 (0.015-0.1) | 0.019 (0.01-0.035) | 0.055 (7.8E-03-0.38) | 0.3 | 0.2 | 0.73 | 0.17 | 1 | 1 | 1 | 1 | No |
|  | 2 | 0.012 (8.4E-03-0.017) | 0.01 (0.01-0.01) | 0.01 (0.01-0.01) | 0.47 | 0.28 | 0.61 | NA | 1 | 1 | 1 | NA | No |
|  | 3 | 0.029 (0.013-0.061) | 0.022 (0.012-0.04) | 0.038 (5.3E-03-0.27) | 0.74 | 0.53 | 0.87 | 0.53 | 1 | 1 | 1 | 1 | No |
|  | 5 | 0.023 (0.012-0.045) | 0.049 (0.022-0.11) | 0.017 (5.5E-03-0.053) | 0.23 | 0.16 | 0.65 | 0.23 | 1 | 1 | 1 | 1 | No |
| *Cat cytokine response*  *above control^* |  |  |  |  |  |  |  |  |  |  |  |  |  |
| IL-13 protein (pg/ml)^ to cat | 0 | 2.8 (0.86-9.3) | 1.6 (0.48-5.2) | 1.3 (0.12-13) | 0.78 | 0.63 | 0.52 | 0.77 | 1 | 1 | 1 | 1 | No |
| to Fel d1 | 0 | 0.27 (0.057-1.3) | 0.24 (0.04-1.4) | 0.65 (2.9E-04-1.4E+03) | 0.97 | 0.97 | 0.84 | 0.86 | 1 | 1 | 1 | 1 | No |
|  | 5 | 0.022 (0.01-0.049) | 0.023 (0.011-0.047) | 0.054 (4.3E-03-0.66) | 0.61 | 0.98 | 0.38 | 0.37 | 1 | 1 | 1 | 1 | No |
| IL-5 protein (pg/ml)^ to cat | 0 | 0.29 (0.092-0.93) | 0.77 (0.25-2.4) | 1.2 (0.12-12) | 0.32 | 0.22 | 0.2 | 0.7 | 1 | 1 | 1 | 1 | No |
| to Fel d1 | 0 | 0.061 (0.018-0.21) | 0.038 (0.01-0.14) | 0.068 (1.5E-04-30) | 0.89 | 0.67 | 0.97 | 0.77 | 1 | 1 | 1 | 1 | No |
|  | 5 | 0.012 (8.2E-03-0.018) | 0.014 (8.8E-03-0.021) | 0.044 (4.8E-03-0.41) | *0.086* | 0.73 | **0.049** | *0.083* | 1 | 1 | 1 | 1 | No |
| IL-10 protein (pg/ml)^ to cat | 0 | 14 (5.2-36) | 8.5 (3.2-22) | 20 (4.1-101) | 0.64 | 0.47 | 0.83 | 0.42 | 1 | 1 | 1 | 1 | No |
| to Fel d1 | 0 | 0.1 (0.026-0.42) | 0.11 (0.021-0.52) | 0.01 (0.01-0.01) | 0.48 | 0.97 | 0.24 | 0.26 | 1 | 1 | 1 | 1 | No |
|  | 5 | 0.01 (0.01-0.01) | 0.01 (0.01-0.01) | 0.01 (0.01-0.01) | NA | NA | NA | NA | NA | NA | NA | NA | No |
| IFN-γ protein (pg/ml)^ to cat | 0 | 4.5 (1.2-16) | 4.5 (1.4-15) | 0.98 (0.075-13) | 0.44 | 0.87 | 0.21 | 0.25 | 1 | 1 | 1 | 1 | No |
| to Fel d1 | 0 | 0.091 (0.022-0.37) | 0.17 (0.029-1) | 0.66 (3.0E-04-1.5E+03) | 0.56 | 0.56 | 0.35 | 0.48 | 1 | 1 | 1 | 1 | No |
|  | 5 | 0.013 (7.7E-03-0.021) | 0.014 (8.5E-03-0.024) | 0.01 (0.01-0.01) | 0.78 | 0.74 | 0.63 | 0.54 | 1 | 1 | 1 | 1 | No |
| *Peanut cytokine response*  *above control^* |  |  |  |  |  |  |  |  |  |  |  |  |  |
| IL-13 protein (pg/ml)^ to Ara h2 | 6m | 0.01 (0.01-0.01) | 0.01 (0.01-0.01) | 0.033 (1.8E-03-0.63) | **7.8E-03** | NA | *0.059* | **0.018** | 0.16 | NA | 1 | 0.52 | No |
|  | 1 | 0.012 (8.2E-03-0.018) | 0.014 (8.7E-03-0.022) | 0.01 (0.01-0.01) | 0.69 | 0.65 | 0.58 | 0.45 | 1 | 1 | 1 | 1 | No |
| to peanut | 2 | 0.14 (0.04-0.47) | 0.05 (0.021-0.12) | 0.043 (4.9E-03-0.38) | 0.27 | 0.13 | 0.31 | 0.92 | 1 | 1 | 1 | 1 | No |
|  | 5 | 0.02 (0.01-0.04) | 0.033 (0.016-0.068) | 0.12 (0.011-1.3) | 0.15 | 0.36 | *0.052* | 0.18 | 1 | 1 | 1 | 1 | No |
| IL-5 protein (pg/ml)^ to Ara h2 | 6m | 0.01 (0.01-0.01) | 0.012 (8.1E-03-0.019) | 0.033 (1.8E-03-0.61) | 0.12 | 0.43 | *0.059* | 0.18 | 1 | 1 | 1 | 1 | No |
|  | 1 | 0.01 (0.01-0.01) | 0.01 (0.01-0.01) | 0.01 (0.01-0.01) | NA | NA | NA | NA | NA | NA | NA | NA | No |
| to peanut | 2 | 0.015 (8.6E-03-0.025) | 0.018 (0.01-0.032) | 0.023 (3.5E-03-0.16) | 0.8 | 0.62 | 0.55 | 0.78 | 1 | 1 | 1 | 1 | No |
|  | 5 | 0.01 (0.01-0.01) | 0.011 (8.8E-03-0.014) | 0.02 (4.5E-03-0.088) | 0.18 | 0.39 | *0.073* | 0.25 | 1 | 1 | 1 | 1 | No |
| IL-10 protein (pg/ml)^ to Ara h2 | 6m | 0.19 (0.04-0.89) | 0.21 (0.057-0.74) | 0.1 (2.6E-03-4.2) | 0.94 | 0.82 | 0.94 | 0.76 | 1 | 1 | 1 | 1 | No |
|  | 1 | 0.01 (0.01-0.01) | 0.012 (8.3E-03-0.017) | 0.01 (0.01-0.01) | 0.56 | 0.37 | NA | 0.61 | 1 | 1 | NA | 1 | No |
| to peanut | 2 | 0.01 (0.01-0.01) | 0.018 (0.01-0.031) | 0.01 (0.01-0.01) | 0.15 | *0.082* | NA | 0.38 | 1 | 1 | NA | 1 | No |
|  | 5 | 0.028 (0.013-0.06) | 0.017 (0.01-0.029) | 0.018 (5.2E-03-0.06) | 0.57 | 0.32 | 0.58 | 0.97 | 1 | 1 | 1 | 1 | No |
| IFN-γ protein (pg/ml)^ to Ara h2 | 6m | 3.3 (0.45-23) | 10 (2.4-45) | 1 (4.8E-03-212) | 0.64 | 0.4 | 0.86 | 0.57 | 1 | 1 | 1 | 1 | No |
|  | 1 | 0.012 (8.0E-03-0.02) | 0.014 (8.7E-03-0.023) | 0.01 (0.01-0.01) | 0.7 | 0.67 | 0.58 | 0.45 | 1 | 1 | 1 | 1 | No |
| to peanut | 2 | 0.012 (8.3E-03-0.017) | 0.042 (0.017-0.1) | 0.022 (3.8E-03-0.13) | *0.082* | **0.026** | 0.29 | 0.62 | 1 | 0.71 | 1 | 1 | No |
|  | 5 | 0.014 (8.7E-03-0.023) | 0.017 (0.01-0.027) | 0.066 (7.5E-03-0.58) | *0.074* | 0.66 | **0.04** | *0.063* | 1 | 1 | 1 | 1 | No |
| *Ovalbumin cytokine response*  *above control^* |  |  |  |  |  |  |  |  |  |  |  |  |  |
| IL-13 protein (pg/ml)^ | 0 | 1.2 (0.36-3.9) | 1.2 (0.39-3.5) | 0.13 (0.016-1.1) | 0.21 | 0.84 | *0.098* | *0.097* | 1 | 1 | 1 | 1 | No |
|  | 6m | 0.021 (9.0E-03-0.049) | 0.018 (9.3E-03-0.033) | 0.49 (0.014-17) | **3.3E-03** | 0.77 | **9.3E-03** | **1.9E-03** | *0.074* | 1 | 0.3 | *0.077* | No |
|  | 1 | 0.033 (0.013-0.083) | 0.03 (0.014-0.065) | 0.065 (7.6E-03-0.56) | 0.62 | 0.86 | 0.42 | 0.36 | 1 | 1 | 1 | 1 | No |
|  | 2 | 0.11 (0.033-0.35) | 0.055 (0.022-0.14) | 0.13 (6.8E-03-2.5) | 0.58 | 0.39 | 0.78 | 0.43 | 1 | 1 | 1 | 1 | No |
|  | 5 | 0.14 (0.047-0.41) | 0.12 (0.045-0.33) | 0.052 (7.8E-03-0.34) | 0.7 | 0.83 | 0.42 | 0.46 | 1 | 1 | 1 | 1 | No |
| IL-5 protein (pg/ml)^ | 0 | 0.13 (0.045-0.39) | 0.12 (0.043-0.34) | 0.077 (0.011-0.55) | 0.88 | 0.88 | 0.68 | 0.63 | 1 | 1 | 1 | 1 | No |
|  | 6m | 0.013 (7.8E-03-0.021) | 0.01 (0.01-0.01) | 0.055 (4.1E-03-0.74) | **5.1E-03** | 0.27 | *0.058* | **2.0E-03** | 0.11 | 1 | 1 | *0.08* | No |
|  | 1 | 0.01 (0.01-0.01) | 0.01 (0.01-0.01) | 0.01 (0.01-0.01) | NA | NA | NA | NA | NA | NA | NA | NA | No |
|  | 2 | 0.018 (9.2E-03-0.036) | 0.015 (9.4E-03-0.025) | 0.01 (0.01-0.01) | 0.67 | 0.71 | 0.4 | 0.46 | 1 | 1 | 1 | 1 | No |
|  | 5 | 0.014 (8.7E-03-0.023) | 0.02 (0.011-0.037) | 0.017 (5.5E-03-0.051) | 0.61 | 0.33 | 0.65 | 0.81 | 1 | 1 | 1 | 1 | No |
| IL-10 protein (pg/ml)^ | 0 | 1.5 (0.44-4.8) | 6 (2.2-16) | 6.8 (0.84-55) | 0.12 | *0.069* | 0.13 | 0.67 | 1 | 1 | 1 | 1 | No |
|  | 6m | 0.07 (0.02-0.25) | 0.14 (0.043-0.46) | 0.18 (6.4E-03-5.3) | 0.55 | 0.37 | 0.4 | 0.66 | 1 | 1 | 1 | 1 | No |
|  | 1 | 0.2 (0.056-0.7) | 0.33 (0.1-1.1) | 0.25 (0.018-3.3) | 0.73 | 0.42 | 0.8 | 0.85 | 1 | 1 | 1 | 1 | No |
|  | 2 | 0.031 (0.013-0.075) | 0.05 (0.021-0.12) | 0.023 (3.4E-03-0.16) | 0.6 | 0.41 | 0.83 | 0.48 | 1 | 1 | 1 | 1 | No |
|  | 5 | 3 (0.97-9.5) | 2.9 (0.95-8.7) | 1.1 (0.087-14) | 0.72 | 0.76 | 0.51 | 0.45 | 1 | 1 | 1 | 1 | No |
| IFN-γ protein (pg/ml)^ | 0 | 0.94 (0.23-3.8) | 1.2 (0.33-4.5) | 0.91 (0.074-11) | 0.94 | 0.88 | 0.78 | 0.74 | 1 | 1 | 1 | 1 | No |
|  | 6m | 0.19 (0.041-0.88) | 0.4 (0.11-1.5) | 0.21 (6.2E-03-7.4) | 0.79 | 0.48 | 0.82 | 0.94 | 1 | 1 | 1 | 1 | No |
|  | 1 | 0.3 (0.072-1.3) | 0.39 (0.11-1.4) | 0.49 (0.034-6.9) | 0.97 | 0.81 | 0.88 | 0.93 | 1 | 1 | 1 | 1 | No |
|  | 2 | 0.024 (0.01-0.056) | 0.082 (0.029-0.23) | 0.074 (3.6E-03-1.5) | 0.23 | *0.093* | 0.33 | 0.95 | 1 | 1 | 1 | 1 | No |
|  | 5 | 0.14 (0.041-0.44) | 0.25 (0.083-0.77) | 0.058 (7.7E-03-0.44) | 0.45 | 0.43 | 0.54 | 0.25 | 1 | 1 | 1 | 1 | No |
| *Ryegrass cytokine response*  *above control^* |  |  |  |  |  |  |  |  |  |  |  |  |  |
| IL-13 protein (pg/ml)^ | 2 | 0.52 (0.14-1.9) | 0.076 (0.029-0.2) | 1.1 (0.053-24) | **0.038** | **0.029** | 0.54 | **0.046** | 0.62 | 0.77 | 1 | 1 | No |
|  | 3 | 0.068 (0.023-0.2) | 0.059 (0.024-0.14) | 1.7 (0.12-25) | **5.2E-03** | 0.88 | **4.7E-03** | **2.7E-03** | 0.11 | 1 | 0.17 | 0.11 | No |
|  | 5 | 0.098 (0.036-0.26) | 0.087 (0.035-0.22) | 0.51 (0.042-6.1) | 0.27 | 0.86 | 0.16 | 0.11 | 1 | 1 | 1 | 1 | No |
| IL-5 protein (pg/ml)^ | 2 | 0.02 (0.01-0.041) | 0.017 (0.01-0.027) | 0.021 (4.0E-03-0.11) | 0.88 | 0.67 | 0.95 | 0.73 | 1 | 1 | 1 | 1 | No |
|  | 3 | 0.01 (0.01-0.01) | 0.015 (9.5E-03-0.023) | 0.062 (7.5E-03-0.52) | **6.1E-03** | 0.15 | **1.9E-03** | **0.039** | 0.13 | 1 | *0.077* | 0.98 | No |
|  | 5 | 0.02 (0.011-0.035) | 0.024 (0.013-0.045) | 0.045 (8.0E-03-0.25) | 0.48 | 0.65 | 0.22 | 0.41 | 1 | 1 | 1 | 1 | No |
| IL-10 protein (pg/ml)^ | 2 | 0.097 (0.033-0.29) | 0.077 (0.031-0.19) | 0.04 (5.0E-03-0.33) | 0.68 | 0.79 | 0.37 | 0.48 | 1 | 1 | 1 | 1 | No |
|  | 3 | 0.49 (0.13-1.8) | 1.8 (0.61-5.1) | 2.9 (0.23-38) | 0.18 | 0.14 | 0.13 | 0.38 | 1 | 1 | 1 | 1 | No |
|  | 5 | 0.14 (0.05-0.39) | 0.18 (0.067-0.48) | 0.27 (0.026-2.8) | 0.81 | 0.73 | 0.58 | 0.62 | 1 | 1 | 1 | 1 | No |
| IFN-γ protein (pg/ml)^ | 2 | 0.18 (0.052-0.64) | 0.28 (0.092-0.85) | 0.11 (6.8E-03-1.9) | 0.84 | 0.72 | 0.76 | 0.6 | 1 | 1 | 1 | 1 | No |
|  | 3 | 0.46 (0.12-1.8) | 1 (0.32-3.3) | 9.9 (0.66-148) | *0.061* | 0.25 | **0.017** | 0.11 | 0.9 | 1 | 0.5 | 1 | No |
|  | 5 | 0.092 (0.035-0.24) | 0.073 (0.029-0.18) | 0.11 (0.011-1.1) | 0.91 | 0.76 | 0.83 | 0.7 | 1 | 1 | 1 | 1 | No |

Feature? = whether variable was used as a clustering feature or not; geom. mean = geometric mean; HDM = house dust mite; PBMC = peripheral blood mononuclear cells; P-value (adj.) = adjusted P-values (Benjamini-Yekutieli method); prop. = proportion. For categorical variables, associations were tested using Fisher exact test; for continuous variables, Kruskal-Wallis and Mann-Whitney-Wilcoxon. Bold text indicates statistical significance (*p*<0.05); italics indicate near-significance (*p*<0.10). Note that none of these variables were used as clustering features. ^PBMC cytokine responses to HDM above unstimulated control; birth samples (age 0) taken from cord blood (CBMC).

1. **Immunological (SPT)**

| **Variable** | **Age** | **CAS1 (N=88)** | **CAS2 (N=107)** | **CAS3 (N=22)** | **P-value** |  |  |  | **P-value (adj.)** |  |  |  | **Feature?** |
| --- | --- | --- | --- | --- | --- | --- | --- | --- | --- | --- | --- | --- | --- |
|  |  | **Mean (95% CI)** | **Mean (95% CI)** | **Mean (95% CI)** | **Overall** | **1 vs. 2** | **1 vs. 3** | **2 vs. 3** | **Overall** | **1 vs. 2** | **1 vs. 3** | **2 vs. 3** |  |
| *Wheal size SPT* |  |  |  |  |  |  |  |  |  |  |  |  |  |
| Histamine (mm) | 6m | 1.8 (1.7-2) | 1.8 (1.7-1.9) | 1.9 (1.6-2.2) | 0.87 | 0.87 | 0.68 | 0.59 | 1 | 1 | 1 | 1 | **Yes** |
|  | 2 | 3.4 (3.1-3.8) | 3.4 (3.2-3.7) | 4.1 (3.1-5.1) | 0.4 | 0.66 | 0.2 | 0.24 | 1 | 1 | 1 | 1 | **Yes** |
|  | 5 | 2.5 (2.3-2.6) | 2.6 (2.5-2.8) | 2.8 (2.2-3.4) | 0.65 | 0.39 | 0.89 | 0.59 | 1 | 1 | 1 | 1 | No |
|  | 10 | 4.2 (3.9-4.4) | 4.2 (3.9-4.5) | 4.4 (3.8-5.1) | 0.6 | 0.78 | 0.41 | 0.32 | 1 | 1 | 1 | 1 | No |
| HDM (mm) | 6m | 0.091 (0-0.18) | 0.051 (0-0.12) | 0.34 (0-0.75) | **0.04** | 0.29 | 0.11 | 0.01 | 0.64 | 1 | 1 | 0.32 | **Yes** |
|  | 2 | 0.44 (0.17-0.72) | 0.67 (0.35-0.99) | 6.2 (4.5-7.9) | **5.9E-15** | 0.54 | **3.9E-13** | **1.4E-12** | **1.3E-12** | 1 | **2.6E-10** | **6.7E-10** | **Yes** |
|  | 5 | 1.1 (0.6-1.5) | 1.9 (1.4-2.5) | 5.5 (4.2-6.9) | **1.3E-08** | **0.026** | **5.9E-09** | **2.0E-06** | **7.6E-07** | 0.71 | **9.1E-07** | **1.7E-04** | No |
|  | 10 | 4.1 (3.4-4.8) | 4.8 (4.2-5.3) | 7 (4.9-9.1) | **2.2E-03** | **0.046** | **1.7E-03** | **0.016** | *0.051* | 1 | *0.07* | 0.47 | No |
| Cat (mm) | 6m | 0.091 (0-0.2) | 0.11 (0.012-0.21) | 0.32 (0-0.74) | 0.15 | 0.67 | 0.064 | 0.12 | 1 | 1 | 1 | 1 | **Yes** |
|  | 2 | 0.35 (0.18-0.51) | 0.4 (0.22-0.58) | 1.4 (0.56-2.3) | **9.6E-03** | 0.89 | **4.5E-03** | **5.8E-03** | 0.19 | 1 | 0.16 | 0.2 | **Yes** |
|  | 5 | 0.33 (0.16-0.49) | 0.56 (0.32-0.79) | 1.9 (0.6-3.2) | **4.0E-03** | 0.3 | **1.0E-03** | **9.7E-03** | *0.087* | 1 | **0.043** | 0.31 | No |
|  | 10 | 3.5 (2.9-4.1) | 4.6 (2.5-6.7) | 6.5 (0-15) | 0.36 | 0.79 | 0.2 | 0.27 | 1 | 1 | 1 | 1 | No |
| Ryegrass (mm) | 6m | 0.085 (0-0.2) | 0 (0-0) | 0.2 (0-0.47) | **1.8E-03** | *0.056* | *0.065* | **1.2E-04** | **0.043** | 1 | 1 | **6.6E-03** | **Yes** |
|  | 2 | 0.25 (0.1-0.4) | 0.22 (0.11-0.33) | 1.9 (0.82-2.9) | **5.0E-06** | 1 | **2.0E-05** | **7.4E-06** | **1.8E-04** | 1 | **1.3E-03** | **5.4E-04** | **Yes** |
|  | 5 | 0.37 (0.091-0.66) | 0.63 (0.33-0.92) | 3.1 (1.8-4.4) | **4.1E-09** | 0.11 | **2.8E-09** | **7.2E-07** | **2.6E-07** | 1 | **4.7E-07** | **6.7E-05** | No |
|  | 10 | 4.5 (3.8-5.2) | 3.6 (3-4.2) | 4.7 (3.8-5.7) | **0.025** | **0.021** | 0.66 | **0.033** | 0.43 | 0.6 | 1 | 0.86 | No |
| *Alternaria* (mm) | 6m | 0.057 (0-0.12) | 0.014 (0-0.042) | 0.11 (0-0.31) | *0.096* | 0.23 | 0.27 | **0.022** | 1 | 1 | 1 | 0.62 | **Yes** |
|  | 2 | 0.14 (0.031-0.25) | 0.089 (0-0.19) | 0.091 (0-0.28) | 0.59 | 0.34 | 0.59 | 1 | 1 | 1 | 1 | 1 | **Yes** |
|  | 5 | 0.13 (0-0.26) | 0.41 (0.2-0.62) | 0.36 (0-0.77) | *0.059* | **0.018** | 0.14 | 0.82 | 0.89 | 0.52 | 1 | 1 | No |
| *Aspergillus* (mm) | 6m | 0.023 (0-0.054) | 0.061 (5.4E-03-0.12) | 0.45 (0.1-0.81) | **3.0E-05** | 0.36 | **4.6E-05** | **4.5E-04** | **9.9E-04** | 1 | **2.8E-03** | **0.021** | **Yes** |
|  | 2 | 0.27 (0.13-0.42) | 0.14 (0.046-0.23) | 0.52 (0-1.1) | 0.2 | 0.15 | 0.56 | 0.13 | 1 | 1 | 1 | 1 | **Yes** |
|  | 5 | 0.18 (0.031-0.34) | 0.35 (0.054-0.65) | 0.6 (0-1.2) | 0.24 | 0.57 | *0.091* | 0.2 | 1 | 1 | 1 | 1 | No |
| Cow’s milk (mm) | 6m | 0.18 (0.014-0.35) | 0.43 (0.13-0.74) | 1.8 (0.65-2.9) | **2.4E-04** | 0.26 | **8.2E-05** | **1.3E-03** | **6.8E-03** | 1 | **4.7E-03** | *0.055* | **Yes** |
|  | 2 | 0.23 (0.097-0.37) | 0.26 (0.093-0.42) | 2.5 (0.98-3.9) | **1.8E-06** | 0.8 | **1.0E-05** | **3.5E-06** | **7.5E-05** | 1 | **7.2E-04** | **2.8E-04** | **Yes** |
|  | 5 | 0 (0-0) | 0.022 (0-0.066) | 0.78 (0-1.8) | **2.0E-06** | 0.35 | **5.7E-05** | **2.7E-04** | **8.2E-05** | 1 | **3.4E-03** | **0.014** | No |
| Egg white (mm) | 6m | 0.41 (0.085-0.73) | 1.1 (0.54-1.7) | 4.7 (2.5-6.9) | **1.1E-06** | 0.2 | **3.7E-07** | **4.7E-05** | **4.6E-05** | 1 | **3.7E-05** | **2.9E-03** | **Yes** |
|  | 2 | 0.24 (0-0.51) | 0.78 (0.34-1.2) | 5.8 (3-8.5) | **2.6E-09** | **0.021** | **1.3E-09** | **2.5E-06** | **1.7E-07** | 0.6 | **2.3E-07** | **2.1E-04** | **Yes** |
|  | 5 | 0 (0-0) | 0.094 (0-0.2) | 2.3 (0.97-3.6) | **3.9E-16** | 0.1 | **1.4E-11** | **1.0E-09** | **1.1E-13** | 1 | **4.7E-09** | **1.8E-07** | No |
| Peanut (mm) | 5 | 0.013 (0-0.038) | 0.26 (0.076-0.45) | 1.5 (0.063-2.9) | **1.7E-04** | **0.026** | **1.2E-05** | **0.011** | **5.0E-03** | 0.71 | **8.3E-04** | 0.34 | No |
|  |  | **Prop. (95% CI)** | **Prop. (95% CI)** | **Prop. (95% CI)** | **Overall** | **1 vs. 2** | **1 vs. 3** | **2 vs. 3** | **Overall** | **1 vs. 2** | **1 vs. 3** | **2 vs. 3** |  |
| *SPT past atopy threshold* |  |  |  |  |  |  |  |  |  |  |  |  |  |
| Histamine wheal ≥ 2mm | 6m | 57% (46%-67%) | 55% (46%-65%) | 64% (42%-85%) | 0.78 | 0.89 | 0.63 | 0.49 | 1 | 1 | 1 | 1 | No* |
|  | 2 | 89% (82%-95%) | 97% (94%-100%) | 95% (86%-100%) | **0.038** | **0.021** | 0.69 | 0.53 | 0.62 | 0.6 | 1 | 1 | No* |
| ≥ 3mm | 5 | 30% (20%-41%) | 39% (29%-49%) | 29% (7.5%-50%) | 0.44 | 0.26 | 1 | 0.46 | 1 | 1 | 1 | 1 | No |
|  | 10 | 98% (95%-100%) | 100% (100%-100%) | 100% (100%-100%) | 0.53 | 0.46 | 1 | 1 | 1 | 1 | 1 | 1 | No |
| HDM wheal ≥ 2mm | 6m | 2.3% (0%-5.4%) | 1.9% (0%-4.5%) | 14% (0%-29%) | **0.043** | 1 | *0.054* | **0.035** | 0.68 | 1 | 1 | 0.9 | No* |
|  | 2 | 10% (3.8%-17%) | 15% (8.1%-22%) | 86% (71%-100%) | **2.9E-12** | 0.39 | **8.2E-12** | **1.5E-10** | **3.7E-10** | 1 | **3.0E-09** | **3.7E-08** | No* |
| ≥ 3mm | 5 | 13% (5.2%-20%) | 28% (18%-37%) | 81% (63%-99%) | **1.5E-08** | **0.022** | **4.6E-09** | **1.0E-05** | **8.7E-07** | 0.62 | **7.2E-07** | **7.2E-04** | No |
|  | 10 | 36% (23%-49%) | 51% (38%-63%) | 78% (57%-99%) | **7.4E-03** | 0.11 | **2.7E-03** | *0.06* | 0.15 | 1 | 0.11 | 1 | No |
| Cat wheal ≥ 2mm | 6m | 3.4% (0%-7.3%) | 4.7% (0.61%-8.7%) | 9.1% (0%-22%) | 0.4 | 0.73 | 0.26 | 0.34 | 1 | 1 | 1 | 1 | No* |
|  | 2 | 14% (6.3%-21%) | 14% (7.3%-21%) | 41% (19%-63%) | **0.011** | 1 | 0.012 | **6.3E-03** | 0.22 | 1 | 0.37 | 0.22 | No* |
| ≥ 3mm | 5 | 0% (0%-0%) | 5.6% (0.73%-10%) | 25% (4.2%-46%) | **1.9E-04** | *0.061* | **2.2E-04** | **0.017** | **5.5E-03** | 1 | **0.011** | 0.5 | No |
|  | 10 | 6.9% (0.18%-14%) | 12% (4%-20%) | 17% (0%-36%) | 0.4 | 0.38 | 0.35 | 0.69 | 1 | 1 | 1 | 1 | No |
| Ryegrass wheal ≥ 2mm | 6m | 1.1% (0%-3.4%) | 0% (0%-0%) | 4.5% (0%-14%) | *0.092* | 0.45 | 0.36 | 0.17 | 1 | 1 | 1 | 1 | No* |
|  | 2 | 10% (3.8%-17%) | 7.5% (2.4%-13%) | 45% (23%-68%) | **7.1E-05** | 0.61 | **4.3E-04** | **5.2E-05** | **2.3E-03** | 1 | **0.02** | **3.1E-03** | No* |
| ≥ 3mm | 5 | 3.8% (0%-8.1%) | 7.8% (2.1%-13%) | 50% (26%-74%) | **2.2E-06** | 0.34 | **2.4E-06** | **3.9E-05** | **8.8E-05** | 1 | **2.0E-04** | **2.4E-03** | No |
|  | 10 | 21% (9.9%-31%) | 24% (13%-34%) | 56% (30%-81%) | **0.017** | 0.83 | **7.3E-03** | **0.019** | 0.31 | 1 | 0.24 | 0.55 | No |
| *Alternaria* wheal ≥ 2mm | 6m | 2.3% (0%-5.4%) | 0% (0%-0%) | 4.5% (0%-14%) | *0.078* | 0.2 | 0.49 | 0.17 | 1 | 1 | 1 | 1 | No* |
|  | 2 | 4.5% (0.11%-9%) | 0.93% (0%-2.8%) | 4.5% (0%-14%) | 0.23 | 0.18 | 1 | 0.31 | 1 | 1 | 1 | 1 | No* |
| ≥ 3mm | 5 | 1.3% (0%-3.8%) | 6.7% (1.4%-12%) | 4.8% (0%-15%) | 0.16 | 0.12 | 0.38 | 1 | 1 | 1 | 1 | 1 | No |
| *Aspergillus* wheal ≥ 2mm | 6m | 0% (0%-0%) | 0.93% (0%-2.8%) | 18% (0.68%-36%) | **3.8E-04** | 1 | **1.3E-03** | **2.9E-03** | **0.01** | 1 | *0.055* | 0.11 | No* |
|  | 2 | 10% (3.8%-17%) | 4.7% (0.61%-8.7%) | 14% (0%-29%) | 0.15 | 0.17 | 0.7 | 0.14 | 1 | 1 | 1 | 1 | No* |
| ≥ 3mm | 5 | 1.3% (0%-3.8%) | 4.4% (0.1%-8.8%) | 15% (0%-32%) | **0.032** | 0.37 | **0.025** | 0.11 | 0.53 | 1 | 0.69 | 1 | No |
| Cow’s milk wheal ≥ 2mm | 6m | 3.4% (0%-7.3%) | 7.5% (2.4%-13%) | 36% (15%-58%) | **1.2E-04** | 0.35 | **8.7E-05** | **1.1E-03** | **3.7E-03** | 1 | **5.0E-03** | **0.047** | No* |
|  | 2 | 9.1% (3%-15%) | 6.5% (1.8%-11%) | 50% (27%-73%) | **4.7E-06** | 0.59 | **5.1E-05** | **4.5E-06** | **1.7E-04** | 1 | **3.1E-03** | **3.5E-04** | No* |
| ≥ 3mm | 5 | 0% (0%-0%) | 0% (0%-0%) | 5% (0%-15%) | 0.11 | 1 | 0.2 | 0.18 | 1 | 1 | 1 | 1 | No |
| Egg white wheal ≥ 2mm | 6m | 6.8% (1.4%-12%) | 15% (8.1%-22%) | 55% (32%-77%) | **4.6E-06** | 0.11 | **1.9E-06** | **1.9E-04** | **1.7E-04** | 1 | **1.6E-04** | **0.01** | No* |
|  | 2 | 5.7% (0.75%-11%) | 14% (7.3%-21%) | 59% (37%-81%) | **1.8E-07** | *0.062* | **1.0E-07** | **2.4E-05** | **8.6E-06** | 1 | **1.2E-05** | **1.6E-03** | No* |
| ≥ 3mm | 5 | 0% (0%-0%) | 2.2% (0%-5.3%) | 29% (7.5%-50%) | **1.1E-05** | 0.5 | **4.6E-05** | **5.2E-04** | **3.9E-04** | 1 | **2.8E-03** | **0.024** | No |
| Any SPT wheal ≥ 2mm | 6m | 16% (8.1%-24%) | 21% (14%-29%) | 64% (42%-85%) | **4.4E-05** | 0.36 | **2.1E-05** | **1.8E-04** | **1.4E-03** | 1 | **1.4E-03** | **9.6E-03** | No* |
|  | 2 | 34% (24%-44%) | 36% (27%-46%) | 100% (100%-100%) | **3.7E-09** | 0.77 | **3.6E-09** | **6.1E-09** | **2.3E-07** | 1 | **5.9E-07** | **9.2E-07** | No* |
| ≥ 3mm | 5 | 16% (8.1%-25%) | 38% (28%-48%) | 90% (77%-100%) | **8.5E-10** | **2.1E-03** | **4.0E-10** | **1.3E-05** | **6.2E-08** | *0.084* | **8.6E-08** | **9.0E-04** | No |
|  | 10 | 52% (38%-65%) | 60% (48%-72%) | 89% (73%-100%) | **0.014** | 0.37 | **5.4E-03** | **0.026** | 0.26 | 1 | 0.19 | 0.71 | No |

Feature? = whether variable was used as a clustering feature or not; geom. mean = geometric mean; HDM = house dust mite; P-value (adj.) = adjusted P-values (Benjamini-Yekutieli method); SPT = skin prick or sensitisation test. For categorical variables, associations were tested using Fisher exact test; for continuous variables, Kruskal-Wallis and Mann-Whitney-Wilcoxon. Bold text indicates statistical significance (*p*<0.05); italics indicate near-significance (*p*<0.10). *Not used as clustering features, as these were derived variables.

1. **Microbiological and respiratory health-related**

| **Variable** | **Age (y)** | **CAS1 (N=88)** | **CAS2 (N=107)** | **CAS3 (N=22)** | **P-value** |  |  |  | **P-value (adj.)** |  |  |  | **Feature?** |
| --- | --- | --- | --- | --- | --- | --- | --- | --- | --- | --- | --- | --- | --- |
|  |  | **Mean (95% CI)** | **Mean (95% CI)** | **Mean (95% CI)** | **Overall** | **1 vs. 2** | **1 vs. 3** | **2 vs. 3** | **Overall** | **1 vs. 2** | **1 vs. 3** | **2 vs. 3** |  |
| *Events in general* |  |  |  |  |  |  |  |  |  |  |  |  |  |
| Any ARI (events per y) | 1 | 4.4 (3.9-4.9) | 3.6 (3.1-4.1) | 4.5 (3.3-5.6) | **0.044** | **0.018** | 0.86 | 0.16 | 0.69 | 0.52 | 1 | 1 | No* |
|  | 2 | 4.5 (3.8-5.2) | 3.6 (3.2-4) | 4.7 (3.4-6) | 0.13 | 0.13 | 0.56 | **0.077** | 1 | 1 | 1 | 1 | No* |
|  | 3 | 3.7 (3.1-4.3) | 3.4 (3-3.9) | 4 (2.7-5.4) | 0.74 | 0.56 | 0.77 | 0.53 | 1 | 1 | 1 | 1 | No* |
|  | 4 | 3 (2.4-3.6) | 2.7 (2.2-3.2) | 3.7 (2.4-4.9) | 0.28 | 0.48 | 0.28 | 0.11 | 1 | 1 | 1 | 1 | No |
|  | 5 | 2 (1.5-2.5) | 1.9 (1.5-2.3) | 1.5 (0.9-2.1) | 0.94 | 0.83 | 0.89 | 0.74 | 1 | 1 | 1 | 1 | No |
| URI (events per y) | 1 | 2.9 (2.4-3.3) | 2.6 (2.2-3) | 2.5 (1.7-3.3) | 0.59 | 0.34 | 0.5 | 0.96 | 1 | 1 | 1 | 1 | **Yes** |
|  | 2 | 3.2 (2.6-3.7) | 2.6 (2.2-3) | 2.5 (1.2-3.8) | 0.19 | 0.19 | 0.12 | 0.34 | 1 | 1 | 1 | 1 | **Yes** |
|  | 3 | 2.7 (2.2-3.2) | 2.8 (2.4-3.3) | 2.2 (1.3-3.2) | 0.45 | 0.41 | 0.59 | 0.24 | 1 | 1 | 1 | 1 | **Yes** |
|  | 4 | 2.1 (1.7-2.6) | 2.2 (1.8-2.7) | 1.7 (0.77-2.7) | 0.5 | 0.94 | 0.26 | 0.27 | 1 | 1 | 1 | 1 | No |
|  | 5 | 1.6 (1.1-2) | 1.5 (1.2-1.9) | 0.67 (0.2-1.1) | *0.081* | 0.76 | **0.047** | **0.026** | 1 | 1 | 1 | 0.71 | No |
| LRI (events per y) | 1 | 1.6 (1.2-1.9) | 0.98 (0.76-1.2) | 2 (1.3-2.6) | **4.0E-03** | **0.021** | 0.17 | **2.6E-03** | *0.087* | 0.6 | 1 | 0.1 | **Yes** |
|  | 2 | 1.4 (0.98-1.7) | 1 (0.81-1.2) | 2.2 (1.6-2.9) | **2.5E-03** | 0.83 | **6.1E-03** | **2.0E-04** | *0.058* | 1 | 0.21 | **0.011** | **Yes** |
|  | 3 | 1 (0.76-1.3) | 0.6 (0.4-0.8) | 1.8 (1.1-2.6) | **6.1E-04** | **0.02** | **0.039** | **2.7E-04** | **0.016** | 0.57 | 0.98 | **0.014** | **Yes** |
|  | 4 | 0.87 (0.52-1.2) | 0.46 (0.3-0.63) | 2 (1.1-2.8) | **1.7E-05** | 0.3 | **3.5E-04** | **1.6E-06** | **5.9E-04** | 1 | **0.017** | **1.4E-04** | No |
|  | 5 | 0.42 (0.24-0.6) | 0.36 (0.24-0.48) | 0.86 (0.44-1.3) | **0.019** | 1 | **0.011** | **7.5E-03** | 0.34 | 1 | 0.34 | 0.25 | No |
| Wheezy LRI (wLRI, events per y) | 1 | 0.47 (0.3-0.63) | 0.24 (0.15-0.34) | 0.64 (0.19-1.1) | *0.054* | **0.036** | 0.61 | *0.065* | 0.82 | 0.93 | 1 | 1 | **Yes** |
|  | 2 | 0.68 (0.45-0.91) | 0.41 (0.26-0.56) | 1 (0.56-1.5) | **5.2E-03** | *0.063* | *0.066* | **1.7E-03** | 0.11 | 1 | 1 | *0.07* | **Yes** |
|  | 3 | 0.59 (0.37-0.81) | 0.3 (0.17-0.44) | 1.4 (0.78-2.1) | **4.6E-05** | *0.065* | **2.5E-03** | **6.6E-06** | **1.5E-03** | 1 | *0.098* | **4.9E-04** | **Yes** |
|  | 4 | 0.52 (0.25-0.79) | 0.32 (0.18-0.46) | 1.9 (0.95-2.8) | **4.5E-08** | 0.86 | **9.3E-07** | **3.3E-08** | **2.4E-06** | 1 | **8.5E-05** | **4.3E-06** | No |
|  | 5 | 0.28 (0.13-0.42) | 0.23 (0.13-0.33) | 0.76 (0.36-1.2) | **2.3E-03** | 0.99 | **2.0E-03** | **1.2E-03** | *0.053* | 1 | *0.08* | *0.051* | No |
| Febrile LRI (fLRI, events per y) | 1 | 0.36 (0.22-0.51) | 0.28 (0.16-0.4) | 0.55 (0.28-0.81) | **0.025** | 0.24 | *0.071* | **6.4E-03** | 0.43 | 1 | 1 | 0.22 | **Yes** |
|  | 2 | 0.36 (0.23-0.5) | 0.33 (0.22-0.43) | 0.95 (0.46-1.4) | **0.01** | 1 | **6.1E-03** | **3.8E-03** | 0.2 | 1 | 0.21 | 0.14 | **Yes** |
|  | 3 | 0.38 (0.21-0.55) | 0.16 (0.09-0.23) | 0.52 (0.13-0.92) | *0.06* | *0.063* | 0.44 | 0.04 | 0.89 | 1 | 1 | 1 | **Yes** |
|  | 4 | 0.3 (0.13-0.47) | 0.15 (0.064-0.24) | 0.43 (0.16-0.7) | **0.021** | 0.18 | *0.091* | **4.9E-03** | 0.37 | 1 | 1 | 0.17 | No |
|  | 5 | 0.19 (0.082-0.3) | 0.14 (0.06-0.21) | 0.19 (0-0.42) | 0.83 | 0.55 | 0.91 | 0.8 | 1 | 1 | 1 | 1 | No |
| Severe LRI (wLRI or fLRI, events per y) | 1 | 0.69 (0.5-0.89) | 0.44 (0.29-0.58) | 1 (0.49-1.5) | **0.012** | **0.027** | 0.25 | **9.1E-03** | 0.23 | 0.73 | 1 | 0.29 | No* |
|  | 2 | 0.9 (0.62-1.2) | 0.59 (0.43-0.75) | 1.6 (1.1-2.2) | **7.9E-04** | 0.22 | **5.2E-03** | **1.2E-04** | **0.02** | 1 | 0.18 | **6.6E-03** | No* |
|  | 3 | 0.73 (0.49-0.97) | 0.37 (0.23-0.51) | 1.5 (0.85-2.2) | **1.6E-04** | **0.032** | **0.01** | **3.8E-05** | **4.8E-03** | 0.84 | 0.32 | **2.4E-03** | No* |
|  | 4 | 0.63 (0.32-0.94) | 0.36 (0.21-0.52) | 1.9 (1-2.8) | **2.8E-07** | 0.56 | **5.9E-06** | **8.4E-08** | **1.3E-05** | 1 | **4.5E-04** | **1.0E-05** | No |
|  | 5 | 0.36 (0.19-0.53) | 0.27 (0.17-0.38) | 0.76 (0.36-1.2) | **0.015** | 0.88 | **0.012** | **5.0E-03** | 0.28 | 1 | 0.37 | 0.18 | No |
| *Events with RSV detected in sample* |  |  |  |  |  |  |  |  |  |  |  |  |  |
| URI (events per y) | 1 | 0.22 (0.13-0.3) | 0.24 (0.15-0.33) | 0.14 (0-0.34) | 0.41 | 0.84 | 0.22 | 0.19 | 1 | 1 | 1 | 1 | **Yes** |
|  | 2 | 0.11 (0.032-0.2) | 0.25 (0.16-0.34) | 0.14 (0-0.31) | **0.033** | **0.01** | 0.51 | 0.35 | 0.54 | 0.32 | 1 | 1 | **Yes** |
|  | 3 | 0.15 (0.068-0.24) | 0.18 (0.089-0.27) | 0.14 (0-0.36) | 0.82 | 0.82 | 0.63 | 0.54 | 1 | 1 | 1 | 1 | **Yes** |
| LRI (events per y) | 1 | 0.28 (0.18-0.39) | 0.12 (0.059-0.18) | 0.36 (0.15-0.58) | **5.6E-03** | **7.1E-03** | 0.43 | **5.3E-03** | 0.12 | 0.24 | 1 | 0.19 | **Yes** |
|  | 2 | 0.15 (0.072-0.22) | 0.16 (0.084-0.23) | 0.24 (0.039-0.44) | 0.57 | 0.95 | 0.32 | 0.33 | 1 | 1 | 1 | 1 | **Yes** |
|  | 3 | 0.13 (0.049-0.21) | 0.076 (0.018-0.13) | 0.29 (0.075-0.5) | **0.015** | 0.23 | *0.061* | **3.1E-03** | 0.28 | 1 | 1 | 0.12 | **Yes** |
| wLRI (events per y) | 1 | 0.057 (7.5E-03-0.11) | 0.028 (0-0.06) | 0.18 (6.8E-03-0.36) | **0.016** | 0.32 | *0.058* | **4.0E-03** | 0.29 | 1 | 1 | 0.15 | **Yes** |
|  | 2 | 0.091 (0.03-0.15) | 0.13 (0.066-0.2) | 0.048 (0-0.15) | 0.44 | 0.38 | 0.52 | 0.28 | 1 | 1 | 1 | 1 | **Yes** |
|  | 3 | 0.047 (0-0.1) | 0.029 (0-0.061) | 0.24 (0.039-0.44) | **3.2E-04** | 0.79 | **2.1E-03** | **3.5E-04** | **8.7E-03** | 1 | *0.084* | **0.017** | **Yes** |
| fLRI (events per y) | 1 | 0.1 (0.038-0.17) | 0.037 (8.5E-04-0.074) | 0.18 (6.8E-03-0.36) | **0.04** | *0.072* | 0.31 | **0.011** | 0.64 | 1 | 1 | 0.34 | **Yes** |
|  | 2 | 0.08 (0.022-0.14) | 0.093 (0.032-0.16) | 0.24 (0.039-0.44) | *0.075* | 0.9 | **0.039** | **0.043** | 1 | 1 | 0.98 | 1 | **Yes** |
|  | 3 | 0.082 (0.023-0.14) | 0.019 (0-0.046) | 0.095 (0-0.23) | *0.097* | **0.042** | 0.86 | *0.072* | 1 | 1 | 1 | 1 | **Yes** |
| *Events with influenza detected in sample* |  |  |  |  |  |  |  |  |  |  |  |  |  |
| URI (events per y) | 1 | 0.12 (0.048-0.2) | 0.13 (0.066-0.2) | 0.045 (0-0.14) | 0.53 | 0.74 | 0.34 | 0.26 | 1 | 1 | 1 | 1 | **Yes** |
|  | 2 | 0.091 (0.03-0.15) | 0.075 (0.024-0.13) | 0.19 (0-0.42) | 0.57 | 0.69 | 0.45 | 0.29 | 1 | 1 | 1 | 1 | **Yes** |
|  | 3 | 0.059 (7.8E-03-0.11) | 0.038 (8.7E-04-0.075) | 0.048 (0-0.15) | 0.8 | 0.51 | 0.85 | 0.85 | 1 | 1 | 1 | 1 | **Yes** |
| LRI (events per y) | 1 | 0.057 (0-0.12) | 0.028 (0-0.06) | 0.091 (0-0.22) | 0.4 | 0.51 | 0.42 | 0.17 | 1 | 1 | 1 | 1 | **Yes** |
|  | 2 | 0.034 (0-0.073) | 0.037 (8.5E-04-0.074) | 0.095 (0-0.23) | 0.43 | 0.91 | 0.24 | 0.26 | 1 | 1 | 1 | 1 | **Yes** |
|  | 3 | 0.035 (0-0.075) | 9.5E-03 (0-0.028) | 0.048 (0-0.15) | 0.38 | 0.22 | 0.8 | 0.21 | 1 | 1 | 1 | 1 | **Yes** |
| wLRI (events per y) | 1 | 0.023 (0-0.054) | 0 (0-0) | 0.045 (0-0.14) | 0.16 | 0.12 | 0.57 | **0.029** | 1 | 1 | 1 | 0.77 | **Yes** |
|  | 2 | 0.034 (0-0.073) | 0 (0-0) | 0.048 (0-0.15) | 0.13 | *0.056* | 0.78 | **0.025** | 1 | 1 | 1 | 0.69 | **Yes** |
|  | 3 | 0.024 (0-0.056) | 9.5E-03 (0-0.028) | 0.048 (0-0.15) | 0.47 | 0.45 | 0.56 | 0.21 | 1 | 1 | 1 | 1 | **Yes** |
| fLRI (events per y) | 1 | 0.034 (0-0.073) | 0.019 (0-0.045) | 0.045 (0-0.14) | 0.7 | 0.5 | 0.81 | 0.46 | 1 | 1 | 1 | 1 | **Yes** |
|  | 2 | 0.011 (0-0.034) | 0.019 (0-0.045) | 0.095 (0-0.23) | *0.066* | 0.68 | **0.037** | *0.068* | 0.97 | 1 | 0.94 | 1 | **Yes** |
|  | 3 | 0.035 (0-0.075) | 9.5E-03 (0-0.028) | 0 (0-0) | 0.35 | 0.22 | 0.39 | 0.67 | 1 | 1 | 1 | 1 | **Yes** |
| *Events with HRV-A detected in sample* |  |  |  |  |  |  |  |  |  |  |  |  |  |
| LRI (events per y) | 1 | 0.55 (0.33-0.76) | 0.36 (0.24-0.49) | 0.73 (0.27-1.2) | 0.22 | 0.37 | 0.29 | *0.089* | 1 | 1 | 1 | 1 | **Yes** |
|  | 2 | 0.32 (0.2-0.44) | 0.23 (0.14-0.33) | 0.38 (0.11-0.65) | 0.29 | 0.23 | 0.58 | 0.18 | 1 | 1 | 1 | 1 | **Yes** |
|  | 3 | 0.27 (0.14-0.41) | 0.095 (0.032-0.16) | 0.29 (0.031-0.54) | **0.048** | **0.031** | 0.68 | **0.042** | 0.74 | 0.81 | 1 | 1 | **Yes** |
| wLRI (events per y) | 1 | 0.14 (0.063-0.21) | 0.15 (0.071-0.23) | 0.23 (0-0.46) | 0.79 | 0.95 | 0.54 | 0.51 | 1 | 1 | 1 | 1 | **Yes** |
|  | 2 | 0.17 (0.09-0.25) | 0.084 (0.025-0.14) | 0.19 (0-0.42) | 0.13 | **0.043** | 0.83 | 0.3 | 1 | 1 | 1 | 1 | **Yes** |
|  | 3 | 0.14 (0.052-0.23) | 0.057 (0.012-0.1) | 0.24 (0-0.48) | *0.098* | 0.13 | 0.38 | **0.037** | 1 | 1 | 1 | 0.94 | **Yes** |
| fLRI (events per y) | 1 | 0.12 (0.048-0.2) | 0.1 (0.044-0.16) | 0.14 (0-0.34) | 0.95 | 0.79 | 0.81 | 0.92 | 1 | 1 | 1 | 1 | **Yes** |
|  | 2 | 0.068 (5.6E-03-0.13) | 0.047 (6.1E-03-0.087) | 0.14 (0-0.31) | 0.24 | 0.74 | 0.19 | *0.099* | 1 | 1 | 1 | 1 | **Yes** |
|  | 3 | 0.071 (5.8E-03-0.14) | 0.038 (8.7E-04-0.075) | 0.095 (0-0.29) | 0.79 | 0.5 | 0.88 | 0.82 | 1 | 1 | 1 | 1 | **Yes** |
| *Events with HRV-B detected in sample* |  |  |  |  |  |  |  |  |  |  |  |  |  |
| LRI (events per y) | 1 | 0.068 (0.014-0.12) | 0.019 (0-0.045) | 0.091 (0-0.22) | 0.15 | *0.084* | 0.72 | *0.078* | 1 | 1 | 1 | 1 | **Yes** |
|  | 2 | 0.011 (0-0.034) | 0 (0-0) | 0.048 (0-0.15) | 0.11 | 0.27 | 0.28 | **0.025** | 1 | 1 | 1 | 0.69 | **Yes** |
|  | 3 | 0.024 (0-0.056) | 0 (0-0) | 0.095 (0-0.23) | **0.013** | 0.12 | 0.13 | **1.6E-03** | 0.25 | 1 | 1 | *0.066* | **Yes** |
| wLRI (events per y) | 1 | 0.011 (0-0.034) | 0 (0-0) | 0.045 (0-0.14) | 0.12 | 0.27 | 0.29 | **0.029** | 1 | 1 | 1 | 0.77 | **Yes** |
|  | 2 | 0 (0-0) | 0 (0-0) | 0 (0-0) | NA | NA | NA | NA | NA | NA | NA | NA | No^ |
|  | 3 | 0.012 (0-0.035) | 0 (0-0) | 0.048 (0-0.15) | 0.12 | 0.27 | 0.29 | **0.027** | 1 | 1 | 1 | 0.73 | **Yes** |
| fLRI (events per y) | 1 | 0.034 (0-0.073) | 0 (0-0) | 0.045 (0-0.14) | 0.13 | *0.056* | 0.81 | **0.029** | 1 | 1 | 1 | 0.77 | **Yes** |
|  | 2 | 0.011 (0-0.034) | 0 (0-0) | 0.048 (0-0.15) | 0.11 | 0.27 | 0.28 | **0.025** | 1 | 1 | 1 | 0.69 | **Yes** |
|  | 3 | 0.012 (0-0.035) | 0 (0-0) | 0.048 (0-0.15) | 0.12 | 0.27 | 0.29 | **0.027** | 1 | 1 | 1 | 0.73 | **Yes** |
| *Events with HRV-C detected in sample* |  |  |  |  |  |  |  |  |  |  |  |  |  |
| LRI (events per y) | 1 | 0.51 (0.35-0.67) | 0.29 (0.18-0.4) | 0.59 (0.14-1) | *0.068* | **0.027** | 0.99 | 0.17 | 1 | 0.73 | 1 | 1 | **Yes** |
|  | 2 | 0.35 (0.19-0.51) | 0.23 (0.14-0.33) | 0.67 (0.3-1) | **0.021** | 0.63 | **0.027** | **5.1E-03** | 0.37 | 1 | 0.73 | 0.18 | **Yes** |
|  | 3 | 0.25 (0.13-0.37) | 0.14 (0.07-0.22) | 0.57 (0.2-0.94) | **0.014** | 0.26 | **0.046** | **3.3E-03** | 0.26 | 1 | 1 | 0.13 | **Yes** |
| wLRI (events per y) | 1 | 0.15 (0.06-0.24) | 0.075 (0.024-0.13) | 0.23 (0-0.46) | 0.23 | 0.23 | 0.48 | 0.11 | 1 | 1 | 1 | 1 | No |
|  | 2 | 0.14 (0.044-0.23) | 0.084 (0.014-0.15) | 0.38 (0.076-0.69) | **9.0E-03** | 0.34 | **0.03** | **2.1E-03** | 0.18 | 1 | 0.79 | *0.084* | No |
|  | 3 | 0.16 (0.06-0.27) | 0.067 (0.011-0.12) | 0.48 (0.13-0.82) | **8.4E-04** | 0.13 | **0.016** | **1.2E-04** | **0.021** | 1 | 0.48 | **6.6E-03** | No |
| fLRI (events per y) | 1 | 0.091 (0.03-0.15) | 0.075 (0.018-0.13) | 0.091 (0-0.22) | 0.8 | 0.52 | 1 | 0.69 | 1 | 1 | 1 | 1 | **Yes** |
|  | 2 | 0.057 (0-0.12) | 0.047 (6.1E-03-0.087) | 0.048 (0-0.15) | 1 | 0.98 | 0.98 | 0.99 | 1 | 1 | 1 | 1 | **Yes** |
|  | 3 | 0.047 (1.1E-03-0.093) | 0.029 (0-0.061) | 0.14 (0-0.31) | *0.08* | 0.5 | 0.12 | **0.026** | 1 | 1 | 1 | 0.71 | **Yes** |
|  |  | **Prop. (95% CI)** | **Prop. (95% CI)** | **Prop. (95% CI)** | **Overall** | **1 vs. 2** | **1 vs. 3** | **2 vs. 3** | **Overall** | **1 vs. 2** | **1 vs. 3** | **2 vs. 3** |  |
| *Proportion of ARI events* |  |  |  |  |  |  |  |  |  |  |  |  |  |
| wLRI / ARI | 1 | 11% (7%-16%) | 7.1% (4.1%-10%) | 16% (3.1%-30%) | 0.16 | *0.092* | 0.76 | 0.16 | 1 | 1 | 1 | 1 | No* |
|  | 2 | 15% (10%-19%) | 12% (7.8%-17%) | 24% (13%-35%) | **0.015** | 0.11 | *0.07* | **5.5E-03** | 0.28 | 1 | 1 | 0.19 | No* |
|  | 3 | 16% (10%-23%) | 10% (5.9%-15%) | 35% (19%-50%) | **3.2E-04** | *0.082* | **7.2E-03** | **6.1E-05** | **8.7E-03** | 1 | 0.24 | **3.6E-03** | No* |
|  | 4 | 14% (7.8%-21%) | 13% (7.5%-19%) | 59% (40%-77%) | **1.0E-07** | 0.98 | **5.2E-07** | **1.8E-07** | **5.1E-06** | 1 | **5.0E-05** | **1.9E-05** | No* |
|  | 5 | 13% (6.2%-20%) | 13% (6.7%-20%) | 56% (33%-79%) | **2.4E-04** | 0.92 | **2.1E-04** | **1.8E-04** | **6.8E-03** | 1 | **0.011** | **9.6E-03** | No* |
| fLRI / ARI | 1 | 7.3% (4.5%-10%) | 7.3% (4%-11%) | 15% (7.6%-22%) | **0.025** | 0.43 | **0.028** | **8.8E-03** | 0.43 | 1 | 0.75 | 0.29 | No* |
|  | 2 | 7.9% (4.7%-11%) | 11% (6.9%-15%) | 28% (12%-44%) | **0.014** | 0.69 | **4.3E-03** | **0.01** | 0.26 | 1 | 0.16 | 0.32 | No* |
|  | 3 | 11% (6.1%-16%) | 5% (2.1%-8%) | 13% (2.9%-24%) | *0.066* | *0.05* | 0.6 | *0.058* | 0.97 | 1 | 1 | 1 | No* |
|  | 4 | 7.9% (4%-12%) | 8.1% (2.8%-13%) | 17% (4%-30%) | **0.046** | 0.26 | 0.11 | **0.013** | 0.71 | 1 | 1 | 0.4 | No* |
|  | 5 | 11% (4.4%-17%) | 7% (2.3%-12%) | 7.8% (0%-17%) | 0.68 | 0.38 | 0.74 | 0.86 | 1 | 1 | 1 | 1 | No* |
| sLRI / ARI | 1 | 16% (11%-21%) | 12% (8%-16%) | 26% (12%-40%) | **0.041** | *0.091* | 0.2 | **0.021** | 0.65 | 1 | 1 | 0.6 | No* |
|  | 2 | 19% (14%-24%) | 18% (13%-24%) | 42% (27%-58%) | **2.3E-03** | 0.56 | **1.8E-03** | **7.9E-04** | *0.053* | 1 | *0.074* | **0.035** | No* |
|  | 3 | 21% (14%-27%) | 13% (7.7%-17%) | 36% (21%-51%) | **1.0E-03** | **0.044** | **0.031** | **3.0E-04** | **0.025** | 1 | 0.81 | **0.015** | No* |
|  | 4 | 17% (10%-24%) | 15% (8.7%-21%) | 59% (41%-78%) | **7.8E-07** | 0.67 | **4.0E-06** | **5.6E-07** | **3.4E-05** | 1 | **3.2E-04** | **5.3E-05** | No* |
|  | 5 | 17% (9.4%-25%) | 16% (8.8%-23%) | 56% (33%-79%) | **1.5E-03** | 0.74 | **1.1E-03** | **7.1E-04** | **0.036** | 1 | **0.047** | **0.032** | No* |
| *Proportion of LRI events* |  |  |  |  |  |  |  |  |  |  |  |  |  |
| wLRI / LRI | 1 | 33% (23%-44%) | 28% (17%-38%) | 31% (11%-52%) | 0.54 | 0.27 | 0.82 | 0.63 | 1 | 1 | 1 | 1 | No* |
|  | 2 | 54% (42%-66%) | 39% (28%-50%) | 42% (24%-59%) | 0.16 | *0.068* | 0.25 | 0.57 | 1 | 1 | 1 | 1 | No* |
|  | 3 | 52% (39%-66%) | 52% (38%-67%) | 80% (62%-98%) | *0.099* | 0.95 | **0.035** | *0.059* | 1 | 1 | 0.9 | 1 | No* |
|  | 4 | 49% (33%-65%) | 65% (49%-82%) | 94% (86%-100%) | **1.1E-03** | *0.08* | **2.1E-04** | **0.026** | **0.027** | 1 | **0.011** | 0.71 | No* |
|  | 5 | 62% (42%-82%) | 66% (48%-84%) | 88% (68%-100%) | 0.28 | 0.75 | 0.11 | 0.19 | 1 | 1 | 1 | 1 | No* |
| fLRI / LRI | 1 | 24% (15%-33%) | 25% (15%-35%) | 39% (19%-58%) | 0.21 | 0.81 | 0.11 | 0.11 | 1 | 1 | 1 | 1 | No* |
|  | 2 | 30% (20%-41%) | 36% (26%-47%) | 43% (23%-62%) | 0.54 | 0.74 | 0.23 | 0.43 | 1 | 1 | 1 | 1 | No* |
|  | 3 | 36% (23%-48%) | 32% (18%-45%) | 28% (8.2%-48%) | 0.77 | 0.55 | 0.56 | 0.92 | 1 | 1 | 1 | 1 | No* |
|  | 4 | 31% (18%-45%) | 33% (17%-50%) | 27% (7.4%-47%) | 0.89 | 0.76 | 0.61 | 0.98 | 1 | 1 | 1 | 1 | No* |
|  | 5 | 45% (26%-65%) | 34% (17%-51%) | 18% (0%-40%) | 0.2 | 0.37 | 0.08 | 0.28 | 1 | 1 | 1 | 1 | No* |
| sLRI / LRI | 1 | 50% (39%-61%) | 42% (30%-53%) | 62% (39%-85%) | 0.17 | 0.23 | 0.32 | 0.085 | 1 | 1 | 1 | 1 | No* |
|  | 2 | 68% (58%-79%) | 60% (49%-71%) | 71% (53%-88%) | 0.67 | 0.42 | 0.89 | 0.55 | 1 | 1 | 1 | 1 | No* |
|  | 3 | 67% (55%-80%) | 65% (51%-78%) | 85% (68%-100%) | 0.28 | 0.93 | 0.13 | 0.14 | 1 | 1 | 1 | 1 | No* |
|  | 4 | 61% (45%-77%) | 74% (58%-90%) | 97% (91%-100%) | **3.6E-03** | *0.094* | **8.8E-04** | *0.057* | *0.08* | 1 | **0.038** | 1 | No* |
|  | 5 | 82% (66%-98%) | 77% (61%-92%) | 88% (68%-100%) | 0.67 | 0.57 | 0.75 | 0.42 | 1 | 1 | 1 | 1 | No* |
|  |  | **Prop. (95% CI)** | **Prop. (95% CI)** | **Prop. (95% CI)** | **Overall** | **1 vs. 2** | **1 vs. 3** | **2 vs. 3** | **Overall** | **1 vs. 2** | **1 vs. 3** | **2 vs. 3** |  |
| >20% *Streptococcus* in  first infection-naive NPA | 7w | 11% (0.34%-23%) | 15% (3.3%-26%) | 44% (3.9%-85%) | *0.081* | 0.75 | **0.042** | *0.065* | 1 | 1 | 1 | 1 | No |
|  | 6m | 7.6% (1.6%-14%) | 18% (10%-26%) | 14% (0%-31%) | 0.12 | **0.045** | 0.39 | 1 | 1 | 1 | 1 | 1 | No |
| % Healthy NPAs with  risk-associated MPGs | 0-2 | 49% (38%-59%) | 32% (24%-39%) | 62% (47%-76%) | **1.2E-03** | **0.013** | 0.2 | **5.5E-04** | **0.029** | 0.4 | 1 | **0.025** | No |
|  | 2-4 | 46% (37%-55%) | 44% (37%-51%) | 45% (29%-61%) | 0.9 | 0.67 | 0.92 | 0.8 | 1 | 1 | 1 | 1 | No |
| % Healthy NPAs with  health-associated MPGs | 0-2 | 35% (25%-44%) | 46% (38%-55%) | 29% (13%-46%) | *0.077* | *0.064* | 0.61 | *0.08* | 1 | 1 | 1 | 1 | No |
|  | 2-4 | 33% (26%-41%) | 28% (21%-35%) | 23% (9.6%-36%) | 0.34 | 0.24 | 0.23 | 0.66 | 1 | 1 | 1 | 1 | No |
|  |  | **Mean (95% CI)** | **Mean (95% CI)** | **Mean (95% CI)** | **Overall** | **1 vs. 2** | **1 vs. 3** | **2 vs. 3** | **Overall** | **1 vs. 2** | **1 vs. 3** | **2 vs. 3** |  |
| Quartile of % healthy NPAs  with risk-associated MPGs | 0-2 | 2.4 (2-2.7) | 1.8 (1.6-2.1) | 2.7 (2.1-3.2) | **3.7E-03** | **0.015** | 0.29 | **2.6E-03** | *0.081* | 0.45 | 1 | 0.1 | No |
|  | 2-4 | 2.2 (1.9-2.5) | 2.2 (1.9-2.4) | 2.2 (1.7-2.7) | 0.99 | 0.99 | 0.89 | 0.87 | 1 | 1 | 1 | 1 | No |
| Quartile of % healthy NPAs  with health-associated MPGs | 0-2 | 2.1 (1.9-2.4) | 2.5 (2.2-2.7) | 2.1 (1.5-2.6) | 0.15 | *0.087* | 0.83 | 0.18 | 1 | 1 | 1 | 1 | No |
|  | 2-4 | 2.3 (2-2.5) | 2 (1.8-2.3) | 1.9 (1.3-2.4) | 0.3 | 0.2 | 0.21 | 0.65 | 1 | 1 | 1 | 1 | No |

Feature? = whether variable was used as a clustering feature or not; geom. mean = geometric mean; ARI = acute respiratory infection (lower or upper); LRI = lower respiratory infection; MPG = microbiome profile group; NPA = nasopharyngeal aspirate; prop. = proportion; P-value (adj.) = adjusted P-values (Benjamini-Yekutieli method); URI = upper respiratory infection; 7w = 7 weeks. For categorical variables, associations were tested using Fisher exact test; for continuous variables, Kruskal-Wallis and Mann-Whitney-Wilcoxon. Bold text indicates statistical significance (*p*<0.05); italics indicate near-significance (*p*<0.10). *Not used as clustering features, as these were derived variables; the variables from which they were derived (URI, LRI, wLRI, fLRI) were used instead. ^Not used as clustering feature due to no variation across entire cohort.

Table supplement 4: Repeated-measures ANOVA for selected predictors, in the first three years of life (timepoints at ages 6m, 1, 2, and 3)

|  | **P-value for predictor, following repeated-measures ANOVA within each cluster** | | |
| --- | --- | --- | --- |
| **Predictor** | **CAS1** | **CAS2** | **CAS3** |
| HDM IgE | 0.97 | **8.1E-03** | *0.059* |
| Phadiatop IgE | 0.24 | 0.78 | 0.47 |
| LRI | **0.048** | **2.4E-03** | 0.18 |
| wLRI | **0.011** | **0.015** | 0.97 |
| fLRI | 0.28 | **0.011** | 0.97 |

Table supplement 5: Comparison of the three clusters generated by npEM, with other clustering or classification schemes

1. **npEM vs. atopy as defined by specific IgE or SPT past atopic threshold by age 2**

|  | **Atopic*** | |
| --- | --- | --- |
| **npEM** | No | Yes |
| CAS1 | 39 | 46 |
| CAS2 | 30 | 75 |
| CAS3 | 0 | 22 |

*Any specific IgE ≥ 0.35kU/L, or any SPT ≥ 2mm, at any timepoint less than two years of age

1. **npEM vs. atopy as defined only by specific IgE past atopic threshold by age 2**

|  | **Atopic*** | |
| --- | --- | --- |
| **npEM** | No | Yes |
| CAS1 | 58 | 26 |
| CAS2 | 40 | 65 |
| CAS3 | 0 | 22 |

*Any specific IgE ≥ 0.35kU/L at any timepoint less than two years of age

Table supplement 6: Correlation between Phadiatop vs. allergen-specific IgE and IgG4 in CAS

1. **Phadiatop vs. allergen-specific IgE**

|  |  | **All clusters** | | **CAS1** | | **CAS2** | | **CAS3** | |
| --- | --- | --- | --- | --- | --- | --- | --- | --- | --- |
| **Age (y)** | **Allergen** | **Rho** | **P-value** | **Rho** | **P-value** | **Rho** | **P-value** | **Rho** | **P-value** |
| 6m | HDM | 0.38 | 2.7E-09 | 0.27 | 0.012 | 0.37 | 1.2E-04 | 0.21 | 0.34 |
|  | Cat | 0.37 | 5.3E-09 | 0.37 | 4.6E-04 | 0.38 | 7.3E-05 | 0.34 | 0.12 |
|  | Peanut | **0.67** | **2.1E-32** | **0.57** | **9.0E-09** | **0.5** | **4.7E-08** | **0.77** | **2.9E-05** |
|  | Mould | 0.18 | 5.3E-03 | 0.4 | 1.1E-04 | 0.25 | 0.012 | -1.1E-01 | 0.61 |
|  | Couch grass | 0.19 | 3.3E-03 | 0.33 | 2.2E-03 | 0.23 | 0.017 | -1.4E-02 | 0.95 |
|  | Ryegrass | 0.21 | 9.8E-04 | 0.34 | 1.2E-03 | 0.29 | 3.1E-03 | 0.016 | 0.94 |
| 1 | HDM | 0.52 | 6.3E-18 | 0.28 | 9.1E-03 | 0.29 | 2.4E-03 | 0.35 | 0.11 |
|  | Cat | 0.43 | 8.4E-12 | 0.22 | 0.04 | 0.31 | 1.1E-03 | 0.23 | 0.31 |
|  | Peanut | **0.71** | **6.4E-37** | **0.54** | **1.1E-07** | **0.49** | **6.3E-08** | **0.69** | **3.5E-04** |
|  | Mould | 0.11 | 0.083 | 0.16 | 0.14 | 0.072 | 0.46 | NA | NA |
|  | Couch grass | 0.27 | 3.6E-05 | 0.27 | 0.011 | 0.18 | 0.069 | 0.14 | 0.54 |
|  | Ryegrass | 0.21 | 1.3E-03 | 0.21 | 0.057 | 0.17 | 0.078 | 0.015 | 0.95 |
| 2 | HDM | **0.79** | **7.4E-47** | **0.64** | **2.3E-11** | **0.59** | **5.1E-11** | 0.43 | 0.046 |
|  | Cat | 0.46 | 7.5E-13 | 0.39 | 1.6E-04 | 0.29 | 2.8E-03 | 0.13 | 0.55 |
|  | Peanut | 0.67 | 1.2E-29 | 0.4 | 1.5E-04 | 0.37 | 1.1E-04 | **0.6** | **3.5E-03** |
|  | Mould | 0.2 | 2.4E-03 | 0.11 | 0.29 | 0.21 | 0.032 | 0.13 | 0.57 |
|  | Couch grass | 0.45 | 2.0E-12 | 0.33 | 1.8E-03 | 0.12 | 0.21 | 0.24 | 0.29 |
|  | Ryegrass | NA | NA | NA | NA | NA | NA | NA | NA |
| 3 | HDM | **0.84** | **1.2E-57** | **0.81** | **1.5E-20** | **0.7** | **4.2E-16** | **0.81** | **4.2E-06** |
|  | Cat | 0.45 | 9.0E-12 | 0.5 | 1.5E-06 | 0.23 | 0.019 | 0.022 | 0.92 |
|  | Peanut | 0.61 | 8.3E-23 | 0.56 | 3.7E-08 | 0.26 | 8.8E-03 | 0.44 | 0.039 |
|  | Mould | 0.36 | 7.7E-08 | 0.5 | 1.2E-06 | 0.13 | 0.2 | 0.46 | 0.031 |
|  | Couch grass | 0.51 | 5.0E-15 | 0.5 | 1.3E-06 | 0.19 | 0.052 | 0.42 | 0.049 |
|  | Ryegrass | 0.57 | 1.2E-19 | 0.59 | 3.3E-09 | 0.25 | 0.012 | 0.61 | 2.3E-03 |
| 4 | HDM | **0.88** | **6.1E-63** | **0.79** | **5.7E-18** | **0.83** | **6.5E-24** | **0.85** | **1.1E-06** |
|  | Cat | 0.52 | 3.0E-14 | 0.45 | 4.3E-05 | 0.4 | 9.5E-05 | -8.0E-04 | 1 |
|  | Peanut | 0.58 | 1.3E-18 | 0.54 | 3.1E-07 | 0.33 | 2.0E-03 | 0.38 | 0.09 |
|  | Mould | 0.33 | 4.2E-06 | 0.26 | 0.023 | 0.28 | 8.3E-03 | 0.27 | 0.23 |
|  | Couch grass | 0.57 | 6.5E-18 | 0.48 | 7.5E-06 | 0.35 | 7.1E-04 | 0.4 | 0.075 |
|  | Ryegrass | 0.63 | 1.5E-22 | 0.55 | 2.1E-07 | 0.38 | 2.8E-04 | 0.51 | 0.018 |
| 5* | HDM | **0.93** | **1.1E-73** | **0.88** | **3.3E-23** | **0.9** | **8.0E-30** | **0.98** | **4.8E-12** |
|  | Cat | 0.58 | 1.9E-16 | 0.45 | 1.3E-04 | 0.52 | 1.1E-06 | 0.18 | 0.48 |
|  | Peanut | 0.59 | 2.9E-17 | 0.61 | 3.7E-08 | 0.43 | 7.1E-05 | 0.24 | 0.34 |
|  | Mould | 0.39 | 2.8E-07 | 0.37 | 1.8E-03 | 0.26 | 0.019 | 0.33 | 0.18 |
|  | Couch grass | 0.66 | 5.2E-22 | 0.63 | 8.4E-09 | 0.5 | 3.3E-06 | 0.52 | 0.028 |
|  | Ryegrass | 0.67 | 6.5E-23 | 0.59 | 1.2E-07 | 0.53 | 6.1E-07 | 0.56 | 0.016 |

* Assay used at age 5 was the adult version, not Phadiatop infant.

1. **Phadiatop vs. allergen-specific IgG4**

|  |  | **All clusters** | | **CAS1** | | **CAS2** | | **CAS3** | |
| --- | --- | --- | --- | --- | --- | --- | --- | --- | --- |
| **Age (y)** | **Allergen** | **Rho** | **P-value** | **Rho** | **P-value** | **Rho** | **P-value** | **Rho** | **P-value** |
| 6m | HDM | 0.14 | 0.029 | NA | NA | 0.053 | 0.59 | 0.38 | 0.081 |
|  | Cat | **0.38** | **2.7E-09** | **0.35** | **9.2E-04** | **0.29** | **2.8E-03** | **0.55** | **8.1E-03** |
|  | Peanut | 0.084 | 0.2 | NA | NA | NA | NA | 0.19 | 0.39 |
|  | Mould | 0.064 | 0.33 | 0.18 | 0.1 | -2.7E-02 | 0.78 | 0.28 | 0.21 |
|  | Couch grass | 0.14 | 0.028 | NA | NA | 0.13 | 0.19 | 0.28 | 0.21 |
|  | Ryegrass | 0.1 | 0.12 | NA | NA | NA | NA | 0.28 | 0.21 |
| 1 | HDM | 0.27 | 3.1E-05 | NA | NA | 0.31 | 1.1E-03 | 0.43 | 0.047 |
|  | Cat | **0.43** | **6.1E-12** | **0.29** | **6.1E-03** | **0.44** | **1.8E-06** | **0.58** | **4.5E-03** |
|  | Peanut | 0.22 | 5.8E-04 | 0.13 | 0.25 | 0.078 | 0.42 | 0.52 | 0.014 |
|  | Mould | 0.085 | 0.19 | NA | NA | NA | NA | 0.25 | 0.26 |
|  | Couch grass | 0.3 | 3.2E-06 | 0.11 | 0.33 | 0.36 | 1.7E-04 | 0.44 | 0.04 |
|  | Ryegrass | 0.15 | 0.023 | 0.056 | 0.61 | 0.088 | 0.37 | 0.33 | 0.13 |
| 2 | HDM | 0.4 | 1.4E-09 | 0.057 | 0.6 | **0.26** | **9.0E-03** | 0.013 | 0.95 |
|  | Cat | **0.53** | **1.8E-17** | **0.43** | **3.5E-05** | 0.2 | 0.04 | **0.67** | **6.6E-04** |
|  | Peanut | 0.43 | 3.6E-11 | 0.28 | 9.4E-03 | 0.23 | 0.018 | 0.56 | 7.1E-03 |
|  | Mould | 0.16 | 0.02 | NA | NA | 0.18 | 0.062 | 0.13 | 0.57 |
|  | Couch grass | 0.31 | 3.5E-06 | 0.13 | 0.23 | 0.21 | 0.035 | 0.44 | 0.039 |
|  | Ryegrass | 0.2 | 2.4E-03 | -1.1E-01 | 0.31 | 0.13 | 0.19 | 0.3 | 0.18 |
| 3 | HDM | 0.49 | 2.6E-14 | 0.25 | 0.025 | 0.29 | 2.8E-03 | -1.3E-02 | 0.95 |
|  | Cat | **0.56** | **1.9E-18** | **0.44** | **3.4E-05** | 0.35 | 2.7E-04 | **0.85** | **4.5E-07** |
|  | Peanut | 0.35 | 1.5E-07 | 0.14 | 0.21 | 0.019 | 0.85 | 0.23 | 0.31 |
|  | Mould | 0.22 | 1.4E-03 | 0.14 | 0.22 | 0.36 | 2.1E-04 | 0.15 | 0.49 |
|  | Couch grass | 0.49 | 5.2E-14 | 0.3 | 5.2E-03 | **0.49** | **2.7E-07** | 0.64 | 1.5E-03 |
|  | Ryegrass | 0.24 | 3.5E-04 | NA | NA | 0.18 | 0.071 | 0.042 | 0.85 |
| 4 | HDM | 0.49 | 1.5E-12 | 0.34 | 2.2E-03 | 0.39 | 2.1E-04 | 0.17 | 0.47 |
|  | Cat | **0.58** | **2.0E-18** | **0.51** | **2.0E-06** | 0.45 | 1.3E-05 | 0.62 | 2.7E-03 |
|  | Peanut | 0.35 | 5.4E-07 | 0.19 | 0.087 | 0.19 | 0.072 | 0.61 | 3.4E-03 |
|  | Mould | 0.25 | 6.5E-04 | 0.17 | 0.14 | 0.38 | 2.4E-04 | 0.49 | 0.024 |
|  | Couch grass | 0.56 | 9.6E-17 | 0.44 | 6.7E-05 | **0.6** | **5.1E-10** | **0.69** | **4.8E-04** |
|  | Ryegrass | 0.27 | 1.7E-04 | 0.13 | 0.25 | 0.28 | 8.0E-03 | 0.53 | 0.014 |
| 5* | HDM | 0.56 | 2.1E-15 | 0.37 | 1.6E-03 | 0.53 | 5.7E-07 | 0.81 | 3.9E-05 |
|  | Cat | 0.47 | 1.6E-10 | 0.34 | 3.9E-03 | 0.45 | 2.9E-05 | 0.97 | 1.5E-11 |
|  | Peanut | 0.5 | 2.7E-12 | 0.4 | 6.6E-04 | 0.19 | 0.1 | 0.94 | 4.2E-09 |
|  | Mould | 0.44 | 1.8E-09 | 0.19 | 0.11 | 0.34 | 2.0E-03 | 0.53 | 0.023 |
|  | Couch grass | 0.57 | 8.1E-16 | 0.25 | 0.035 | 0.58 | 2.8E-08 | **0.98** | **3.9E-13** |
|  | Ryegrass | **0.68** | **4.5E-24** | **0.64** | **3.1E-09** | **0.59** | **1.2E-08** | 0.74 | 4.6E-04 |

* Assay used at age 5 was the adult version, not Phadiatop infant.

Table supplement 7: Complete version of Table 4: Predictors for age-five wheeze within each CAS cluster, with demographic covariates (sex, BMI, parental history of asthma)

| **Selected predictors** |  | **CAS1 (N=88)** |  | **CAS2 (N=107)** |  | **CAS3 (N=22)** |  | **All (N=261)** |  |
| --- | --- | --- | --- | --- | --- | --- | --- | --- | --- |
| **for age-five wheeze** |  | **OR (95% CI)** | **P-value** | **OR (95% CI)** | **P-value** | **OR (95% CI)** | **P-value** | **OR (95% CI)** | **P-value** |
| ARI (events per y) | 1 | 1.1 (0.88-1.5) | 0.36 | 1.1 (0.87-1.3) | 0.51 | 0.57 (0.29-0.93) | **0.046** | 1 (0.89-1.2) | 0.76 |
|  | 2 | 1.1 (0.94-1.3) | 0.22 | 1 (0.81-1.3) | 0.82 | 0.43 (0.077-0.89) | 0.12 | 1 (0.93-1.2) | 0.44 |
|  | 3 | 1.1 (0.87-1.3) | 0.58 | 1.1 (0.91-1.4) | 0.3 | 0.67 (0.36-1) | 0.1 | 1 (0.93-1.2) | 0.48 |
|  | 4 | 1.2 (0.99-1.4) | *0.074* | 1.2 (1-1.5) | **0.032** | 0.63 (0.27-1.1) | 0.15 | 1.2 (1-1.3) | **0.013** |
| LRI (events per y) | 1 | 0.97 (0.71-1.3) | 0.84 | 1 (0.61-1.5) | 0.99 | 0.48 (0.13-1.1) | 0.16 | 1 (0.81-1.2) | 0.92 |
|  | 2 | 1.2 (0.88-1.6) | 0.26 | 1.5 (0.97-2.5) | *0.069* | 0.99 (0.34-2.6) | 0.98 | 1.4 (1.1-1.7) | **5.3E-03** |
|  | 3 | 2 (1.3-3.2) | **2.3E-03** | 2.6 (1.5-5.3) | **2.7E-03** | 0.98 (0.4-2.6) | 0.96 | 2 (1.5-2.7) | **3.8E-06** |
|  | 4 | 2 (1.4-3.4) | **2.0E-03** | 3.6 (1.8-8.3) | **6.5E-04** | 1.9 (0.57-8.4) | 0.32 | 2.5 (1.8-3.6) | **1.5E-07** |
| Wheezy LRI (events per y) | 1 | 1.3 (0.68-2.4) | 0.43 | 1.1 (0.35-3) | 0.83 | 2.6 (0.62-58) | 0.34 | 1.5 (0.98-2.3) | *0.06* |
|  | 2 | 1.2 (0.8-2) | 0.33 | 1.6 (0.89-2.9) | 0.12 | 2.4 (0.67-16) | 0.24 | 1.6 (1.2-2.2) | **5.6E-03** |
|  | 3 | 2.8 (1.6-5.6) | **1.3E-03** | 3 (1.4-8) | **0.016** | 1.2 (0.43-4.6) | 0.76 | 2.7 (1.8-4.2) | **4.1E-06** |
|  | 4 | 2.5 (1.5-5) | **4.0E-03** | 6.3 (2.5-21) | **6.8E-04** | 7.1 (1.2-169) | 0.1 | 3.9 (2.5-6.7) | **5.4E-08** |
| Febrile LRI (events per y) | 1 | 1.6 (0.77-3.6) | 0.21 | 0.84 (0.28-1.9) | 0.71 | 7.3 (0.78-178) | 0.12 | 1.5 (0.93-2.4) | *0.098* |
|  | 2 | 1 (0.44-2.2) | 1 | 4.8 (1.8-15) | **3.9E-03** | 1.6 (0.48-10) | 0.5 | 2.3 (1.4-3.9) | **1.2E-03** |
|  | 3 | 2 (1-4.8) | *0.08* | 4.3 (1.2-15) | **0.02** | 4.2 (0.55-519) | 0.37 | 2.4 (1.4-4.3) | **2.3E-03** |
|  | 4 | 1.8 (0.97-4.1) | *0.092* | 2.6 (0.88-8.3) | *0.082* | 1.1 (0.11-18) | 0.93 | 2.2 (1.3-4) | **5.9E-03** |
| % Healthy NPAs with infection-associated MPGs | 0-2 | 0.9 (0.13-5.7) | 0.91 | 2.6 (0.43-16) | 0.3 | NA | NA | 2.3 (0.79-6.7) | 0.13 |
|  | 2-4 | 0.086 (6.8E-03-0.71) | **0.034** | 0.8 (0.077-7.5) | 0.85 | 4.4E+03 (2.1-2.5E+12) | 0.13 | 0.49 (0.14-1.6) | 0.24 |
| Quartile of % healthy NPAs with infection-associated MPGs | 0-2 | 1 (0.54-1.8) | 0.98 | 1.3 (0.72-2.4) | 0.36 | NA | NA | 1.3 (0.89-1.8) | 0.19 |
|  | 2-4 | 0.45 (0.19-0.88) | **0.035** | 1 (0.51-2.1) | 0.9 | NA | NA | 0.8 (0.53-1.2) | 0.24 |
| HDM IgE (kU/L)* | 6m | 8 (0.85-94) | *0.074* | 0.93 (0.14-3.6) | 0.92 | 3.4 (0.26-180) | 0.4 | 2.3 (0.99-5.8) | *0.054* |
|  | 1 | 1.5 (0.22-7.8) | 0.65 | 0.54 (0.039-2.3) | 0.51 | 39 (2.5-22000) | *0.082* | 2.7 (1.5-5) | **0.00089** |
|  | 2 | 0.93 (0.28-2.5) | 0.89 | 2 (1.2-3.7) | **0.016** | 1.4 (0.38-4.8) | 0.62 | 2 (1.5-2.8) | **2.80E-05** |
|  | 3 | 1.4 (0.68-2.9) | 0.32 | 1.5 (0.9-2.4) | 0.12 | 1.5 (0.4-5.2) | 0.55 | 1.7 (1.3-2.2) | **1.00E-04** |
|  | 4 | 1.9 (0.94-4.1) | *0.086* | 1.9 (1.2-3.1) | **0.011** | 1.4 (0.31-5.5) | 0.64 | 1.9 (1.5-2.5) | **3.70E-06** |
| Peanut IgE (kU/L)* | 6m | 2.5 (0.78-9) | 0.13 | 1.5 (0.54-3.8) | 0.41 | 1.1 (0.3-3.7) | 0.92 | 2.3 (1.4-3.9) | **0.0014** |
|  | 1 | 1.7 (0.48-6.3) | 0.39 | 2.2 (0.65-6.9) | 0.19 | 0.47 (0.095-1.6) | 0.27 | 2.2 (1.4-3.6) | **0.00098** |
|  | 2 | 0.51 (0.097-2) | 0.37 | 3 (0.74-12) | 0.12 | 2 (0.51-13) | 0.37 | 2.7 (1.6-4.9) | **0.00046** |
|  | 3 | 1.7 (0.46-5.5) | 0.37 | 0.53 (0.015-3.8) | 0.61 | 3.3 (0.94-26) | 0.13 | 2.6 (1.6-4.8) | **0.00068** |
|  | 4 | 0.2 (0.00073-2.9) | 0.36 | 0.96 (0.19-3.2) | 0.95 | 1.4 (0.49-6.5) | 0.54 | 2.1 (1.3-3.7) | **0.006** |
| Cat IgE (kU/L)* | 6m | 6.6 (0.77-61) | *0.079* | 2.2 (0.62-7.6) | 0.2 | 0.24 (0.012-3.2) | 0.29 | 2.3 (0.96-5.4) | *0.061* |
|  | 1 | 2.1 (0.13-30) | 0.57 | 4 (0.54-32) | 0.16 | 0.45 (0.053-2.8) | 0.41 | 3.5 (1.4-9.5) | **0.0099** |
|  | 2 | 0.55 (0.042-3.7) | 0.57 | 2.1 (0.59-7) | 0.22 | 2.2 (0.42-26) | 0.42 | 2.6 (1.3-5.5) | **0.0065** |
|  | 3 | 1.7 (0.49-5.6) | 0.35 | 1.4 (0.21-6.7) | 0.66 | 1.3 (0.29-6.9) | 0.77 | 2.5 (1.3-4.9) | **0.0065** |
|  | 4 | 0.75 (0.0088-13) | 0.86 | 1.5 (0.53-3.9) | 0.4 | 0.83 (0.17-4.4) | 0.81 | 2.4 (1.3-4.8) | **0.006** |
| Couch grass IgE (kU/L)* | 6m | 2.8 (0.51-14) | 0.21 | 1.3 (0.3-4.5) | 0.68 | 0.98 (0.048-59) | 0.99 | 1.7 (0.71-3.9) | 0.22 |
|  | 1 | 0.38 (0.017-2.8) | 0.42 | 0.33 (0.01-2.9) | 0.41 | 0.15 (0.0058-1.5) | 0.14 | 0.63 (0.19-1.7) | 0.4 |
|  | 2 | 0.085 (0.0034-0.7) | *0.057* | 1.1 (0.14-6.3) | 0.9 | 25 (1.6-1100) | **0.046** | 2.1 (0.99-4.7) | *0.053* |
|  | 3 | 2 (0.44-8) | 0.29 | 6.1e-06 (NA-8.1e+54) | 0.99 | 2.3 (0.57-14) | 0.29 | 2.5 (1.3-5.1) | **8.90E-03** |
|  | 4 | 8.4e-13 (NA-3.5e+172) | 0.99 | 1.6 (0.55-4.1) | 0.34 | 1.9 (0.54-10) | 0.35 | 2 (1.3-3.4) | **4.30E-03** |
| Phadiatop IgE (PAU/L)* | 6m | 1.2 (0.44-2.9) | 0.73 | 1.3 (0.65-2.6) | 0.43 | 2.2 (0.66-12) | 0.25 | 2 (1.3-2.9) | **0.00078** |
|  | 1 | 0.73 (0.2-2.5) | 0.63 | 1.1 (0.41-2.8) | 0.85 | 1.6 (0.23-18) | 0.67 | 2.1 (1.3-3.4) | **0.0021** |
|  | 2 | 0.33 (0.091-1) | *0.065* | 2.1 (0.81-5.9) | 0.13 | 2.5 (0.18-70) | 0.52 | 2 (1.3-3) | **0.0012** |
|  | 3 | 1.8 (0.8-4) | 0.16 | 1.4 (0.72-2.8) | 0.31 | 8.4 (0.53-380) | 0.19 | 2 (1.4-2.9) | **8.00E-05** |
|  | 4 | 1.8 (0.91-3.8) | *0.094* | 2.4 (1.3-4.8) | **0.01** | 2.7 (0.16-66) | 0.5 | 2.2 (1.6-3.2) | **2.20E-06** |
| HDM IgG4 (μg/L)* | 6m | NA (NA-NA) | 0.55 | 0.053 (NA-6.5e+24) | 0.99 | 28 (1.7e-34-NA) | 0.99 | 1.4 (0.88-2.6) | 0.17 |
|  | 1 | NA (NA-NA) | 0.61 | 1.1 (0.8-1.5) | 0.5 | 0.9 (0.58-1.3) | 0.6 | 1.2 (1-1.4) | *0.053* |
|  | 2 | 1.1 (0.71-1.6) | 0.67 | 1.1 (0.85-1.4) | 0.61 | 0.4 (0.038-1.2) | 0.26 | 1.1 (1-1.3) | *0.056* |
|  | 3 | 1.1 (0.85-1.5) | 0.35 | 1.1 (0.77-2) | 0.64 | 0.94 (0.19-2.3) | 0.9 | 1.1 (0.98-1.2) | 0.1 |
|  | 4 | 1.2 (0.98-1.5) | *0.082* | 0.89 (0.7-1.1) | 0.33 | 0.46 (0.031-5.4) | 0.53 | 1.1 (1-1.3) | **0.034** |
| Peanut IgG4 (μg/L)* | 6m | NA (NA-NA) | 0.55 | NA (NA-NA) | 0.53 | 0.9 (0.42-1.9) | 0.76 | 1.5 (0.94-2.6) | 0.1 |
|  | 1 | 0.075 (NA-3.5e+23) | 0.99 | 0.89 (0.67-1.1) | 0.35 | 0.96 (0.64-1.4) | 0.84 | 1.1 (0.95-1.2) | 0.22 |
|  | 2 | 1.1 (0.85-1.3) | 0.54 | 0.96 (0.8-1.2) | 0.64 | 0.89 (0.48-1.4) | 0.65 | 1 (0.95-1.2) | 0.37 |
|  | 3 | 1.1 (0.89-1.4) | 0.37 | 1 (0.83-1.3) | 0.87 | 0.68 (0.22-1.3) | 0.37 | 1.1 (0.96-1.2) | 0.27 |
|  | 4 | 1.1 (0.92-1.4) | 0.22 | 0.91 (0.76-1.1) | 0.35 | 0.73 (0.19-1.4) | 0.45 | 1.1 (0.96-1.2) | 0.24 |
| Cat IgG4 (μg/L)* | 6m | 0.057 (NA-2e+12) | 0.99 | 0.99 (0.67-1.3) | 0.95 | 24 (3.3e-30-NA) | 1 | 1.1 (0.88-1.3) | 0.41 |
|  | 1 | 0.76 (0.43-1.1) | 0.22 | 0.94 (0.78-1.1) | 0.54 | 0.76 (0.42-1.2) | 0.28 | 1 (0.9-1.1) | 0.82 |
|  | 2 | 1.4 (1.1-1.7) | **0.011** | 0.92 (0.67-1.3) | 0.59 | 0.96 (0.51-1.6) | 0.88 | 1.1 (1-1.3) | *0.053* |
|  | 3 | 1.3 (1-1.6) | *0.05* | 0.9 (0.63-1.4) | 0.59 | 0.86 (0.054-13) | 0.91 | 1.2 (1-1.4) | **0.033** |
|  | 4 | 1.4 (1.1-2) | **0.027** | 0.89 (0.64-1.3) | 0.49 | 0.54 (0.011-1.5) | 0.58 | 1.2 (1-1.5) | **0.034** |
| Couch grass IgG4 (μg/L)* | 6m | NA (NA-NA) | 0.55 | 0.062 (NA-1.3e+24) | 0.99 | 19 (2.5e-57-NA) | 1 | 1.3 (0.74-2.4) | 0.32 |
|  | 1 | 0.081 (NA-9.7e+23) | 0.99 | 1 (0.77-1.3) | 0.81 | 0.93 (0.6-1.4) | 0.71 | 1.1 (0.92-1.3) | 0.29 |
|  | 2 | 0.071 (NA-2.1e+22) | 0.99 | 0.88 (0.7-1.1) | 0.22 | 0.91 (0.61-1.3) | 0.61 | 1 (0.88-1.1) | 0.96 |
|  | 3 | 1.2 (0.99-1.6) | *0.061* | 0.85 (0.7-1) | 0.1 | 1.4 (0.88-2.2) | 0.16 | 1.1 (0.96-1.2) | 0.22 |
|  | 4 | 1.1 (0.91-1.4) | 0.28 | 0.72 (0.56-0.91) | **0.0074** | 0.88 (0.24-1.9) | 0.75 | 1 (0.91-1.2) | 0.69 |
| Phadiatop Infant IgG4 (PAU/L)* | 6m | 0.7 (0.45-0.91) | **0.03** | 1 (0.88-1.2) | 0.79 | 1.4 (0.96-2.4) | 0.12 | 0.98 (0.89-1.1) | 0.67 |
|  | 1 | 0.91 (0.72-1.2) | 0.4 | 0.73 (0.49-0.99) | *0.057* | 0.83 (0.29-1.5) | 0.64 | 0.93 (0.81-1.1) | 0.35 |
|  | 2 | 1.1 (0.89-1.3) | 0.49 | 0.97 (0.68-1.6) | 0.86 | 1.7 (0.93-7.7) | 0.2 | 1.1 (0.96-1.3) | 0.2 |
|  | 3 | 2.3 (1.1-6.8) | *0.091* | 0.23 (0.071-0.64) | **0.0076** | 1 (0.17-7.3) | 1 | 1.3 (0.96-1.8) | 0.16 |
|  | 4 | 1 (0.83-1.4) | 0.71 | 0.3 (0.097-0.85) | **0.028** | 0.42 (0.042-3.2) | 0.4 | 1.1 (0.88-1.3) | 0.61 |
| HDM IgG (mg/L)* | 1 | 25 (0.32-1.6E+04) | 0.19 | 3.3 (0.16-46) | 0.38 | 5.6E-03 (8.4E-06-0.57) | *0.058* | 2 (0.31-11) | 0.44 |
|  | 2 | 0.8 (0.15-3.5) | 0.78 | 0.97 (0.24-3.7) | 0.96 | 0.79 (0.031-18) | 0.88 | 1.3 (0.6-2.9) | 0.48 |
|  | 3 | 2.3 (0.14-35) | 0.54 | 0.48 (0.057-2.5) | 0.43 | 3.9 (0.26-96) | 0.34 | 2.1 (0.89-5) | *0.089* |
| Cat IgG (mg/L)* | 1 | 1.5E-15 (NA-1.2E+291) | 0.99 | 6.5 (0.22-150) | 0.24 | 4.6E-03 (1.4E-06-0.9) | *0.082* | 1.7 (0.11-18) | 0.68 |
|  | 2 | 0.66 (0.077-3.5) | 0.65 | 1.2 (0.28-4.3) | 0.82 | 0.16 (4.0E-03-3.5) | 0.26 | 0.87 (0.34-2.1) | 0.75 |
|  | 3 | 0.023 (8.2E-06-2) | 0.18 | 0.52 (0.058-2.7) | 0.49 | 3.7 (0.18-244) | 0.44 | 1.1 (0.35-3) | 0.9 |

BMI = body mass index; HDM = house dust mite; LRI = lower respiratory infection. Association analyses performed via generalised linear models (GLM) with demographic covariates: age-five wheeze ~ predictor + sex (male) + BMI at age 3 + paternal history of asthma + maternal history of asthma. Bold text indicates statistical significance (*p*<0.05); italics indicate near-significance (*p*<0.10). *Odds ratio (OR) is for every 10-fold increase in IgE, IgG4 or IgG.
